# Transformable Gel‐to‐Nanovaccine Enhances Cancer Immunotherapy via Metronomic‐Like Immunomodulation and Collagen‐Mediated Paracortex Delivery

**DOI:** 10.1002/adma.202409914

**Published:** 2024-10-09

**Authors:** Seung Mo Jin, Ju Hee Cho, Yejin Gwak, Sei Hyun Park, Kyungmin Choi, Jin‐Ho Choi, Hong Sik Shin, JungHyub Hong, Yong‐Soo Bae, Jaewon Ju, Mikyung Shin, Yong Taik Lim

**Affiliations:** ^1^ SKKU Advanced Institute of Nanotechnology (SAINT) Department of Nano Engineering, Department of Nano Science and Technology, School of Chemical Engineering, and Biomedical Institute for Convergence at SKKU Sungkyunkwan University 2066 Seobu‐ro, Jangan‐gu Suwon Gyeonggi‐do 16419 Republic of Korea; ^2^ Progeneer 12 Digital‐ro 31‐gil, Guro‐gu Seoul 08380 Republic of Korea; ^3^ Department of Biological Sciences Science Research Center (SRC) for Immune Research on Non‐lymphoid Organ (CIRNO) Sungkyunkwan University 2066 Seobu‐ro, Jangan‐gu Suwon Gyeonggi‐do 16419 Republic of Korea; ^4^ Department of Biomedical Engineering Center for Neuroscience Imaging Research Institute for Basic Science (IBS) Department of Intelligent Precision Healthcare Convergence Sungkyunkwan University 2066 Seobu‐ro, Jangan‐gu Suwon Gyeonggi‐do 16419 Republic of Korea

**Keywords:** bioadhesive immune niche, cancer immunotherapy, dynamic immune modulation, lymph node delivery, metronomic immunotherapy, nanovaccine

## Abstract

The generation of non‐exhausted effector T‐cells depends on vaccine's spatiotemporal profile, and untimely delivery and low targeting to lymph node (LN) paracortex by standard bolus immunization show limited efficacy. By recapitulating the dynamic processes of acute infection, a bioadhesive immune niche domain (BIND) is developed that facilitates the delivery of timely‐activating conjugated nanovaccine (t‐CNV) in a metronomic‐like manner and increased the accumulation and retention of TANNylated t‐CNV (tannic acid coated t‐CNV) in LN by specifically binding to collagen in subcapsular sinus where they gradually transformed into TANNylated antigen–adjuvant conjugate by proteolysis, inducing their penetration into paracortex through the collagen‐binding in LN conduit and evoking durable antigen‐specific CD8^+^ T‐cell responses. The BIND combined with t‐CNV, mRNA vaccine, IL‐2, and anti‐PD‐1 antibody also significantly enhanced cancer immunotherapy by the dynamic modulation of immunological landscape of tumor microenvironment. The results provide material design strategy for dynamic immunomodulation that can potentiate non‐exhausted T‐cell‐based immunotherapy.

## Graphical abstract

A bioadhesive immune niche domain (BIND) is developed that delivers timely‐activating conjugated nanovaccine (t‐CNV) in a metronomic‐like manner, enhancing its accumulation and retention in lymph nodes. By binding to collagen in the subcapsular sinus, t‐CNV gradually transforms into an antigen–adjuvant conjugate, penetrates the paracortex through conduit, and triggers durable antigen‐specific CD8^+^ T‐cell responses.

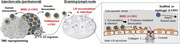

## 1 Introduction

The efficient generation of antigen‐specific effector CD8^+^ T cells in lymphoid organs such as the lymph node (LN) and spleen plays a pivotal role in the cancer‐immunity cycle.^[^ ^1^, ^2^, ^3^, ^4^, ^5^ ^]^ However, cancer vaccines administered via standard bolus immunization that are used to activate innate immune cells and generate antigen‐specific T cells in the LN generally have limited efficacy due to their untimely delivery, low targeting of the LN paracortex, and induction of immune tolerance, which limits their effectiveness due to refractoriness to further stimulation.^[^ ^6^, ^7^ ^]^ To counteract T‐cell‐based immunotherapies, tumors also employ immune escape mechanisms, including the establishment of an immunosuppressive tumor microenvironment (TME) and the induction of T‐cell exhaustion, suggesting that neither the tumor itself nor the TME remains static.^[^ ^8^, ^9^ ^]^

Biomaterials can play a pivotal role in allowing fine‐tuned dynamic control over the dosage, timing, and location of immunomodulation at both the cellular and tissue levels.^[^ ^6^, ^10^, ^11^, ^12^, ^13^, ^14^ ^]^ Hydrogels, scaffolds, and microparticles have been designed to recruit and program innate immune cells at the administration site, particularly within the TME, establishing a lasting presence for an extended period in the local environment.^[^ ^15^, ^16^, ^17^, ^18^, ^19^, ^20^, ^21^, ^22^ ^]^ However, conventional vaccines released from these materials or passively targeted engineered nanoparticle vaccines often exhibit low retention and penetration into the LN paracortex region, leading to the limited generation of antigen‐specific T‐cell responses.^[^ ^6^, ^13^ ^]^

**Figure 1:**
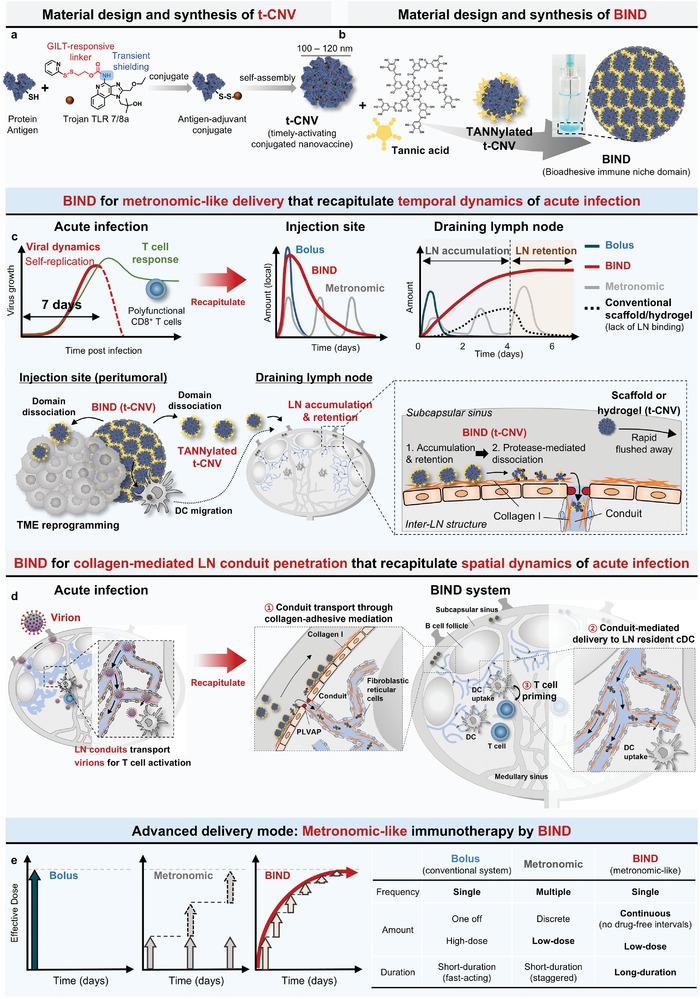
Multiscale dynamic immune modulation with the timely‐activating conjugated nanovaccine (t‐CNV) and the bioadhesive immune niche domain (BIND) augments antitumor immunity. a) Material design and synthesis of t‐CNV. b) Material design and synthesis of BIND. c) BIND for metronomic‐like immunotherapy that recapitulates the temporal dynamic (viral dynamics of self‐replication) of acute infection. d) BIND for collagen‐mediated LN conduit penetration that recapitulates the spatial dynamic (migration toward the T‐cell zone via LN conduits) of acute infection. e) Comparison of vaccine delivery modes and their effects on drug action kinetics and dynamic immune modulation.

Here, we suggest a strategy for the generation of effector CD8^+^ T cells in the LN that mimics the dynamic process of acute infection,^[^ ^23^, ^24^, ^25^, ^26^, ^27^ ^]^ where naïve T cells engage with specific antigens and danger signals for six to seven days and activated T cells are generated for pathogen clearance after the entry of pathogens into LN conduit. We hypothesize that recapitulating the spatiotemporal dynamics of acute infection in LN lead to the design of transformable LN‐adaptive nanovaccine that can be positioned into LN by metronomic‐like delivery and time‐dependent accumulation by targeting collagen, finally transforming into small‐sized antigen‐adjuvant conjugates that facilitate the penetration into paracortex through the LN conduit and provoke enduring antigen‐specific CD8^+^ T‐cell responses (**Figure** **1**). To devise an immunomodulatory drug with metronomic‐like kinetically controlled delivery that targets collagen, we developed the bioadhesive immune niche domain (BIND) by the in situ physical crosslinking of a timely‐activating conjugated nanovaccine (t‐CNV) (synthesized by the conjugation of protein antigen with Trojan TLR7/8a to form nanoparticulate structures through self‐assembly) with the bioadhesive tannic acid (TA) (Figure 1a,b). This allows the controlled and programmed delivery of a low dose of TANNylated t‐CNV during the domain dissociation process into the TME and LN and induces its accumulation and retention in the LN by binding with the collagen that exists in the subcapsular sinus (SCS) and LN conduit (Figure 1c). We demonstrated that TA plays a key role in implementing the metronomic‐like delivery kinetics for TANNylated t‐CNV, enhancing resistance to proteolysis, promoting stability, and allowing prolonged attachment in the SCS. Consequently, due to the high levels of protease in the SCS, TANNylated t‐CNV remains exposed for an extended period, gradually and continuously transitioning into small‐sized TANNylated protein–adjuvant conjugates. Due to their small size and collagen binding affinity, they can penetrate the paracortex through the LN conduit, where they are taken up by cDC1s, interact with CD8^+^ T cells in the T‐cell zone, and provoke enduring antigen‐specific CD8^+^ T‐cell responses (Figure 1d). While both conventional metronomic therapy and metronomic‐like approaches recapitulated by BIND share the same unified framework of modulating drug–immune cell interactions via the delivery of low doses of immunomodulatory drugs in a time‐dependent manner, BIND offers continuous and sustained metronomic‐like immunomodulation without drug‐free intervals with just a single injection (Figure 1e). The results here demonstrate that metronomic‐like immunomodulation and collagen binding in the LN conduit facilitate efficient delivery of the nanovaccine to the paracortical region of the LN by BIND (t‐CNV) and potentiate durable antigen‐specific effector CD8^+^ T‐cell responses. Furthermore, we demonstrate that in vivo, BIND also modulates the dynamic immunological landscape of the immunosuppressive TME by reprogramming it, reducing the population of myeloid‐derived suppressor cells (MDSCs) and M2 macrophages, reducing the expression of exhaustion markers (PD‐1, LAG‐3, and TIM‐3) on CD8^+^ T cells, and increasing the populations of activated CD8^+^ T cells and NK cells that secrete IFN‐*γ*, TNF‐*α*, and GrB.

## 2 Results and Discussion

### 2.1 t‐CNV Generates Non‐Tolerogenic and Activated Immune Cells

**Figure 2:**
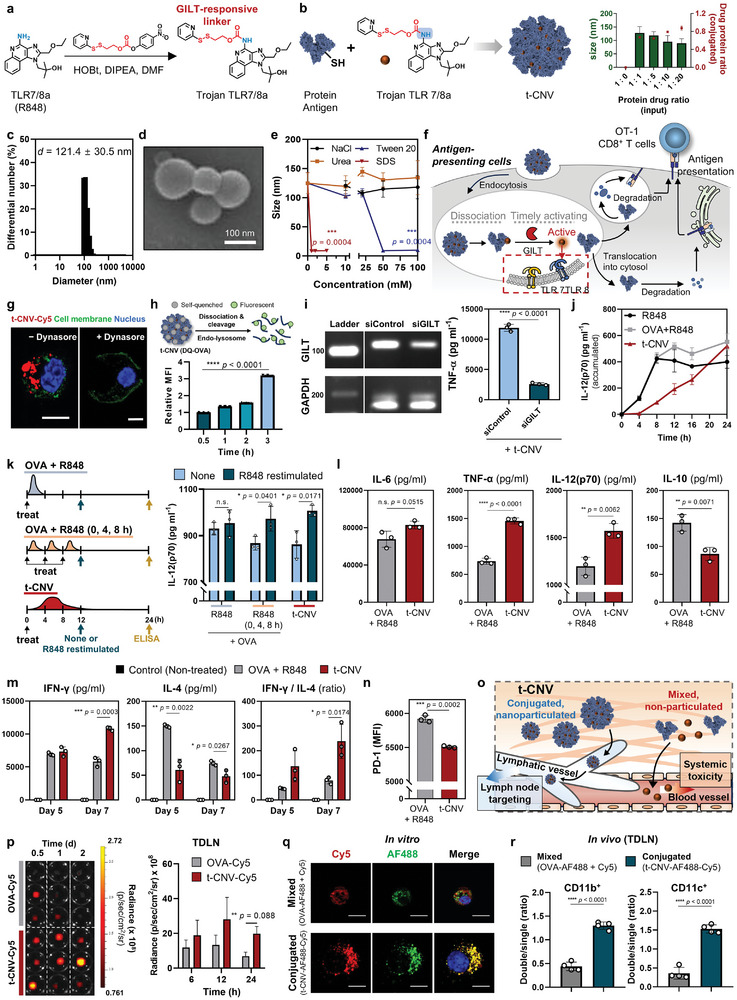
Physiochemical characterization of t‐CNV and its ability to perform dynamic immune modulation at the molecular scale level. a) Schematic of the chemical synthesis of Trojan TLR7/8a. b) Schematic of the synthesis of t‐CNV and its nanoparticulate formulation based on the protein‐to‐drug ratio. c) Dynamic light scattering analysis of t‐CNV particle size distribution. d) Representative scanning electron micrograph of the structure of t‐CNV. Scale bar, 100 nm. e) Dominant mode of interaction during t‐CNV self‐assembly. Tween 20 and SDS are hydrophobic competitors (n = 3). f) Schematic of the mode of action of dynamic immune modulation by t‐CNV at the cellular level. g) Representative image of the endocytosis‐mediated cellular uptake of t‐CNV by BMDCs verified by dynasore (an endocytosis inhibitor). Scale bar, 10 µm. h) Time‐dependent intracellular dissociation and cleavage of the DQ(OVA) protein by t‐CNV following treatment of BMDCs as determined by the measured fluorescence intensity of DQ‐OVA (n = 3). i) GILT‐dependent RAW 264.7 cell activation by t‐CNV (n = 3). t‐CNV was added to siControl‐ or siGILT‐treated RAW 264.7 cells for 36 h. The amount of TNF‐*α* produced was analyzed by ELISA (n = 3). j) Time‐dependent IL‐12(p70) secretion by BMDCs after treatment with R848, OVA+R848, or t‐CNV (OVA, 6.2 µm; R848, 4.5 µm) (n = 3). k) TLR tolerance in BMDCs. BMDCs were stimulated for 12 h with the indicated samples and then restimulated or not with R848 (1 µg mL^−1^). The amount of cytokines produced was analyzed 24 h after the initial sample treatment by ELISA (n = 3). l) Concentrations of proinflammatory cytokines (IL‐6, TNF‐*α*, and IL‐12(p70)) and an anti‐inflammatory cytokine (IL‐10) in BMDCs 36 h after treatment with OVA+R848 or t‐CNV (n = 3). m) Th1 (IFN‐*γ*‐secreting) or Th2 (IL‐4‐secreting) polarization of naïve CD4^+^ T cells cocultured for 5 or 7 days with BMDCs treated with OVA+R848 or t‐CNV for 12 h (n = 3). n) PD‐1 expression level in exhausted OT‐1 CD8^+^ T cells after coculture with BMDCs and OVA+R848 or t‐CNV for 24 h (n = 3). o) Antigen adjuvant‐conjugated and nanoparticle formulations of t‐CNV for targeted delivery to the draining lymph node (dLNs). p) LN‐targeting ability of t‐CNV. IVIS images and average radiance of fluorescent signal in the tumor draining lymph node (TDLN) over time after peritumoral administration of OVA‐Cy5 or t‐CNV‐Cy5 (n = 3). q) In vitro codelivery of a protein antigen (AF488) and small molecule (Cy5). Representative confocal images of the protein and its colocalization with mixed or conjugated small molecules in BMDCs after 4 h of treatment. Hoechst 33342 (blue). Scale bars, 10 µm. r) In vivo codelivery of a protein antigen (AF488) and small molecule (Cy5). Double and single uptake rates by cells in the TDLN 12 h after peritumoral administration of a mixture or the conjugated protein and small molecule (n = 4). All data are presented as the mean ± SD. Statistical significance was evaluated by one‐way ANOVA with Tukey's multiple comparison test in h and by an unpaired two‐tailed *t* test in e, i, k‐n, p and r. *P* values: NS, not significant; *P < 0.05, **P < 0.01, ***P < 0.001, ****P < 0.0001.

To achieve dynamic immune modulation at the cellular level, we conjugated Trojan TLR7/8a with a protein antigen (ovalbumin (OVA)) (Figures 1a and **2**a,b; Figures S1–S6, Supporting Information). Leveraging the unique reactivity of Cys367 amongst the four cysteine residues in OVA,^[^ ^28^ ^]^ OVA was chemically conjugated to Trojan TLR7/8a, which features an active site masked by a gamma‐interferon‐inducible lysosomal thiol reductase (GILT)‐cleavable linker and terminates with 2‐pyridyl disulfide, enabling easy conjugation to cysteine‐containing protein antigens.^[^ ^29^, ^30^ ^]^ The OVA‐Trojan TLR7/8a conjugates formed supramolecular nanoparticle structures (t‐CNV) via desolvating agent (t‐butanol and ethanol)^[^ ^31^ ^]^ and increased hydrophobicity due to conjugation with hydrophobic drug (Trojan TLR7/8a) (Figure 2b). The spherical morphology and size distribution (100–120 nm) of t‐CNV were observed by scanning electron microscopy (SEM) and dynamic light scattering (DLS) (Figure 2c,d; Figures S7 and S8, Supporting Information). We observed that the nanoparticulate structure of t‐CNV was effectively dissociated by Tween 20 and sodium dodecyl sulfate (SDS), suggesting that t‐CNV primarily self‐assembled through hydrophobic interactions (Figure 2e).

The endocytosis‐mediated internalization of t‐CNV by antigen‐presenting cells (APCs) was observed by fluorescence microscopy of bone marrow‐derived dendritic cells (BMDCs) after treatment with t‐CNV in the presence or absence of the endocytosis inhibitor dynasore (Figure 2f,g; Figure S9, Supporting Information). Upon internalization, the protein structure of t‐CNV(DQ‐OVA) rapidly dissociated due to proteolysis in the endolysosome in an ovalbumin degradation assay (DQ‐OVA) (Figure 2f,h). When GILT‐knockdown RAW 264.7 cells were treated with t‐CNV, the production of TNF‐α was significantly reduced, which suggested that the Trojan TLR7/8a portion of t‐CNV was cleaved after exposure to GILT in the endolysosomes, resulting in the activation of TLR7/8a (Figure 2f,i). It was also confirmed that t‐CNV could cross‐present to OT‐1 CD8^+^ T cells (Figure S10, Supporting Information). The intracellular exposure and cleavage of the GILT‐responsive linker Trojan TLR7/8a following endocytosis revealed that DCs treated with t‐CNV exhibited time‐dependent activation (Figures 1a and 2j).^[^ ^32^ ^]^ While R848‐treated BMDCs secreted IL‐12(p70) for only 8–12 h before becoming exhausted, t‐CNV‐treated BMDCs exhibited sustained IL‐12(p70) secretion for more than 24 h. We found that BMDCs treated with a single bolus of R848 failed to respond to secondary R848 stimulation 12 h after the initial treatment (Figure 2k). However, BMDCs treated with R848 in a metronomic, fractionated manner (with three administrations spaced 4 h apart) and those treated with t‐CNV remained responsive to secondary R848 stimulation, suggesting that these treatments did not induce TLR tolerance (Figure 2k). Remarkably, by circumventing TLR tolerance, t‐CNV‐treated BMDCs produced more proinflammatory cytokines (TNF‐*α* and IL‐12(p70)), while no significant difference in IL‐6 levels, but secreted less of the anti‐inflammatory cytokine IL‐10 than R848‐treated BMDCs (Figure 2l). These observations imply that durable TLR stimulation in a fractionated (metronomic) manner and t‐CNV (metronomic‐like) can maintain the long‐lasting effectiveness of APCs without triggering TLR tolerance, thereby maximizing the potency of TLR stimulation. Long‐lasting IL‐12(p70) production by t‐CNV‐treated BMDCs was shown to effectively promote the Th1 polarization of naïve CD4^+^ T cells and prevent further exhaustion of exhausted CD8^+^ T cells (Figure 2m,n).^[^ ^32^, ^33^ ^]^

Compared with soluble protein antigen, the nanoparticulate structure of t‐CNV induced twofold greater localization to the tumor draining lymph node (TDLN) within 6–24 h after peritumoral injection (Figure 2o,p). Moreover, t‐CNV was localized within identical single cells both in in vitro BMDCs (Figure 2q; Figure S11, Supporting Information) and in vivo in the TDLN after peritumoral injection compared to the OVA and small molecule mixture (Figure 2r; Figure S12, Supporting Information). Additionally, we confirmed that t‐CNV had enhanced in vivo antitumor efficacy compared to the nonconjugated/nonparticulated (OVA and R848 mixture) and nonconjugated/particulated (OVA and liposomal TLR7/8a mixture) formulations (Figure S13, Supporting Information).

### 2.2 Characterization of BIND for Dynamic Immune Modulation at the Tissue Level

**Figure 3:**
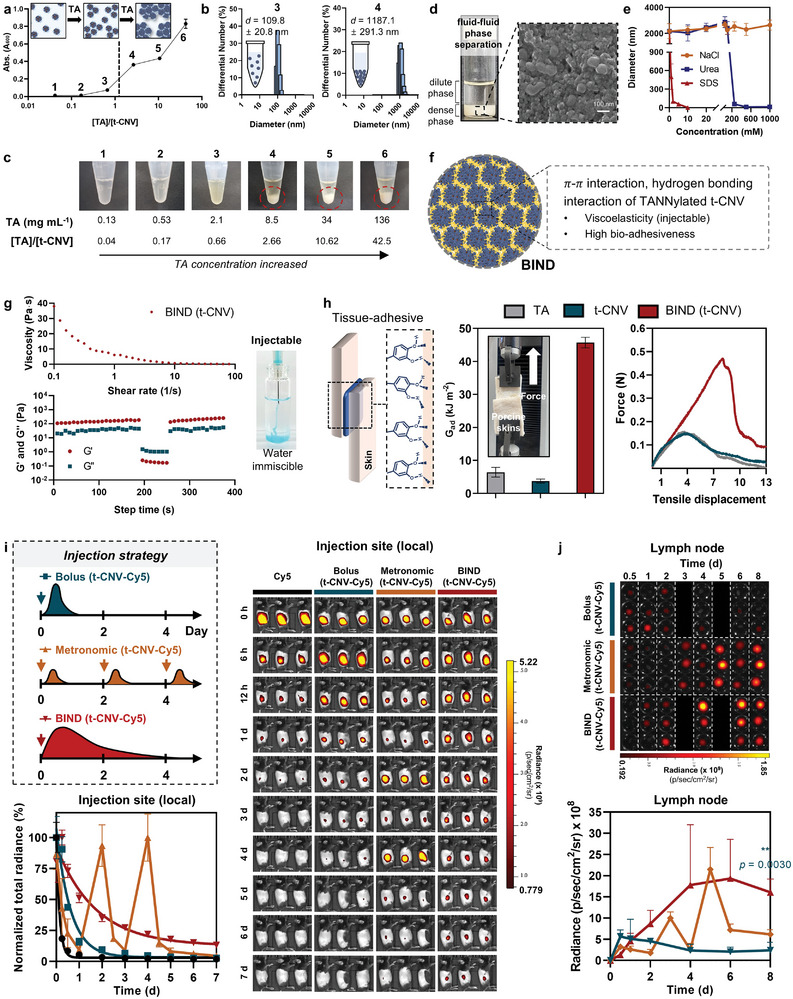
Physiochemical characterization of BIND for dynamic immune modulation at the macroscopic level. a) t‐CNV condensation depends on the concentration of tannic acid (TA). Turbidity measurements at A600 depend on the stoichiometric ratio of [TA]/[t‐CNV]. The dashed line represents the starting point of condensate formation (n = 3). b) Particle size distribution of points 3 and 4 in Figure 3a. c) Image of how condensate formation depends on the stoichiometric ratio of [TA]/[t‐CNV]. The red dashed circle represents the coacervate. d) Mixing t‐CNV and TA results in fluid‒fluid phase separation, with the dense phase identified as BIND (t‐CNV). A scanning electron micrograph of BIND (t‐CNV) is shown in the right insert. e) Dominant mode of interaction during the formation of BIND (t‐CNV) (n = 3). f) Depiction of chemical interactions during the formation of BIND (t‐CNV) and its mechanical properties. g) Rheological properties of BIND (t‐CNV). Viscosity versus shear rate of BIND (t‐CNV) (upper). G′ and G″ for BIND (t‐CNV) with alternating shear strains of 0.2% and 500% strain at a frequency of 1 Hz (lower). h) Lap shear test to determine bioadhesiveness (adhesive energy, G_ad_) (n = 3). i) IVIS images and normalized average radiance of fluorescent signal at the injection site over time depicting local retention after subcutaneous administration of the indicated samples in wild‐type C57BL/6 mice (n = 3). j) IVIS image and average radiance of fluorescent signal in the TDLN over time depicting the sustained availability of BIND. The indicated samples were peritumorally administered to E.G7‐OVA tumor‐bearing C57BL/6 mice (n = 3). All the data are presented as the mean ± SD. Statistical significance was evaluated by one‐way ANOVA with Tukey's multiple comparison test in h and by an unpaired two‐tailed *t* test in j. *P* values: NS, not significant; *P < 0.05, **P < 0.01, ***P < 0.001, ****P < 0.0001.

To spatiotemporally control the diffusivity of t‐CNV at both the injection site (peritumoral region) and targeted site (TDLN) over an extended period, we developed the BIND system in which t‐CNV is physically crosslinked by in situ with naturally occurring dendritic molecules of TA, which act as bioglue. We analyzed the biosafety of BIND (t‐CNV), and observed comparatively lower serum IL‐6 level and similar ALT and AST activity in the BIND (t‐CNV)‐treated group compared to the soluble OVA and R848 mixed group (Figure S14a,c, Supporting Information). Additionally, there were no significant body weight changes within 7 days post‐injection (Figure S14b, Supporting Information), indicating good biocompatibility and safety of BIND in vivo. TA, noted by the FDA as generally recognized as safe (GRAS) and widely used for animal studies,^[^ ^34^, ^35^, ^36^, ^37^, ^38^, ^39^ ^]^ contains multiple phenolic hydroxyl‐rich groups that can form hydrogen bonds and hydrophobic interactions with proteins,^[^ ^40^, ^41^ ^]^ including proline‐rich proteins, such as collagen^[^ ^42^ ^]^ and mucin.^[^ ^43^ ^]^ We systematically varied the relative stoichiometric ratios of TA and t‐CNV ([TA]/[t‐CNV]) within the range of 0.04–42.5 by mixing the two components in a 1:1 volume ratio while maintaining a constant t‐CNV concentration of 3.2 mg mL^−1^ (**Figure** **3**a‐c). As the TA concentration increased, it began to adhere to the surface of t‐CNV. When the [TA]/[t‐CNV] ratio exceeded the critical value of 2.66 (indicated by a dashed line), fluid–fluid phase separation occurred, resulting in the formation of a visible coacervate (Figure 3c). Conversely, when the [TA]/[t‐CNV] ratio remained below the critical threshold, specifically at 0.66 (indicated as 3), the A_600_ did not exhibit a notable increase and the nanoparticle size remained close to 100 nm, showing that coacervation did not occur under these conditions and maintained as TANNylated t‐CNV (Figure 3a‐c; Figure S15, Supporting Information). Based on the hypothesis that the distinct coacervation phase in the presence of the aqueous phase could create a prolonged depot effect after the injection of immunomodulatory drugs into the body, we defined this phase as the BIND. SEM and atomic force microscopy (AFM) images of the BIND revealed that t‐CNV maintained its nanoparticulate structure even after coacervation (Figure 3d; Figures S16 and S17, Supporting Information). This coacervation process is assumed to be driven primarily by hydrophobic interactions (π‐π interactions) and hydrogen bonding between TANNylated t‐CNV, as coacervate formation gradually disassembled with increasing concentrations of SDS and urea (Figure 3e,f). After the gel formation, the oxidation and degradation of tannic acid occur over time. TANNylated t‐CNV are expected to be released gradually from the gel's boundary in a sustained manner, facilitating delivery to LN (Figure S18a, Supporting Information). We verified this release ex vivo by incubating BIND (t‐CNV) in PBS buffer for 1 day at 37 °C (Figure S18b, Supporting Information). After the incubation, we observed a reduction in the amount of BIND (t‐CNV) within the gel, while particulate forms were detected in the supernatant by SEM.

The BIND system displayed high viscosity (≈40 Pa⋅s) and demonstrated shear‐thinning behavior, enabling BIND to be injectable as a viscous liquid (with G″ > G′) under stress. However, upon stress removal, BIND reverted to a gel‐like state (with G′ > G″) and formed a depot at the injection site (Figure 3g; Video S1, Supporting Information). Furthermore, BIND exhibited robust bioadhesiveness, with an adhesive energy (G_ad_) tenfold greater than that of t‐CNV and eightfold greater than that of TA. Additionally, the peak adhesion force of BIND occurred at a much larger deformation (Figure 3h). Due to the distinct mechanical properties of BIND, we investigated the kinetics of local retention and delivery into the surrounding or target tissue. To investigate the delivery kinetics of t‐CNV‐Cy5 from BIND (t‐CNV‐Cy5), we monitored the persistence of t‐CNV‐Cy5 following subcutaneous injection using in vivo fluorescence imaging (Figure 3i; Figures S19 and S20, Supporting Information). Although nearly all t‐CNV‐Cy5 had cleared from the injection site within 24 h post‐injection, BIND (t‐CNV‐Cy5) exhibited remarkable longevity, remaining at the injection site for more than three days. When t‐CNV‐Cy5 was administered in a metronomic manner (t‐CNV‐Cy5 (metronomic), where the total dose of the bolus was divided into three discrete injections with a 2‐day interval between each injection), the fluorescence signal showed high local intensity immediately after injection, significantly diminishing just 12 h after administration. These observations highlight the sustained and prolonged local retention provided by BIND compared to the rapid decline in retention exhibited by t‐CNV and t‐CNV (metronomic).

We compared the delivery dynamics in the TDLN after peritumoral injection of BIND (Figure 3j). In contrast to the bolus injection group, which showed a substantial decrease in the signal within the initial 12 h post‐injection, the metronomic injection group exhibited robust signaling at days 1, 3, and 5 following injections, even though these signals also notably diminished after one day. Notably, the metronomic injection approach, despite its rapid decrease in signal, played a pivotal role in generating both innate and adaptive immunity by stimulating the immune system for an extended period, as observed by analysis of the longitudinal population of innate immune cells and antigen‐specific CD8^+^ T cells within the TDLN in the B16‐OVA tumor model (Figures S21–S23, Supporting Information). Compared to metronomic delivery of t‐CNV, t‐CNV incorporated into the BIND system exhibited sustained and continuous delivery, and its accumulation in the TDLN increased for 7–8 days (Figure 3j). The metronomic‐like delivery kinetics observed in the TDLN for BIND (t‐CNV), exhibiting a continuous increase from day 1 to day 8, resembled the pattern observed during acute pathogenic infections, which are characterized by self‐replication for 7 days (Figures 3j and 1e).^[^ ^26^ ^]^

### 2.3 Metronomic‐Like Immune Modulation in the TDLN by BIND Evokes High Penetration Through the Paracortex and Durable Antigen‐Specific CD8^+^ T‐cell Responses

**Figure 4:**
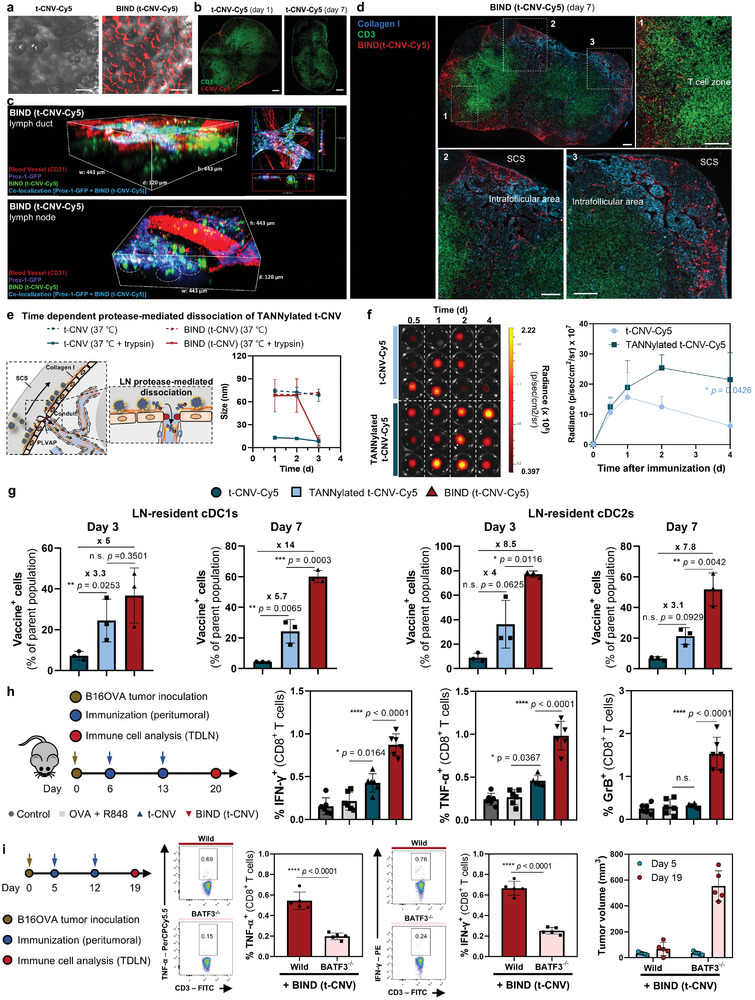
Sustained vaccine availability in the TDLN with high penetration through the paracortex, leading to the generation of durable antigen‐specific CD8^+^ T cells using BIND. a) Characterization of collagen binding affinity through representative confocal microscopy images of SpongeCol (a type I collagen sponge with columnar pore architecture) after incubation with t‐CNV‐Cy5 or BIND (t‐CNV‐Cy5) for 1 h. Scale bar, 500 µm. b) Representative confocal microscopy images of the intra‐LN distribution of t‐CNV‐Cy5 after peritumoral injection. Scale bar, 200 µm. c) Representative intravital image depicting the delivery of BIND (t‐CNV‐Cy5) to the LN: (upper) within the lymph duct at 3 days after peritumoral injection and (lower) within the LN 7 days after peritumoral injection. d) Representative confocal microscopy images depicting the in vivo intra‐LN delivery of BIND (t‐CNV‐Cy5) toward the paracortex through the conduit (collagen I‐stained) 7 days after peritumoral injection. Scale bar, 200 µm. e) Time dependent protease‐mediated dissociation of TANNylated t‐CNV in the subcapsular sinus (SCS). (left) Schematic illustration of the LN protease‐mediated dissociated of TANNylated t‐CNV in the SCS. (right) DLS analysis of time dependent t‐CNV or BIND (t‐CNV) after incubation at 37 °C in aqueous buffer with or without trypsin. f) IVIS image and average radiance of fluorescent signal in the TDLN over time depicting TDLN retention of TANNylated t‐CNV. The indicated samples were peritumorally administered to E.G7‐OVA tumor‐bearing C57BL/6 mice (n = 3). g) In vivo percentage of vaccine^+^ antigen‐presenting cells (APCs) in the TDLN of B16‐OVA tumor‐bearing mice 3 or 7 days after peritumoral injection of the indicated fluorescently labelled samples. APCs were defined as LN resident cDC1s (CD11b^−^CD11c^+^CD8^+^) and LN resident cDC2s (CD11b^+^CD11c^+^CD8^−^CD4^+^CD103^−^) (n = 3). h) Ag‐specific CD8^+^ T cells in the TDLNs of peritumorally immunized B16‐OVA tumor‐bearing mice treated with the indicated compounds (OVA, 22 nmol; R848, Trojan TLR7/8a, 16 nmol). Representative flow cytometry dot plots and percentages of IFN‐*γ* ^+^, TNF‐*α* ^+^ and GrB^+^ CD8^+^ T cells (CD3^+^CD8^+^) after SIINFEKL peptide restimulation and incubation with GolgiPlug for 6 h (n = 5–6). i) BATF3‐dependent antitumor immunity induced by BIND (t‐CNV). The population of Ag‐specific CD8^+^ T cells drastically decreased in BATF3^−/−^ mice following BIND (t‐CNV) immunization (n = 5). All data are presented as the mean ± SD. Statistical significance was evaluated by one‐way ANOVA with Tukey's multiple comparison test in g and h and by an unpaired two‐tailed *t* test in f and i. *P* values: NS, not significant; *P < 0.05, **P < 0.01, ***P < 0.001, ****P < 0.0001.

The fluorescence signal of BIND (t‐CNV‐Cy5) at the injection site diminished within 4 days, but the signal in the TDLN persisted for more than 6–8 days (Figure 3i,j). Therefore, we postulated that the longitudinal retention and accumulation of BIND (t‐CNV) in the TDLN after the supply was halted from the injection site could be facilitated by its binding affinity for structures in the LN. The LN is composed of fibrillar collagenous material, which is situated near the sinus lymphatic endothelial cells (LECs) and within the conduit, which is a transport channel traversing the sinus to the paracortex.^[^ ^13^, ^44^, ^45^, ^46^ ^]^ Since TA participates in multiple interactions with proline‐rich proteins such as collagen, we assessed the ability of BIND (t‐CNV) to bind to collagen. When t‐CNV‐Cy5 or BIND (t‐CNV‐Cy5) was incubated on purified type I collagen sponge discs (SpongeCol, pore size 100–400 µm), BIND (t‐CNV‐Cy5) became strongly attached, but no signal from t‐CNV‐Cy5 was detected in the collagen sponge disc (**Figure** **4**a). Subsequently, we evaluated the distribution of t‐CNV‐Cy5 within the TDLN structures by examining cryo‐section images of the TDLN after peritumoral injection (Figure 4b). t‐CNV‐Cy5 was predominantly located in the SCS on day 1, indicating limited penetration into the intra‐LN parenchyma, and t‐CNV‐Cy5 was no longer detectable on day 7, suggesting its rapid clearance from the TDLN. In the case of BIND (t‐CNV‐Cy5), a substantial amount was observed to track along the collagenous lymph duct and distribute throughout the TDLN, extending beyond the lymphatic epithelial cell layer of the SCS (Figure 4c; Videos S2 and S3, Supporting Information). In more detail, BIND (t‐CNV‐Cy5) closely followed collagen I in the SCS, tracking along the LN in alignment with the distribution of collagen I, being observed within the interfollicular area, paracortex and even deep into the T‐cell zone (Figure 4d). To elucidate the effect of TA on modulating the mode of action of t‐CNV, we conducted a comparative analysis of TDLN retention following injection of t‐CNV and TANNylated t‐CNV (Figure 4f). TANNylated t‐CNV demonstrated extended TDLN retention for more than 4 days, whereas the amount of t‐CNV diminished within 1 day within the TDLN (Figure 4f). This prolonged retention of TANNylated t‐CNV shows that it binds to collagen in the LN, enabling prolonged accumulation.

To elucidate how the high accumulation and retention of TANNylated t‐CNV in the SCS facilitates efficient delivery to the paracortex region, we examined the impact of prolonged exposure to temperature, protease (trypsin), or surfactant (SDS) on the size of BIND (t‐CNV) (Figure 4e; Figure S24a‐e, Supporting Information).^[^ ^47^, ^48^ ^]^ Exposure to body temperature led to the dissociation of BIND (t‐CNV) to generate TANNylated t‐CNV (≈100 nm) without further size reduction. However, incubation with trypsin for 3 days and SDS for 2 days decreased the size of BIND (t‐CNV) to ≈10 nm, indicating that the self‐assembled TANNylated t‐CNV dissociated into small‐sized TANNylated protein–adjuvant conjugates. Interestingly, TANNylated t‐CNV exhibited resistance to proteolysis compared to t‐CNV, which supported the prolonged retention of TANNylated t‐CNV in SCS (Figure 4e; Figure S24d,e, Supporting Information). We additionally verified that even after dissociation, TANNylated t‐CNV and the TANNylated protein–adjuvant conjugates retained their ability to bind collagen (Figure S24f,g, Supporting Information). Leveraging their small size and targeted collagen binding properties, TANNylated protein–adjuvant conjugates can penetrate the paracortex through the LN conduit. Importantly, the LN resident DCs lie on the surface of the conduit.^[^ ^44^ ^]^ Therefore, TANNylated protein–adjuvant conjugates that dissociate from BIND (t‐CNV) can be transported through the conduit directly to the paracortex and efficiently taken up by the LN resident DCs. We compared the percent uptake of t‐CNV‐Cy5, TANNylated t‐CNV‐Cy5, and BIND (t‐CNV‐Cy5) by LN resident cDC1s and cDC2s. Our findings demonstrated that the TANNylated and BIND system had a profound impact on cellular uptake, exhibiting 14‐fold and eightfold greater uptake of BIND (t‐CNV‐Cy5) than that of t‐CNV‐Cy5 by LN resident cDC1s and cDC2s, respectively, at 7 days after injection (Figure 4g; Figure S25, Supporting Information). The matured LN resident cDC1s and cDC2s induce adjacent T‐cell priming, leading to the generation of antigen‐specific T cells. Notably, BIND (t‐CNV) significantly enhanced the generation of antigen‐specific CD8^+^ T cells compared to t‐CNV (Figure 4h; Figures S22 and S26, Supporting Information). We confirmed that increased cellular uptake by LN resident cDC1s influenced the generation of antigen‐specific CD8^+^ T cells by comparing the responses of wild‐type and BATF3^−/−^ mice. In BATF3^−/−^ mice, the number of antigen‐specific CD8^+^ T cells generated by BIND (t‐CNV) was largely decreased, resulting in a significant decrease in the antitumor effect (Figure 4i). Additionally, we confirmed that TA in its soluble or coacervate form had no effect on the generation of antigen‐specific CD8^+^ T cells (Figure S27, Supporting Information).

### 2.4 Metronomic‐Like Immune Modulation in the TME Induces Prolonged IL‐12(p70) Secretion and Improved Antitumor Efficacy with Reduced T‐cell Exhaustion

**Figure 5:**
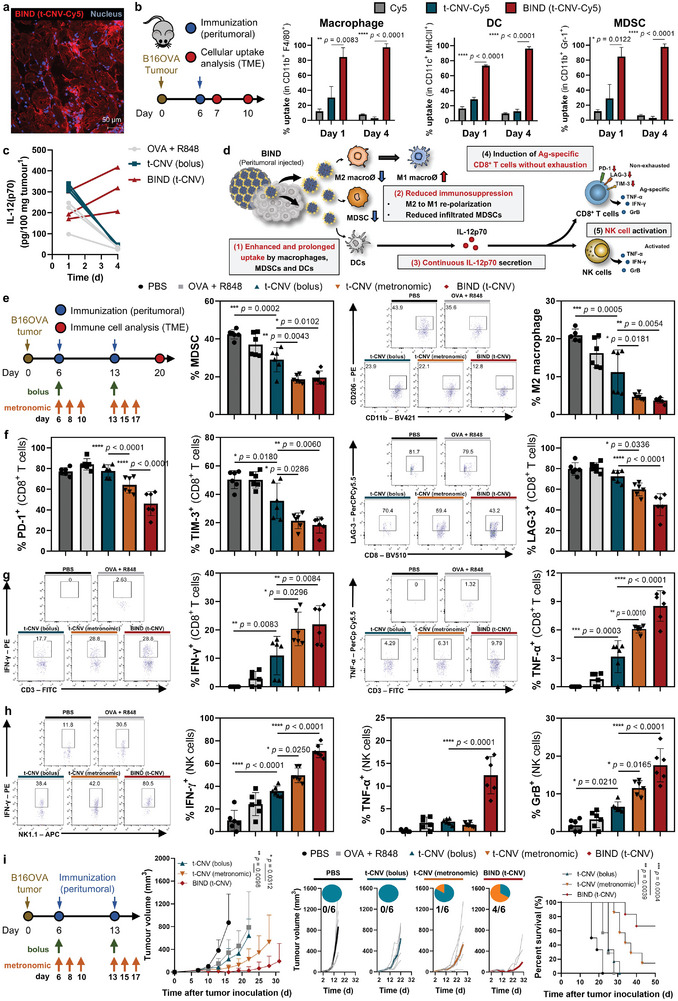
Reprogramming the dynamic immunological landscape of the TME by prolonged immune modulation by BIND. a) Representative confocal microscopy image of a frozen section from the retrieved BIND (t‐CNV‐Cy5) at the injection site one day after peritumoral injection illustrating cell infiltration. b) In vivo kinetics of the percent cellular uptake (vaccine^+^ cells) by macrophages (CD11b^+^F4/80^+^), DCs (CD11c^+^MHCII^+^), and MDSCs (CD11b^+^Gr‐1^+^) in the TME of B16‐OVA tumor‐bearing mice following peritumoral injection of the indicated fluorescently labelled samples (n = 3). c) In vivo kinetics of IL‐12(p70) secretion in the TME by B16‐OVA tumor‐bearing mice following peritumoral injection of the indicated compounds (OVA, 22 nmol; R848, Trojan TLR7/8a, 16 nmol) (n = 3). d) Schematic of the mode of action of BIND (t‐CNV) in the TME. e–h) Analysis of the immune cells in the TME of B16‐OVA tumor‐bearing mice following peritumoral injection of the indicated compounds (OVA, 22 nmol; R848, Trojan TLR7/8a, 16 nmol). Representative flow cytometry dot plots and percentages of MDSCs (CD11b^+^Gr‐1^+^ in CD45^+^) (n = 6) and M2 macrophages (CD11b^+^F4/80^+^CD206^+^ in CD45^+^) (n = 5–6) (e) and PD‐1^+^, TIM‐3^+^ and LAG‐3^+^ cells amongst the CD8^+^ T cells (n = 6) (f). Representative flow cytometry dot plots and percentages of Ag‐specific CD8^+^ T cells characterized by IFN‐*γ* ^+^ and TNF‐*α* ^+^ CD8^+^ T cells (CD3^+^CD8^+^) after SIINFEKL peptide restimulation and incubation with GolgiPlug for 6 h (n = 6) (g). Representative flow cytometry dot plots and percentages of IFN‐*γ* ^+^, TNF‐*α* ^+^ and GrB^+^ NK cells after incubation with a cell activation cocktail (with brefeldin A) or a mixture of the optimized concentrations of PMA, ionomycin and brefeldin A for 4 h (n = 6) (h). i) Antitumor efficacy in mice bearing subcutaneous B16‐OVA tumors following peritumoral administration (OVA, 22 nmol; Trojan TLR7/8a, 16 nmol) (n = 6–7). All data are presented as the mean ± SD. Statistical significance was evaluated by one‐way ANOVA with Tukey's multiple comparison test in e‐h and by an unpaired two‐tailed *t* test in b and i. Survival *P* values were calculated by the log‐rank (Mantel‒Cox) test in i. *P* values: NS, not significant; *P < 0.05, **P < 0.01, ***P < 0.001, ****P < 0.0001.

Following peritumoral injection, BIND (t‐CNV) formed a porous scaffold‐like structure in situ, allowing the infiltration of visible cells into the TME (**Figure** **5**a). We choose peritumoral injection over other routes, such as subcutaneous administration, because we aimed to deliver BIND (t‐CNV) directly to both TDLN and the TME. This choice was made because TLR7/8a are of interest not only for their adjuvant properties, which stimulate the innate immune system and generating antigen‐specific T‐cell responses, but also for their immunomodulatory functions that are crucial for reprogramming the immunosuppressive TME.^[^ ^7^, ^49^, ^50^ ^]^ Subsequently, we assessed the cellular uptake of BIND (t‐CNV) by macrophages, DCs, and MDSCs in the TME (Figure 5b). Although t‐CNV‐Cy5 exhibited modest cellular uptake after just one day, BIND (t‐CNV‐Cy5) demonstrated sustained high uptake over four days. This results in the prolonged secretion of IL‐12(p70) within the TME after treatment with BIND for more than four days (Figure 5c). The extended and enhanced cellular uptake of t‐CNV within the BIND system would potentiate the activity of TLR7/8a in the TME (Figure 5d). Therefore, BIND (t‐CNV) potentiates the activity of TLR7/8a in the M2 macrophage and MDSCs within the TME, significantly reducing the MDSC and M2 macrophage populations in the TME (Figure 5d,e; Figures S28 and S29, Supporting Information). Additionally, due to sustained IL‐12(p70) production by the BIND system, the BIND (t‐CNV)‐injected group showed reduced expression of exhaustion markers, including PD‐1, LAG‐3, and TIM‐3, on CD8^+^ T cells while the population of antigen‐specific CD8^+^ T cells was enhanced (Figure 5d,f,g, Figures S30–S32, Supporting Information). Moreover, the BIND (t‐CNV)‐injected group displayed NK cell activation, which was characterized by the elevated production of IFN‐*γ*, TNF‐*α*, and GrB (Figure 5h; Figures S33 and S34, Supporting Information). We assessed the antitumor efficacy of BIND (t‐CNV) in a B16‐OVA subcutaneous tumor model by monitoring tumor growth and survival rates (Figure 5i). Compared with bolus injection, metronomic administration of t‐CNV exhibited improved antitumor efficacy. However, BIND (t‐CNV) exhibited an even greater enhancement, resulting in 60% of the treated mice achieving a tumor‐free status.

### 2.5 Expandability of BIND to Various Immune Therapeutic Models

**Figure 6:**
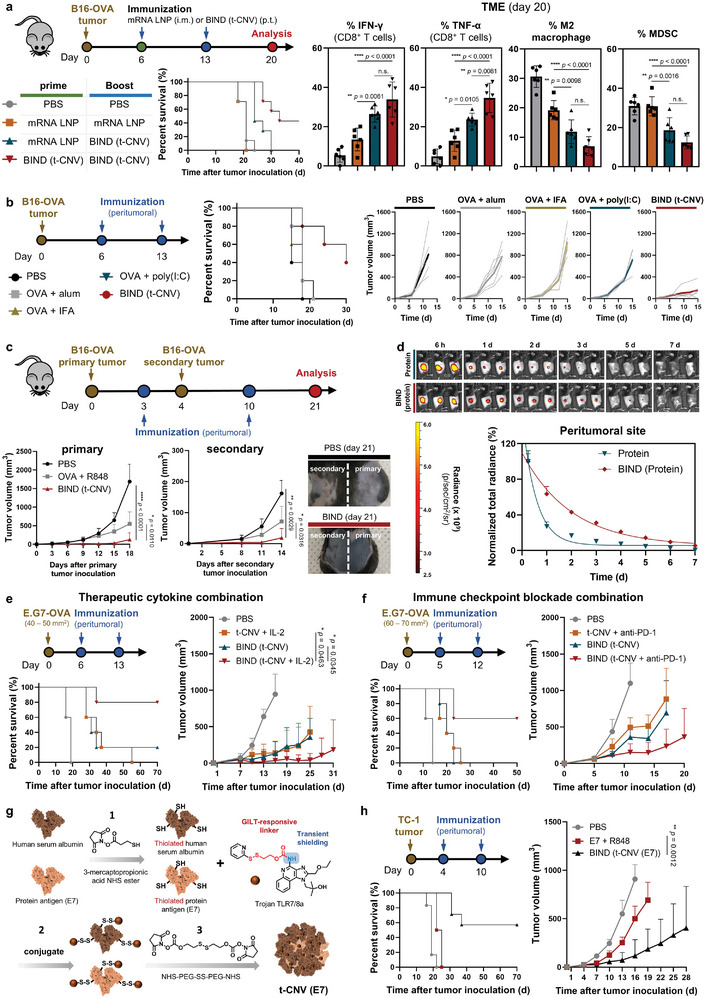
Expandability of BIND to various immune therapeutic models. a) Comparison of the antitumor efficacy of the mRNA LNP vaccine in a B16‐OVA melanoma tumor model: (left) percent survival rate following intramuscular (mRNA LNP) or peritumoral (BIND (t‐CNV)) immunization (OVA, 22 nmol; Trojan TLR7/8a, 16 nmol; CleanCap OVA mRNA, 5 µg) (n = 7); and (right) population of antigen‐specific CD8^+^ T cells and immunosuppressive cells (M2 macrophages and myeloid‐derived suppressor cells (MDSCs)) in the TME (n = 6). b) Comparison of the antitumor efficacy with the leading adjuvants in mice bearing B16‐OVA tumor‐bearing mice (OVA, 22 nmol; Trojan TLR7/8a, 16 nmol; alum, 140 µg; IFA, 10 µL; poly(I:C), 10 µg) (n = 5). c) Assessment of antitumor efficacy in primary and secondary tumor models: (left) primary and secondary tumor growth curves (n = 7); and (right) representative photographs of mice 21 days after primary tumor inoculation. d) IVIS images and normalized average radiance of fluorescent signal at the injection site over time depicting local retention after peritumoral protein (HSA; 66 kDa) administration in its soluble form or in the BIND formulation in wild‐type C57BL/6 mice (n = 3). e) The incorporation of protein‐based therapeutic cytokines into the BIND system enhances therapeutic efficacy: (left) percent survival; and (right) tumor growth curves following peritumoral immunization (OVA, 22 nmol; Trojan TLR7/8a, 16 nmol; IL‐2, 2 µg) (n = 5). f) Antibody‐based immune checkpoint blockade combined with the BIND system enhances therapeutic efficacy (OVA, 22 nmol; Trojan TLR7/8a, 16 nmol; anti‐PD‐1, 100 µg) (n = 5). g) Schematic of t‐CNV (E7) chemical synthesis. h) Antitumor efficacy of the BIND system with the E7 protein antigen in a TC‐1 tumor model: (left) percent survival; and (right) tumor growth curves following peritumoral immunization (HSA, 15.5 nmol; Trojan TLR7/8a, 30 nmol; E7 protein, 10 µg) (n = 6). All data are presented as the mean ± SD. Statistical significance was evaluated by one‐way ANOVA with Tukey's multiple comparison test in a (right) and by an unpaired two‐tailed *t* test in c, e and h (right). *P* values: NS, not significant; *P < 0.05, **P < 0.01, ***P < 0.001, ****P < 0.0001.

Although mRNA vaccines have been extensively explored as therapeutic cancer vaccines in numerous clinical trials, they still exhibit limited efficacy in tumor models where the immunosuppressive microenvironment has been firmly established.^[^ ^51^, ^52^ ^]^ Therefore, we postulated that BIND (t‐CNV), which induces potent antitumor immunity by dynamic immune modulation of suppressive microenvironment, can be a perfect companion to mRNA vaccines. In the B16‐OVA melanoma tumor model, mRNA lipid nanoparticles (LNPs) generated some antigen‐specific CD8^+^ T cells that secreted IFN‐γ and TNF‐α but had a limited effect on reducing the populations of immune suppressive cells such as MDSCs and M2 macrophages, compared to BIND (t‐CNV) (**Figure** **6**a; Figures S35–S37, Supporting Information). Interestingly, LNP/BIND (t‐CNV) prime/booster injections enhanced antigen‐specific CD8^+^ T cells and reduced the population of immunosuppressive cells compared to LNP/LNP prime/booster injections. Next, we conducted a comparative analysis of the BIND (t‐CNV) system with other leading adjuvants (Figure 6b). Our benchmark adjuvants included depot‐forming adjuvants (alum and IFA) and poly(I:C) (a TLR3 agonist). The results indicated that BIND (t‐CNV) outperformed these leading adjuvants in terms of effectively controlling tumor growth. To investigate the potential of using local treatment with BIND (t‐CNV) to elicit a systemic antitumor immune response, we conducted an experiment with a distant tumor model (Figure 6c). Remarkably, mice immunized with BIND (t‐CNV) exhibited responses not only in the local tumor but also effectively inhibited distant tumor growth, which suggested that localized treatment with BIND (t‐CNV) has the capacity to trigger a systemic antitumor immune response.

The BIND system can offer a versatile platform for the incorporation of various protein‐based immunotherapeutics, such as immune checkpoint inhibitors and therapeutic cytokines, through simple mixing. When the model protein (human serum albumin (HSA), 66 kDa) was integrated into the BIND system, enhanced tumor retention was exhibited compared to its standalone counterparts (Figure 6d). Such localization within the TME maximizes protein availability while preventing systemic distribution, potentially reducing immune‐related adverse events.^[^ ^53^, ^54^, ^55^ ^]^ Notably, we observed increased antitumor efficacy for the therapeutic cytokine IL‐2 and immune checkpoint blockade with anti‐PD‐1 antibody when these agents were incorporated into the BIND system (Figure 6e,f; Figure S38, Supporting Information). Finally, to expand the applicability of the BIND system to clinically relevant protein antigens, including those without free thiols on their structure, we propose a versatile strategy. We mixed HSA (building block) and the protein antigen at the desired ratio and chemically reacted them with 3‐mercaptopropionic acid NHS ester to introduce thiol groups to certain amines on the protein surface, facilitating easy conjugation with Trojan TLR7/8a (Figure 6g). Subsequently, we used the chemical linker NHS‐PEG‐SS‐PEG‐NHS to covalently link the Trojan TLR7/8a‐conjugated proteins, resulting in the formation of nanoparticulate structures. By testing this approach with the oncogenic viral protein HPV E7, a protein antigen for HPV‐associated cancers, we found that the antitumor efficacy of BIND (t‐CNV (E7)) was greater than that of a simple mixture of the HPV E7 protein and R848 (Figure 6h).

## 3 Conclusion

We suggest a designer biomaterial‐based metronomic‐like cancer immunotherapy by integrating the molecular design of t‐CNV, which is dynamically activated via the enzyme GILT at the cellular level, with the macroscopic acute infection‐like delivery kinetics provided by BIND at the tissue level. This strategy orchestrates multidimensional axes of immune responses, operating at both the cellular level (non‐tolerogenic DC activation, Th1 polarization, and T‐cell exhaustion mitigation) and the tissue level (prolonged and intra‐LN delivery, CD8^+^ T cell activation, and modulation of the immunosuppressive TME). t‐CNV induces non‐tolerogenic and activated immune cells through sustained TLR7/8 stimulation and enhances the cellular uptake of antigen–adjuvant conjugated formulations, enhancing their in vivo antitumor efficacy compared to that of nonconjugated formulations. The metronomic‐like delivery system based on BIND (t‐CNV) modulates the dynamic immunological landscape of the TME, reducing the populations of MDSCs and M2 macrophages and decreasing exhaustion markers (PD‐1, LAG‐3, and TIM‐3) on CD8^+^ T cells while enhancing the populations of activated CD8^+^ T cells and NK cells that secrete IFN‐*γ*, TNF‐*α*, and GrB. Notably, TANNylated t‐CNV dissociates from BIND (t‐CNV) and accumulates and is retained in the TDLN by specifically binding to collagen in the SCS and LN conduit, inducing its penetration into the paracortex through the LN conduit and facilitating uptake by the cDC1s that interact with CD8^+^ T cells in the T‐cell zone.

We have clarified that the primary mechanism underlying the intra‐LN delivery of TANNylated t‐CNV involves its disassembly into small‐sized TANNylated protein–adjuvant conjugate, a process that is driven by their prolonged retention and increased exposure to proteases in the SCS. Capitalizing on their small size and collagen binding properties, these TANNylated protein–adjuvant conjugates penetrate toward the paracortex through the collagen‐rich LN conduit. Notably, BIND (t‐CNV) can also be delivered to the LN by migratory DCs after phagocytosis, which is facilitated by the depot formed at the injection site (Figures S39 and S40, Supporting Information). However, it is generally understood that active targeting via migratory DCs represents a smaller portion than direct passive targeting to the LN.^[^ ^56^ ^]^

Despite the effectiveness of several engineering strategies, including the use of nanoparticles ≈100 nm in size, to passively target immunotherapeutic agents to the LN,^[^ ^1^, ^2^, ^3^, ^4^, ^57^, ^58^, ^59^ ^]^ nanoparticle penetration into the intra‐LN structure has been limited.^[^ ^12^, ^60^, ^61^ ^]^ This limitation arises because PLVAP (plasmalemma vesicle‐associated protein) forms a membrane structure in the conduit, acting as a sieve that allows the passage of low‐molecular‐weight proteins (< 70 kDa) only.^[^ ^13^, ^46^ ^]^ Shudel et al. proposed a two‐step delivery methodology wherein nanoparticles are rapidly conveyed to the LN upon peripheral tissue administration and subsequently traverse the intra‐LN node barriers by releasing small molecules via programable degradable linkers.^[^ ^62^ ^]^ However, our strategy for delivering TANNylated t‐CNV to the paracortex region via the LN conduit should be distinguished. TANNylated t‐CNV, with its inherent collagen binding ability, accumulates in the SCS for extended periods and gradually and continuously dissociates and traverses through the conduit, mimicking the self‐replication process of viruses during acute infection. Moreover, even after dissociation, TANNylated protein–adjuvant conjugates retain their collagen binding capacity, enabling their selective delivery through the collagenous structure of the LN conduit, a distinction from previous research.

Biomaterial‐based strategies to recruit and program innate immune cells at the administration site and sustain the release of immunotherapeutic agents for an extended period in the local environment, such as hydrogels, scaffolds, and microparticles, have been suggested.^[^ ^11^, ^15^, ^16^, ^17^, ^18^, ^63^ ^]^ However, from the point of view of LN delivery, the ability of these biomaterials to achieve metronomic‐like delivery of nanovaccines to the LN is an area that needs further exploration, as they often demonstrate only low signals for extended periods, which suggests the low accumulation of these nanovaccines in the LN (Figure S41, Supporting Information).^[^ ^18^ ^]^ This limitation arises because even though a low dose of antigens and adjuvants continuously reach the LN, there is limited LN adhesion and intra‐LN penetration, leading to their rapid flush away. The distinctive feature of the BIND system lies in metronomic‐like delivery of nanovaccine and its LN accumulation and retention, which are facilitated by its collagen binding affinity in the LN.

Our study should also be differentiated from previous studies that have reported robust germinal centre responses and higher antibody titres against HIV through osmotic pump‐enabled slow delivery immunization technology,^[^ ^64^ ^]^ in that we suggest an engineered biomaterial‐based metronomic‐like delivery technology that enables the generation of antigen‐specific effector CD8^+^ T cells and the reprogramming of the immunosuppressive TME, which are essential for the success of cancer immunotherapy.

We demonstrated the roles of TA in the development of BIND as follows (Figure S42, Supporting Information): 1) endowing t‐CNV with bioadhesive properties through hydrophobic and hydrogen interactions; 2) forming injectable gel‐like materials; 3) showing metronomic‐like delivery kinetics for TANNylated t‐CNV; 4) binding with collagen in the subcapsular sinus and LN conduit; and 5) enhancing resistance to proteolysis, promoting stability, and allowing prolonged attachment in the SCS.

In general, the interaction of TA and collagen is based on the hydrogen bonding and hydrophobic interaction. TA binds to the collagen with high affinity because the structural flexibility of the collagen compensates for the structural rigidity of the phenolics. However, the adhesion strength between tannylated t‐CNV in vivo condition would be completely different from that of in vitro and is expected to be complicated depending on the physiological state of LN (lymphatic flow and presence of various enzymes, etc.). The adhesion of TANNylated t‐CNV with collagen in LN would be decreased with time due to hydrolysis of TA, degradation by proteases (as shown in Figure S24, Supporting Information), and lymphatic flow, which enables the delivery into paracortex.

From a clinical translational perspective, our investigation suggests that BIND (t‐CNV) could be a perfect match for mRNA vaccines. Although mRNA vaccines have undergone extensive exploration as personalized therapeutic cancer vaccines in numerous clinical trials due to their versatility, their efficacy in tumor models is still limited due to challenges in controlling immunosuppressive cells within the TME. By dynamically modulating the immune landscape of the TME, BIND (t‐CNV) serves as an ideal candidate for administration as a booster after priming with an mRNA vaccine (Figure 6a). This approach not only diminished the population of immunosuppressive cells in the TME but also significantly increased the generation of antigen‐specific CD8^+^ T cells compared to administration of the mRNA vaccine. Moreover, in comparison with other clinically available adjuvants, including depot‐forming adjuvants (alum and IFA) and poly(I:C) (a TLR3 agonist), our novel adjuvant system, BIND (t‐CNV), showed outstanding antitumor efficacy (Figure 6b). Next, we demonstrated the versatile applicability of the BIND system to various FDA‐approved protein‐based immunotherapeutics, including the therapeutic cytokine IL‐2 and immune checkpoint blockade anti‐PD‐1 antibody, through simple mixing. After incorporating these immunomodulators within the BIND system, TANNylated IL‐2 or TANNylated anti‐PD‐1 antibody demonstrated time‐dependent release that was facilitated by binding to the collagen matrix in the TME. This ensures extended localization while potentially preventing systemic distribution and maximizing synergistic effects (Figure 6d).

To test the broad applicability of using this strategy as a booster treatment for enhanced humoral immunity, in addition to cellular immunity, both humoral and cellular immune responses induced by the BIND platform were studied. BIND (t‐CNV) exhibited continuous draining LN (dLN) delivery over 6–8 days following subcutaneous vaccination (Figure S43a, Supporting Information). Subsequent subcutaneous priming‐boosting vaccination of wild‐type mice with BIND (t‐CNV) effectively induced the generation of antigen‐specific CD4^+^ and CD8^+^ T cells, CD4^+^ Tfh cells, GC B cells and antigen‐specific IgG (Figures S43b‐j, S44–S46, Supporting Information). Remarkably, the cellular and humoral immunity elicited by BIND (t‐CNV) surpassed that induced by the depot‐forming alum system, which is commonly used as an adjuvant for infectious diseases (Figure S47, Supporting Information). Compared to conventional immunomodulation, in which functional immunomodulatory drugs are administered as a bolus and the response is autonomous, the dynamic immune modulation strategy revealed in this study provides a design principle for biomaterial‐based programmed immune regulation in cancer immunotherapy and can be applied to increase both cellular and humoral immune responses.

## 4 Experimental Section

### Synthesis and Characterization of Trojan TLR7/8a and t‐CNV

Trojan TLR7/8a was synthesized through the scheme shown in Figures S1–S4 (Supporting Information). The structures of the synthesized compounds were characterized by liquid chromatography–mass spectrometry (LC‒MS; Agilent 1260–6120 system) and high‐performance liquid chromatography (HPLC; Waters Acquity UPLC H‐Class instrument).

The timely‐activating conjugated nanovaccine (t‐CNV) was produced by the direct conjugation of Trojan TLR7/8a to a protein antigen. OVA (albumin from chicken egg white, Sigma–Aldrich) in PBS (10 mg mL^−1^) was reacted with various protein‐to‐drug‐ratio (OVA:Trojan TLR7/8a) in a 10 µL of tert‐butanol:ethanol mixture (3:1 volume ratio). The experimental condition for the protein‐to‐drug ratio as follows: **1:0** (protein 1 mg, PBS 100 µL, Trojan TLR7/8a 0 µg and organic solvent 10 µL), **1:1** (protein 1 mg, PBS 100 µL, Trojan TLR7/8a 12 µg and organic solvent 10 µL), **1:5** (protein 1 mg, PBS 100 µL, Trojan TLR7/8a 60 µg and organic solvent 10 µL) and **1:10** (protein 1 mg, PBS 100 µL, Trojan TLR7/8a 120 µg and organic solvent 10 µL). The conjugation reaction was performed at 4 °C for 1 h. The resulting immunoconjugates were purified by desalting (Zeba spin desalting columns, Thermo Fisher) twice to remove impurities with small molecular weights.

The amount of loaded Trojan TLR7/8a was quantified by ultraviolet‒visible light spectrometry (UV‐1800). The hydrodynamic size was measured using DLS (ELS‐Z electrophoretic light scattering photometer). The morphology was analyzed by field emission scattering electron microscopy (JSM‐7000F). The protein–drug conjugates were verified by LC‐Q/TOF‐MS (TripleTOF5600+). Fluorescent t‐CNV was synthesized and quantified according to the scheme shown in Figure S19 (Supporting Information).

### Endocytosis of t‐CNV

Immature BMDCs (4 × 10^4^ cells) were seeded in an ibidi U‐Slide 8‐well microscopy chamber. The cells were preincubated with dynasore (40 µm) for 1 h and incubated with samples at 37 °C for 4 h. After washing with PBS, the cell membrane was stained with wheat germ agglutinin Texas Red (Thermo Fisher), and the nuclei were stained with Hoechst 33342 (Invitrogen). Cell imaging was performed using a Leica TCS SP8 confocal laser scanning microscope (The BIORP of the Korea Basic Science Institute (KBSI)).

### Kinetics of t‐CNV Degradation After Endocytosis

t‐CNV (DQ‐OVA) was synthesized by incorporating DQ‐OVA (Thermo Fisher) into 1 out of every 10 OVA molecules. BMDCs were treated with t‐CNV (DQ‐OVA) (OVA, 0.2 µm) and incubated for specific durations. The cells were collected, and the mean fluorescence intensity (MFI) was measured by flow cytometry (BD FACSCanto II).

### GILT‐Dependent Cell Activation by t‐CNV

GILT‐dependent cell activation analysis was conducted as described previously.^[^ ^30^ ^]^ Briefly, Lipofectamine RNAiMAX (Thermo Fisher, 12 µL), IFI30‐specific siRNA or control siRNA (Genolotion), and Opti‐MEM (Thermo Fisher) were mixed and incubated at RT for 5 min. RAW 264.7 cells (2 × 10^5^ cells per well) were seeded in a 6‐well culture plate and treated with siRNA solution (40 pmol per well) to induce GILT gene knockdown. After 18 h of incubation, the cells were given fresh medium. t‐CNV was added to siGILT‐treated or siControl‐treated RAW 264.7 cells for 24 h, and the supernatants were collected to measure TNF‐*α* secretion.

### Animals, Cell Lines, and Antibodies

The animal study was reviewed and approved by the Institutional Animal Care and Use Committee (IACUC) of the Sungkyunkwan University School of Medicine (SKKUIACUC2020‐12‐13‐1), which was accredited by the Association for Assessment and Accreditation of Laboratory Animal Care International (AAALAC International) and abides by the Institute of Laboratory Animal Resources (ILAR) guidelines. C57BL/6 mice (6‐ to 8‐week‐old females) were purchased from Orient Bio (Korea). Female BATF3^−/−^ mice and female C57BL/6 OT‐1 mice were obtained from Prof. Yong‐Soo Bae (Sungkyunkwan University, Korea). Female Prox1‐GFP mice were obtained from IVIM Technology. All animals were housed in individually ventilated cages under 30–70% humidity at 21–26 °C on a 12 h light–dark cycle. To minimize the pain and distress caused by the tumor burden or related disease, it adhered to the IACUC guidelines, which state that “if the tumor volume exceeds 1 cm^3^ and causes significant impairment in the animal's behavior, the experiment must be terminated, and the animal should be humanely euthanized.” Accordingly, it established an endpoint criterion for the study, ensuring that the tumor volume to exceed 1000 (mm)^3^. All samples were injected into mice using the BD Ultra‐Fine II Insulin Syringe, 31 G. RAW 264.7 cells (ATCC) and B16‐OVA melanoma tumor cells (ATCC) were cultured in Dulbecco's modified Eagle's medium (DMEM, Thermo Fisher). E.G7‐OVA lymphoma tumor cells (ATCC) were cultured in RPMI 1640 medium (Thermo Fisher). The cell lines used in this study were used after confirming that they were free of mycoplasma contamination and were not listed amongst the misidentified cell lines. All media were supplemented with 10% heat‐inactivated foetal bovine serum (FBS; Thermo Fisher), penicillin (50 IU mL^−1^), and streptomycin (50 µg mL^−1^, Thermo Fisher). Detailed information on the antibodies used in this study, such as the fluorescent antibody type, manufacturer, clone, and catalogue number, is provided in Tables S2 and S3 (Supporting Information).

### Synthesis and Characterization of the Bioadhesive Immune Niche Domain (BIND)

t‐CNV (3.2 mg mL^−1^, PBS) and tannic acid (TA) (8.5 mg mL^−1^, PBS) were mixed vigorously at a volume ratio of 1:1 to achieve a stoichiometric ratio of [TA]/[t‐CNV] of 2.66. The mixture was incubated at RT for 30 min to induce the formation of coacervates. Subsequently, the mixture was subjected to centrifugation at 1500 × g for 3 min, which was repeated twice to effectively remove any excess TA.

Fluorescent BIND (t‐CNV) was prepared in a similar manner, except that fluorescently labelled t‐CNV was used instead of t‐CNV.

### Turbidity Assay

Solution turbidity was measured by UV–vis spectroscopy by measuring the absorbance 600 nm. The hydrodynamic size was measured using DLS. The morphology was analyzed by field emission scattering electron microscopy.

### Rheological Characterization

The rheological characterization of BIND was evaluated by using an ARES‐G2 rheometer (TA Instruments). All measurements were performed at 25 °C using a parallel steal plate geometry (25 mm in diameter). First, the viscosity was evaluated with increasing shear force by the stepped flow test (0.1–100 s^−1^, 1 Hz frequency). Next, the resilience and fluid strength after strong strain were measured. The storage modulus (G′) and loss modulus (G″) were measured by oscillatory time sweep experiments for 3 min (0.2% strain, 1 Hz frequency) before strong strain (1 min, 500% strain, 1 Hz frequency). Then, the oscillatory time in weep mode was restored (2 min, 0.2% strain, 1 Hz frequency).

### Bioadhesion Test

Fresh porcine skins were stored on ice and washed with PBS to remove fat before use. A total of 150 µL of each sample was applied at the interface of two porcine skins, and a shear stress–strain curve was constructed after using a universal testing machine (UTM, Instron 5943). The adhesion energy (G_ad_) of each sample was calculated using the following equation: (*G_ad_* =  3(*F*/*w*)^2^/(2*Eh*)) where *F* represents the measured adhesive tensile stress at the maximum force. *w* and *h* denote the width and height of the pork skin, respectively (*w*  =  30 mm and *h*  =  20 mm), and *E* represents the tensile modulus of pork skin. The values for *F* and *E* of each group are provided in Table S4 (Supporting Information).

### Preparation of Single‐Cell Suspensions

Tumors or inguinal LNs were mechanically disrupted and resuspended in medium containing collagenase D (1 mg mL^−1^, Sigma–Aldrich). The solutions were incubated in a shaking incubator for 40 min at 37 °C. Then, the cells were washed twice with PBS after filtration through 70‐µm cell strainers.

### CD4^+^ Tfh Cell, GC B Cell, MDSC, M2 Macrophage, and Exhausted CD8^+^ T Cell Populations

Single cells were stained with BD Horizon Fixable Viability Stain 780, as well as surface marker antibodies specific for CD4^+^ Tfh cells (anti‐mouse CD4, PD‐1, and CXCR5), GC B cells (anti‐mouse B220, IgD, GL‐7, and FAS), MDSCs (anti‐mouse CD45, CD11b, and Gr‐1), M2 macrophages (anti‐mouse CD45, CD11b, F4/80, and CD206), and exhausted CD8^+^ T cells (anti‐mouse CD3, CD8, PD‐1, LAG‐3, and TIM‐3). Detailed information on the antibodies used is provided in Table S3 (Supporting Information), and the gating strategies used are provided in Figures S28, S30, S36 and S47 (Supporting Information).

### Antigen Specificity of CD4^+^ and CD8^+^ T Cells and Activation of NK Cells

For antigen‐specific CD8^+^ T‐cell analysis, single cells (5 × 10^5^ per well) were seeded in a round‐bottom 96‐well plate. Then, the cells were restimulated with the OVA peptide SIINFEKL (10 µg mL^−1^) and GolgiPlug (a protein transport inhibitor, 0.6 µg mL^−1^, BD Bioscience) for 6 h. For NK cell stimulation analysis, single cells (5 × 10^5^ per well) were seeded in a round‐bottom 96‐well plate. The cells were stimulated with a cell activation cocktail (with brefeldin A) (BioLegend, 500×), which was a mixture of optimized concentrations of PMA, ionomycin, and the protein transport inhibitor brefeldin A for 4 h. After stimulation, the cells were collected and washed twice. The cells were then stained with BD Horizon Fixable Viability Stain 780, surface marker antibodies specific for CD4^+^ T cells (anti‐mouse CD3 and CD4), CD8^+^ T cells (anti‐mouse CD3 and CD8) or NK cells (anti‐mouse CD3 and NK1.1) for 30 min at 4 °C. Detailed information on the antibodies used is provided in Table S3 (Supporting Information). Subsequently, for intracellular staining, the cells were washed and resuspended in fixation/permeabilization solution for 20 min at 4 °C. Fixed cells were washed twice with BD Perm/Wash buffer (BD Bioscience) and stained with antibodies specific for cytokine‐producing CD4^+^ or CD8^+^ T cells (anti‐mouse IFN‐*γ*, TNF‐*α*, and granzyme B) or cytokine‐producing NK cells (anti‐mouse IFN‐*γ*, TNF‐*α*, and granzyme B) for 30 min at 4 °C. After staining, the cells were washed twice with BD Perm/Wash buffer and resuspended in staining buffer. Flow cytometry data were analysed using a BD FACSCanto II (The BIORP of the Korea Basic Science Institute (KBSI) and quantified using FlowJo V 10. Detailed information on the antibodies used is provided in Table S3 (Supporting Information), and the gating strategies used are provided in Figures S22,S26, S32, S34, S37, S44, and S45 (Supporting Information).

### Statistical Analysis and Reproducibility

All the results were presented as the mean ± standard deviation (SD.). Two‐tailed unpaired *t* tests were used to compare two groups. One‐way ANOVA (or two‐way ANOVA) with Tukey's multiple comparisons test (or Sidak's multiple comparisons test) was used to analyze multiple groups of data. The log‐rank (Mantel–Cox) test was used for survival data. All the statistical analyses were performed using GraphPad Prism 8 and Microsoft Excel 2016. *P* values (NS: not significant; *P < 0.05, **P < 0.01, ***P < 0.001, and ****P < 0.0001) were used to indicate statistical significance. The experiments shown in Figures 2, 3, 4a,b,d and 5a were repeated three times on distinct samples. The experiments shown in Figures 2i and 3c were repeated two times with distinct samples.

## Conflict of Interest

The authors declare no conflict of interest.

## Supporting information



Supporting Information

Supplemental Video 1

Supplemental Video 2

Supplemental Video 3

## Data Availability

The data that support the findings of this study are available from the corresponding author upon reasonable request.
