# Supplementary material for: Transformable Gel‐to‐Nanovaccine Enhances Cancer Immunotherapy via Metronomic‐Like Immunomodulation and Collagen‐Mediated Paracortex Delivery
Source: Adv Mater. 2024 Oct 9;36(48):2409914. doi: 10.1002/adma.202409914 (PMC11602686; doi:10.1002/adma.202409914)
Supplement: Supplementary file 1 — Supporting Information [file ADMA-36-2409914-s004.pdf]

ADVANCED MATERIALS

Supporting Information

for *Adv. Mater.*, DOI 10.1002/adma.202409914

Transformable Gel-to-Nanovaccine Enhances Cancer Immunotherapy via Metronomic-Like Immunomodulation and Collagen-Mediated Paracortex Delivery

*Seung Mo Jin, Ju Hee Cho, Yejin Gwak, Sei Hyun Park, Kyungmin Choi, Jin-Ho Choi, Hong Sik Shin, JungHyub Hong, Yong-Soo Bae, Jaewon Ju, Mikyung Shin and Yong Taik Lim**

Supporting Information

Transformable Gel-to-Nanovaccine Enhances Cancer Immunotherapy via Metronomic-like Immunomodulation and Collagen-mediated Paracortex Delivery

Seung Mo Jin, Ju Hee Cho, Yejin Gwak, Sei Hyun Park, Kyungmin Choi, Jin-Ho Choi, Hong Sik Shin, JungHyub Hong, Yong-Soo Bae, Jaewon Ju, Mikyung Shin and Yong Taik Lim[*]

Experimental procedures

***In vitro* bone marrow-derived dendritic cell (BMDC) culture.**

BMDCs were prepared as described previously[30]. Briefly, the bone marrow of C57BL/6 mice (6- to 8-week-old females) was collected and plated in 6-well culture plates with culture media containing 20 ng ml^{-1} mGM-CSF (CreaGene). On day 2, the cell culture medium containing mGM-CSF was refreshed after the cells were vigorously washed with PBS to remove the nonadherent cells. On day 4, fresh medium containing mGM-CSF (20 ng ml^{-1}) was added. After 6 days of differentiation, the BMDCs were collected for use.

In vitro cytokine assay. After 6 days of differentiation, BMDCs were treated with the indicated samples and incubated for specific durations. To analyse the concentrations of cytokines such as IL-12(p70), IL-6, TNF-α, and IL-10 in the cell supernatants, an OptEIA ELISA kit (BD Science) was used according to the manufacturer's instructions.

***In vitro* Th1/Th2 polarization of naïve $CD4^+$ T cells.**

BMDCs were pretreated with OVA+R848 or t-CNV (OVA, 6.2 μM; R848, 4.5 μM) for 12 h. Spleens were harvested from C57BL/6 mice, and naïve $CD4^+$ T cells were isolated using a naïve $CD4^+$ T-cell isolation kit (Miltenyi Biotec) according to the manufacturer's protocol. Preincubated DCs were cocultured with $CD4^+$ T cells (DC:T-cell ratio = 1:10) in a 96-well flat bottom plate. After 5 and 7 days, the cell culture supernatants were collected, and IL-4 and IFN-γ secretion was determined by ELISA.

***In vitro* exhaustion of OT-1 $CD8^+$ T cells.**

Exhausted OT-1 $CD8^+$ T cells were prepared as described previously[30]. Briefly, spleens from 6- to 8-week-old OT-1 transgenic mice (C57BL/6, female) were harvested, and $CD8^+$ T cells were isolated using a mouse $CD8^+$ T-cell isolation kit (Miltenyi Biotec). In a 24-well cell culture plate, 5×10^5 cells per well were cultured in RPMI 1640 medium (ATCC modification, Gibco) supplemented with 10% FBS, 2 mM L-glutamine, 10 mM HEPES, 1 mM sodium pyruvate, 100 U ml^{-1} penicillin, 100 μg ml^{-1} streptomycin sulfate and β-mercaptoethanol (0.05 mM, Sigma Aldrich). IL-2 (5 ng ml^{-1}, PeproTech) and the $OVA_{257-264}$ peptide SIINFEKL (10 ng ml^{-1}, MIMOTOPES) were added with daily addition of SIINFEKL peptide and IL-2 every two days for five days. Six days later, BMDCs (1×10^5 cells per well) and exhausted OT-1 $CD8^+$ T cells (5×10^5 cells per well) were cocultured in a 96-well cell culture plate, and the cells were treated with OVA+R848 or t-CNV (OVA, 6.2 μM; R848, 4.5 μM). After 24 h, the cells were collected and stained with antibodies specific for exhausted $CD8^+$ T cells (anti-mouse CD3, CD8, and PD-1), and their phenotypes were analysed via flow cytometry (BD FACSCanto™ II). Detailed information on the antibodies is provided in Table S3.

Comparison between the conjugated and mixed forms of the protein antigen and small molecule.

In the mixed group, OVA was reacted with AF488 NHS ester (Lumiprobe), and the excess dye was subsequently removed. The detailed dye-protein conjugation procedure and the quantification method were followed, as outlined in Figure S19. Subsequently, AF488-labelled OVA was combined with cyanine 5 amine (Lumiprobe). For the conjugated group, t-CNV was reacted with a mixture of sulfo-cyanine 5 NHS ester (Lumiprobe) and AF488 NHS ester (Lumiprobe) at a volume ratio of 1:1, followed by the removal of excess dye. The amounts of molecular Cy5 and AF488 in each group were quantified and adjusted to contain equimolar amounts of dye. To compare the effects *in vitro*, immature BMDCs were treated with both mixed and conjugated samples for 4 h. After treatment, the cells were washed with PBS, and the nuclei were stained with Hoechst 33342 (Invitrogen). Cell imaging was performed using a Leica TCS SP8 confocal laser scanning microscope. For *in vivo* comparisons, mixed and conjugated samples were peritumorally injected into C57BL/6 mice. After 12 h, the TDLNs were collected. Single-cell suspensions were prepared from the TDLNs and stained with BD Horizon Fixable Viability Stain 780, as well as antibodies specific for anti-mouse CD11b and CD11c. Detailed information about the antibodies used is provided in Table S3, and the gating strategy used is provided in Figure S12. The ratio of the double-positive area ($Cy5^+$ $AF488^+$) to the single-positive areas ($Cy5^-$ $AF488^+$ and $Cy5^+$ $AF488^-$) was calculated for analysis.

***In vivo* trafficking in mice and murine tissue.**

To assess the local retention and LN infiltration of the fluorescently labelled samples, whole mice or inguinal LNs harvested from mice were imaged with an IVIS Lumina XR (Perkin Elmer). Image analysis to determine the total radiant efficiency was performed using Living Image Software.

***In vivo* fluorescence imaging.**

To assess fluorescence at the injection site or in the LN, whole mice or LNs collected from animals were analysed via an optical imaging IVIS Lumina XR *in vivo* imaging system (Perkin Elmer).

Collagen binding analysis with SpongeCol®.

First, 20 µl of t-CNV-Cy5 or BIND (t-CNV-Cy5) was added to commercial SpongeCol® (Advanced Biomatrix) and incubated for 1 h at 37 °C. After incubation, the SpongeCol® was vigorously washed with PBS 10 times, and fluorescence images were obtained with a Leica TCS SP8 confocal laser scanning microscope.

Immunofluorescence imaging of the LNs.

The extracted LNs were immersed in 4% paraformaldehyde for 24 h and equilibrated in a 30% sucrose solution for 24 h, followed by tissue embedding using Tissue-Tek OCT compound (Sakura). After freezing, the LNs were sectioned using a Leica rotary microtome (RM2165). The sectioned tissues were washed and blocked with 5% FBS solution for 1 h. After washing with PBS, the sections were stained with antibodies overnight at 4 °C. Detailed information about the antibodies used can be found in Table S2. Fluorescence images were obtained with a Leica TCS SP8 confocal laser scanning microscope.

Intravital confocal microscopy imaging of the LNs.

A commercial intravital confocal microscopy (IVM-CM) system (IVIM Technology) was utilized for visualizing the dynamics of BIND (t-CNV) in the LN. For intravital imaging, BIND (t-CNV-Cy5) was injected into the right flanks of Prox-1-GFP transgenic mice. Three and seven days after injection, the mice were anaesthetized and placed on a motorized animal stage under an intravital microscope for imaging. A heating pad was applied under the mice, and the thermal probe was placed inside the rectum for continuous monitoring of body temperature. To identify the vasculature, 25 µg of CD31 antibody (553708; BD Biosciences, Franklin Lakes, USA) conjugated with the fluorescent probe FSD 555 (KOSC1003; BioActs, Korea Republic) was intravenously injected 2 h before intravital imaging. For motility analysis, time-lapse imaging of each cell was performed for 10 min.

***In vivo* cytokine analysis.**

OVA+R848, t-CNV, or BIND (t-CNV) (OVA, 22 nmol; R848, Trojan TLR7/8a, 16 nmol) was peritumorally injected into B16-OVA tumor-bearing mice (C57BL/6, 6-week-old females), and for cytokine secretion analysis in tumors, tumor tissues were collected at specific timepoints. The tumor tissues were suspended in CellLytic™ MT cell lysis reagent (100 mg tissue ml^{-1}, Sigma Aldrich) and mechanically disrupted. After incubating at 4 °C for 10 min, the tissue lysate was centrifuged at 10,000 × g for 10 min at 4 °C, and the supernatants were collected and analysed with an IL-12(p70) ELISA kit (BD Science) according to the manufacturer's protocol.

***In vivo* cellular uptake analysis.**

For the analysis of *in vivo* cellular uptake, fluorescent samples were peritumorally injected into B16-OVA tumor-bearing mice (C57BL/6, 6-week-old females). The TDLNs or tumor tissues were collected at specific timepoints, and single-cell suspensions were prepared as indicated above. To analyse cellular uptake in the TDLN, single cells were stained with BD Horizon Fixable Viability Stain 780, as well as surface marker antibodies specific for macrophages (anti-mouse CD11b, CD11c, F4/80, and Ly6G) and dendritic cells (anti-mouse CD11b, CD11c, CD4, CD8, and CD103). To analyse cellular uptake in the TME, single cells were stained with BD Horizon Fixable Viability Stain 780, as well as surface marker antibodies specific for macrophages (anti-mouse CD11b and F4/80), dendritic cells (anti-mouse CD11c and MHC II) and MDSCs (anti-mouse CD11b and Gr-1). Detailed information on the antibodies used is provided in Table S3, and the gating strategies used are provided in Figure S25, S39 and S40.

***In vivo* antibody titre analysis.**

Naïve C57BL/6 mice (6 weeks old, female) were vaccinated with the indicated samples, and blood was collected. Serum was isolated from the blood samples by centrifugation at 10,000 × g for 20 min. For the IgG ELISA, a 96-well plate was coated with recombinant OVA protein (2 µg ml^{-1}) and incubated overnight. The plate was then washed and blocked for 3 h at 37 ° C with 5% (w/v) skim milk. Serum samples were added to the plate and incubated for 2 h at 37 ° C. A secondary antibody for IgG, goat anti-mouse IgG (H+L)-HRP, was added to each well at a 1:6000 dilution. After 1 h of incubation, the plate was washed, and the samples were developed with a TMB solution. The absorbance at 450 nm was measured using a microplate reader (VersaMax).

***In vivo* antitumor efficacy analysis.**

B16-OVA tumor cells (5×10^5 cells per mouse) were subcutaneously inoculated into the right flanks of C57BL/6 mice (6-week-old females). Six days after tumor cell inoculation, OVA+R848, t-CNV either in a bolus or metronomic manner, or BIND (t-CNV) (OVA, 22 nmol; R848, Trojan TLR7/8a, 16 nmol) was administered according to the indicated schedule. The tumor-bearing mice were evenly assigned to their respective treatment groups based on tumor size, with the PBS-treated group serving as the control. Tumor growth and animal survival were monitored at various time points. Tumor volume was calculated using the following formula: (long axis diameter) $\times$ (short axis diameter)^2/2. Mice were euthanized when the tumor volume reached the maximum tumor size (1,000 mm^3) approved by the IACUC, Sungkyunkwan University School of Medicine.

Comparison of the therapeutic efficacy with that of the mRNA vaccine.

LNPs containing OVA mRNA were synthesized with a microfluidic device (NanoAssemblr® Ignite™, Precision Nanosystems). A mixture of 100 mg ml^{-1} ALC-0315 (MedChemExpress), 50 mg ml^{-1} ALC-0159 (MedChemExpress), 10 mg ml^{-1} 1,2-distearoyl-sn-glycero-3-phosphocholine (DSPC) (Avanti), and 10 mg ml^{-1} cholesterol (Sigma–Aldrich) was dissolved in ethanol (organic phase) at a weight ratio of 43/5/9/20, and 0.1 mg ml^{-1} CleanCap® OVA mRNA was dissolved in pH 4 buffer (100 mM sodium citrate, aqueous phase). The organic and aqueous phases were mixed at a 3:1 volume ratio using a microfluidic device. The synthesized LNPs were dialyzed overnight using Slide-A-Lyzer™ Dialysis Cassettes, 10K MWCO (Thermo Fisher) in 1$\times$ PBS.

B16-OVA tumor cells (5×10^5 cells per mouse) were subcutaneously inoculated into the right flanks of C57BL/6 mice (6-week-old females). Six days after tumor cell inoculation, LNPs (intramuscular) or BIND (t-CNV) (peritumoral) (OVA, 22 nmol; Trojan TLR7/8a, 16 nmol; mRNA, 5 μg) were administered according to the indicated schedule. Mouse tumor growth and survival rates were monitored at various time points. Twenty days after tumor inoculation, the immune cells in tumor tissues were analysed using flow cytometry (BD FACSCanto™ II).

Comparison of the therapeutic efficacy with that of conventional adjuvants.

B16-OVA tumor cells (5×10^5 cells per mouse) were subcutaneously inoculated into the right flanks of C57BL/6 mice (6-week-old females). Six days after tumor cell inoculation, OVA+ alum, OVA+IFA, OVA+poly(I:C) or BIND (t-CNV) (OVA, 22 nmol; Trojan TLR7/8a, 16 nmol; alum, 140 μg; IFA, 10 μl; poly(I:C), 10 μg) was administered according to the indicated schedule. Mouse tumor growth and survival rates were monitored at various time points.

In vivo distant tumor model. B16-OVA tumor cells (5×10^5 cells per mouse) were subcutaneously inoculated into the right flanks of C57BL/6 mice (6-week-old females) on day 0. Four days after primary tumor inoculation, secondary tumor cells (2.5×10^5 cells per mouse) were subcutaneously inoculated into the left flanks. OVA+R848 or BIND (t-CNV) (OVA, 22 nmol; R848, Trojan TLR7/8a, 16 nmol) was injected twice at a at 7-day interval 4 days after primary tumor inoculation. Mouse tumor growth was monitored at various time points.

Combination with a therapeutic cytokine. E.G7-OVA tumor cells (5×10^5 cells per mouse) were subcutaneously inoculated into the right flanks of C57BL/6 mice (6-week-old females) on day 0. Six days after tumor inoculation, t-CNV with IL-2, BIND (t-CNV), or BIND (t-CNV) with IL-2 (OVA, 22 nmol; Trojan TLR7/8a, 16 nmol; IL-2, 2 μg) was injected twice at a 7-day interval. Mouse tumor growth and the animal survival rates were monitored at various time points.

Combination with immune checkpoint blockade. E.G7-OVA tumor cells (5×10^5 cells per mouse) were subcutaneously inoculated into the right flanks of C57BL/6 mice (6-week-old females) on day 0. Five days after tumor inoculation, t-CNV with anti-PD-1, BIND (t-CNV), or BIND (t-CNV) with anti-PD-1 (OVA, 22 nmol; Trojan TLR7/8a, 16 nmol; anti-PD-1, 100 μg) was injected twice at a 7-day interval. Mouse tumor growth and the animal survival rates were monitored at various time points.

***In vivo* antitumor efficacy analysis after immunization with t-CNV (E7).**

Synthesis of t-CNV (E7). Human serum albumin (HSA) (Sigma–Aldrich) and recombinant HPV16 E7 protein (LSBIO) in PBS (10 mg ml^{-1}) were reacted with 15 equivalents of 3-mercaptopropionic acid NHS ester (MedChemExpress) in DMSO (20 mg ml^{-1}). The conjugation reaction was performed at RT for 1 h. The resulting product was purified by desalting (Zeba spin desalting columns, Thermo Fisher) twice to remove impurities with small molecular weights. Thiolated HSA and thiolated E7 were then reacted with 25 equivalents of Trojan TLR7/8a in DMSO (15 mg ml^{-1}) at 4 °C for 1 h. The resulting product was purified by desalting (Zeba spin desalting columns, Thermo Fisher) two times to remove impurities with small molecular weights. To synthesize the particulate formulation, Trojan TLR7/8a-conjugated HSA and Trojan TLR7/8a-conjugated E7 were dissolved in PBS at 2 mg ml^{-1}. Then, ethanol was added with vigorous stirring at a rate of 1 ml min^{-1} using a syringe pump, amounting to half the volume of the protein solution. NHS-PEG1-SS-PEG1-NHS (BroadPharm) was added for protein crosslinking, which proceeded over 30 min with stirring at RT. To remove the ethanol from t-CNV (E7), the solution was purified using 10,000 MWCO Amicon® ultracentrifugal filters (Merck) twice and redispersed in PBS.

In vivo antitumor efficacy. TC-1 tumor cells (5×10^5 cells per mouse) were subcutaneously inoculated into the right flanks of C57BL/6 mice (6-week-old females). Four days after tumor cell inoculation, the mice were treated with E7+R848 or BIND (t-CNV (E7)) (HSA, 15.5 nmol; Trojan TLR7/8a, 30 nmol; E7 protein, 10 μg) according to the indicated schedule. Mouse tumor growth and animal survival rates were monitored at various time points.

Synthesis of Trojan TLR7/8a

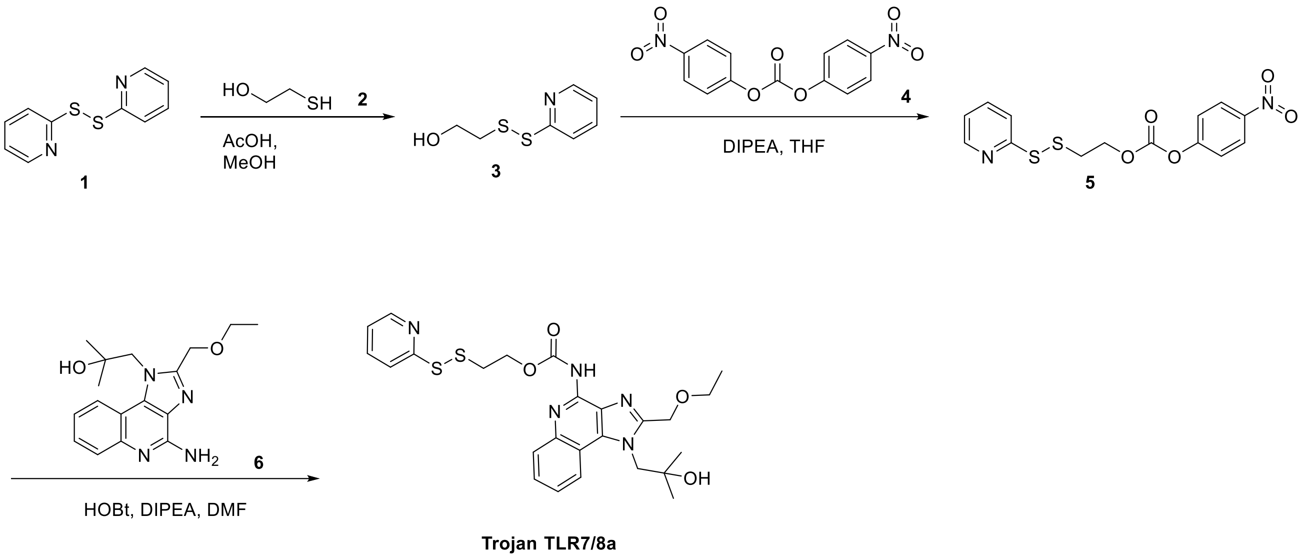

Figure S1. Schematic illustration of the synthesis of Trojan TLR7/8a (2-(pyridin-2-yl-disulfaneyl)ethyl (2-(ethoxymethyl)-1-(2-hydroxy-2-methylpropyl)-1H-imidazo[4,5-c]quinolin-4-yl)carbamate).

Synthesis of 2-(pyridin-2-yldisulfanyl)ethanol

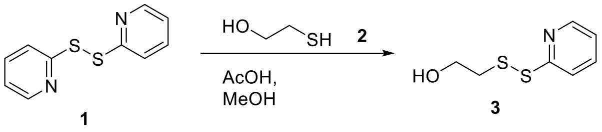

Figure S2. Synthesis and characterization of compound 3, 2-(pyridin-2-yldisulfanyl)ethanol. To a solution of 2-(pyridin-2-yldisulfanyl)pyridine (1) (35 g, 0.158 mol) in 250 ml of methanol, glacial acetic acid (6 ml) was added as catalyst. A solution of 2-sulfanylethanol (2) (12 g, 0.153 mol, 11 ml) in 250 ml of methanol was added dropwise to the above solution at room temperature with continuous stirring. The reaction mixture was stirred at room temperature overnight. Then, the solvent was evaporated to obtain the crude product as a yellow oil. The crude product was purified by flash column chromatography using silica gel as the stationary phase and a mixture of ethyl acetate/hexane as the eluent. The polarity of the eluent was increased by using 40% ethyl acetate/hexane to obtain 2-(pyridin-2-yldisulfanyl)ethanol (25 g, 89%) as a light yellow oil. LC–MS: Rt =1.067 min, (ESI) m/z. [M+H]+ found, 188.0; $C_7H_9NOS_2$ theoretical, 187.01.

Synthesis of (4-nitrophenyl) [2-(pyridin-2-yldisulfanyl)ethyl] carbonate

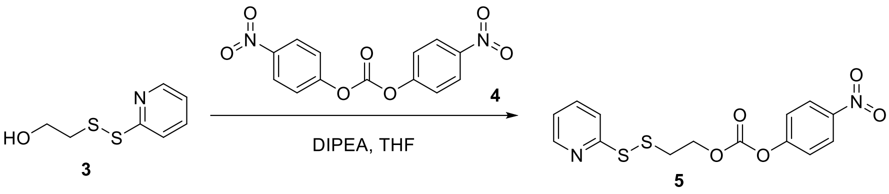

Figure S3. Synthesis and characterization of compound 5, (4-nitrophenyl) [2-(pyridin-2-yldisulfanyl)ethyl] carbonate. A solution of 2-(pyridin-2-yldisulfanyl)ethanol (3) (19 g, 0.1 mol), bis(4-nitrophenyl) carbonate (4) (46.32 g, 0.152 mol) and DIPEA (26 ml, 0.152 mol) in CH_2Cl_2 (500 ml) was placed under Ar protection. The mixture was stirred at room temperature for 5 h. The mixture was washed with water, and the organic phase was dried over $MgSO_4$. The organic solvent was evaporated under reduced pressure, and the residue was purified by flash chromatography (hexane/AcOEt 4:1 to 2:1) to obtain 4-nitrophenyl 2-(pyridin-2-yldisulfanyl)ethyl carbonate (11.21 g, 31.4%) as a light yellow oil. LC–MS: Rt =1.352 min, (ESI) m/z. [M+H]+ found, 353.7; $C_{14}H_{12}N_2O_5S_2$ theoretical, 352.02.

Synthesis of 2-(pyridin-2-yldisulfaneyl)ethyl (2-(ethoxymethyl)-1-(2-hydroxy-2-methylpropyl)-1*H*-imidazo[4,5-*c*]quinolin-4-yl)carbamate

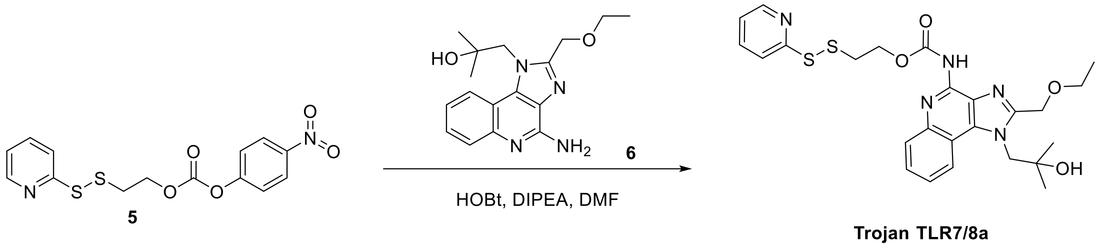

Figure S4. Synthesis and characterization of Trojan TLR7/8a, 2-(pyridin-2-yl-disulfaneyl)ethyl (2-(ethoxymethyl)-1-(2-hydroxy-2-methylpropyl)-1H-imidazo[4,5-c]quinolin-4-yl)carbamate. HOBt (3.22 g, 23.8 mmol), DIPEA (11 ml, 63.6 mmol), and 1-(4-amino-2-(ethoxymethyl)-1H-imidazo[4,5-c]quinolin-1-yl)-2-methylpropan-2-ol (6) (5.00 g, 15.9 mmol) were added to a solution of (4-nitrophenyl) [2-(pyridin-2-yldisulfanyl)ethyl] carbonate (5) (11.21 g, 31.8 mol) in DMF (100 ml). The mixture was stirred at 40 °C for 18 h. LC–MS showed that at this time, the starting material had been completely consumed, and one main peak with the desired mass was detected. The reaction mixture was concentrated under reduced pressure, and the residue was purified by silica gel chromatography by elution with MeOH in DCM (from 0 to 5%) over 10 min to give 2-(pyridin-2-yl-disulfaneyl)ethyl (2-(ethoxymethyl)-1-(2-hydroxy-2-methylpropyl)-1H-imidazo[4,5-c]quinolin-4-yl)carbamate (5.6 g, 67%) as a light yellow gum. LC–MS: Rt = 1.091 min, (ESI) m/z [M+H]+ found, 528.7; $C_{25}H_{29}N_5O_4S_2$ theoretical, 527.17. 1H NMR (400 MHz, CDCl3) δ 8.47 (d, J = 4.8 Hz, 1H), 8.22–8.13 (m, 2H), 7.75 (d, J = 8.0 Hz, 1H), 7.64 (dd, J = 19.2, 7.6 Hz, 2H), 7.51 (t, J = 7.6 Hz, 1H), 7.13–7.05 (m, 1H), 4.94 (s, 2H), 4.81 (s, 2H), 4.55 (t, J = 6.4 Hz, 2H), 3.67 (q, J = 7.2 Hz, 2H), 3.18 (t, J = 6.4 Hz, 2H), 1.35 (s, 6H), 1.25 (d, J = 6.8 Hz, 3H). The purity by HPLC was 98.37% (214 nm) and 98.25% (254 nm).

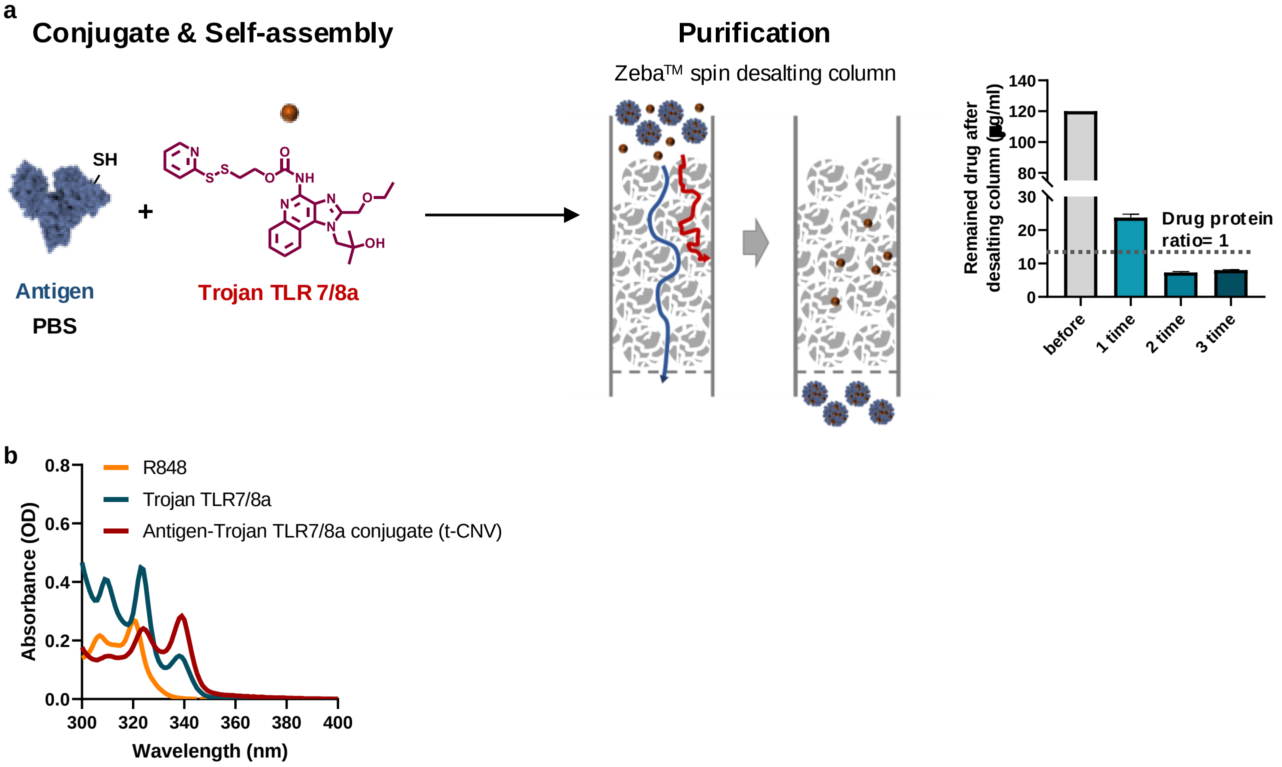

Figure S5. a, Method of purification after the protein antigen and Trojan TLR7/8a conjugate reaction. Purification was performed by desalting (Zeba spin desalting columns, Thermo Fisher) at least twice to remove impurities with small molecular weights. The grey dotted line indicates a drug:protein ratio = 1. **b,** The ultraviolet absorbance of R848, Trojan TLR7/8a and the antigen-TLR7/8a conjugate (t-CNV) at λ = 324 nm. All the data are presented as the mean±s.d.

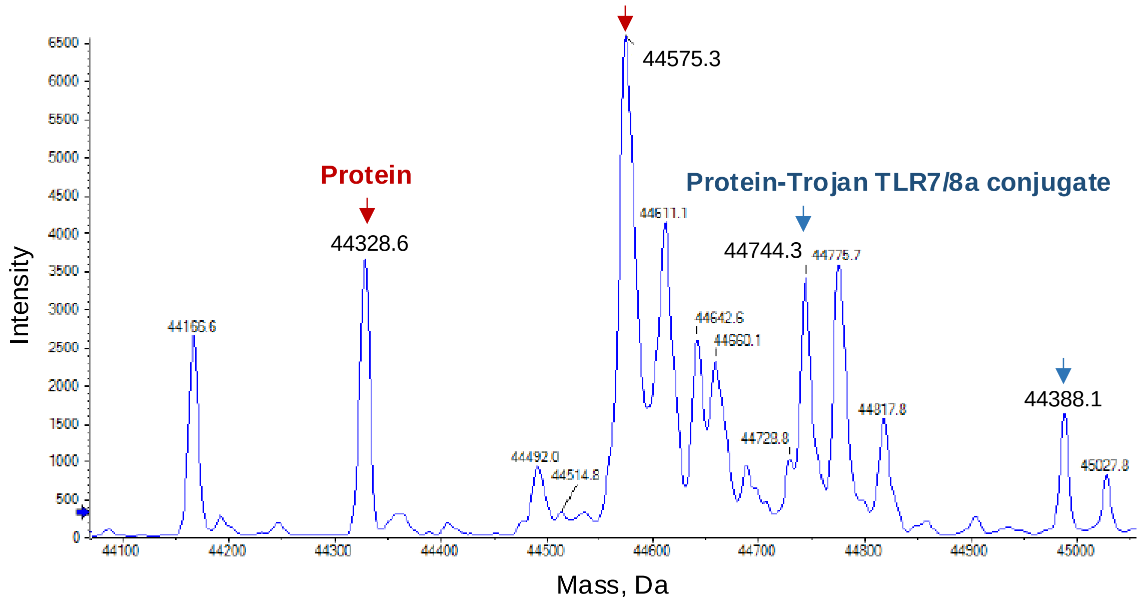

Figure S6. LC–MS analysis of t-CNV (non-particulate) showing peaks based on the addition of 412 g mol-1. Red arrows indicate the peaks of the protein (44328.6 and 44575.3 Da), and blue arrows indicate the peaks of the protein-Trojan TLR7/8a conjugate (44744.3 and 44388.1 Da).

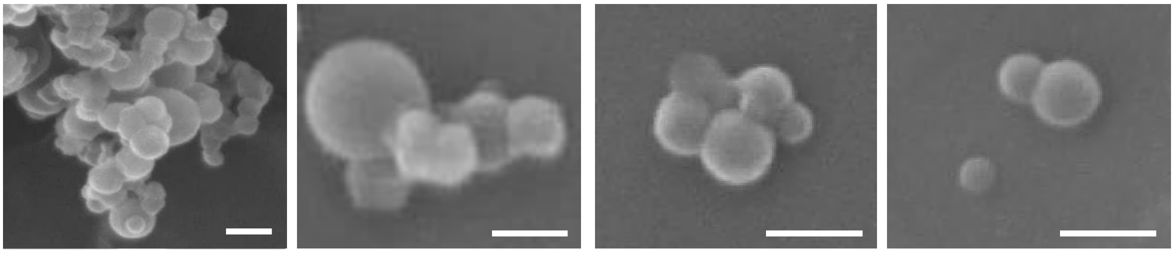

Figure S7. Scanning electron micrographs depicting the t-CNV structure. Scale bars, 100 nm. A representative image is presented in Figure 2d.

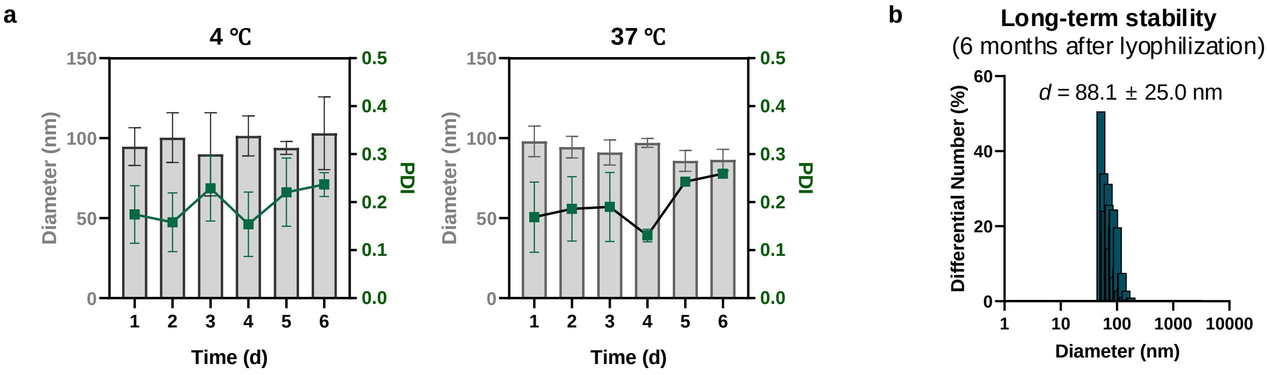

Figure S8. a, Stability of t-CNV in PBS over 6 days at 4 °C and 37 °C (n = 4). **b,** Long-term stability: size of freeze-dried t-CNV after 6 months of storage. All the data are presented as the mean±s.d.

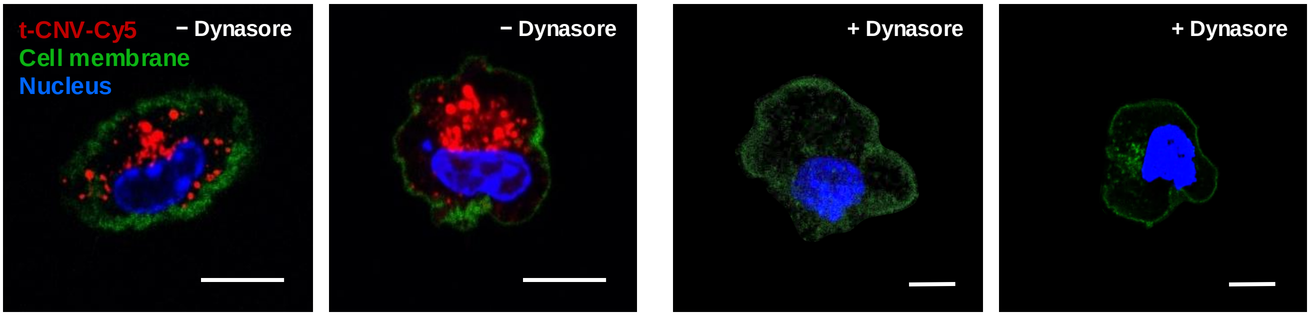

Figure S9. Confocal images of the endocytosis-dependent uptake of t-CNV-Cy5 by BMDCs after 4 h with and without dynasore (a dynamin inhibitor, 40 μM) treatment for 1 h. Cell membrane, wheat germ agglutinin Texas Red (green); nuclei, Hoechst 33342 (blue). Scale bars, 10 μm. A representative image is presented in Figure 2g.

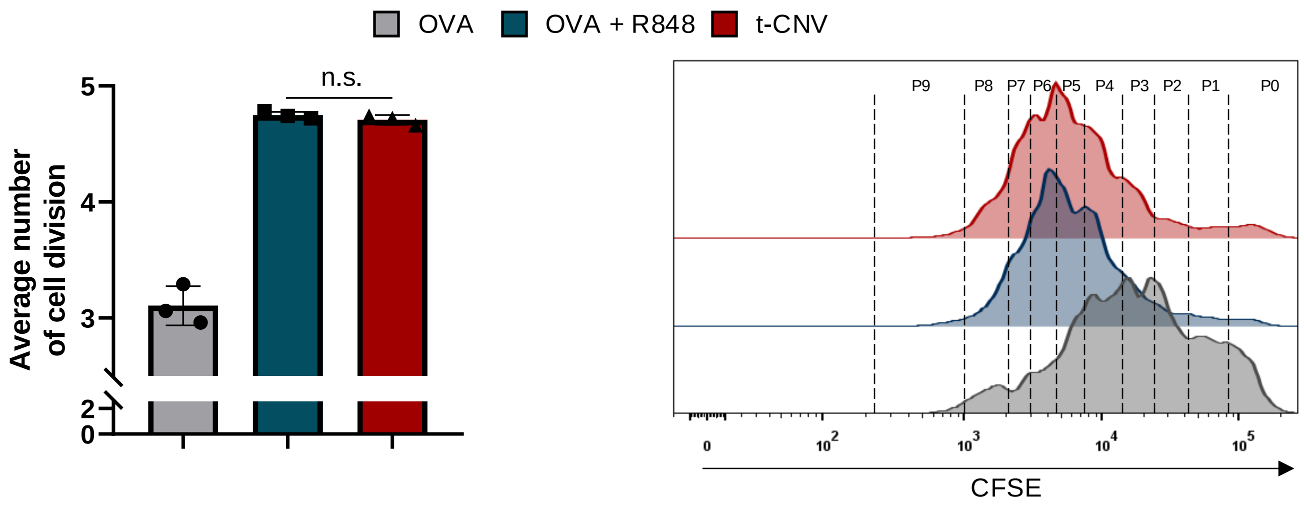

Figure S10. *In vitro* cross-presentation of the OVA protein antigen measured by the proliferation (carboxyfluorescein succinimidyl ester (CFSE) dilution) of OT-1 $CD3^{+}CD8^{+}$ T cells. BMDCs were pulsed with OVA, OVA+R848 or t-CNV (OVA, 6.2 μM; R848, 4.5 μM) for 12 h and cocultured with OT-1 $CD3^{+}CD8^{+}$ T cells for 3 days (left). Statistical analysis of the average number of cell divisions (right). Representative flow cytometry plots of cell division (n = 3). All the data are presented as the mean±s.d. Statistical significance was evaluated by one-way ANOVA with Tukey's multiple comparison test (P values: NS not significant; * P < 0.05, ** P < 0.01, *** P < 0.001, **** P < 0.0001).

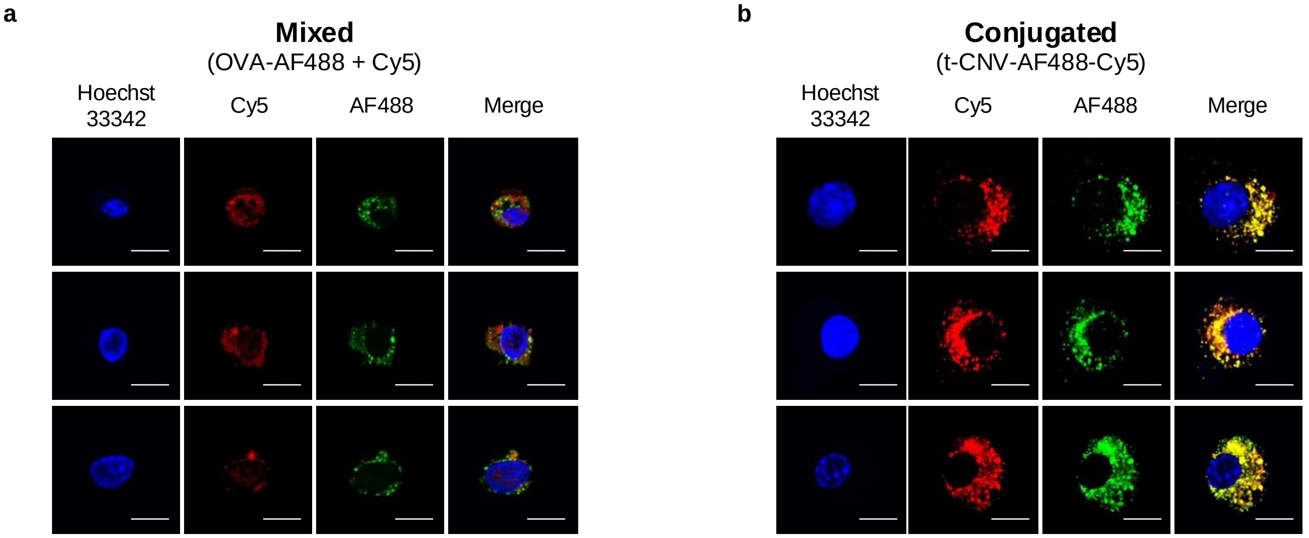

Figure S11. Confocal images of the co-localization of the mixed system (OVA (AF488) + small molecule (Cy5)) and conjugated system (t-CNV-AF488-Cy5) in BMDCs after 4 h of treatment. Scale bars, 10 μm. A representative image is presented in Figure 2q.

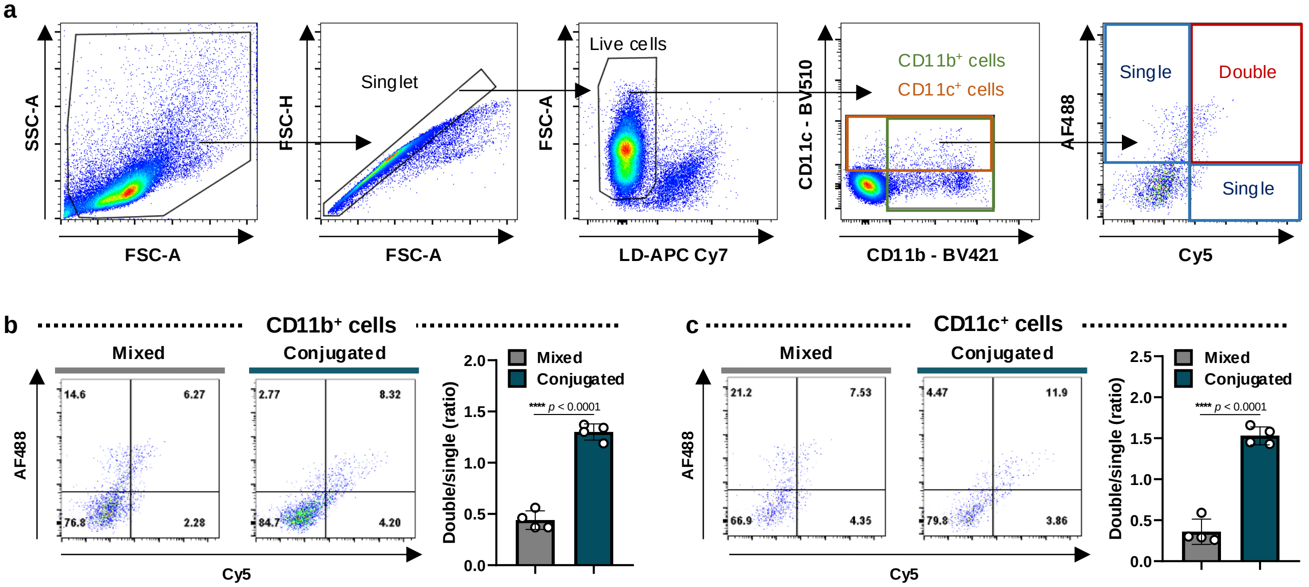

Figure S12. Double and single uptake ratios by cells in the draining lymph node (dLN) 12 h after subcutaneous administration of the mixed protein and small molecule or their conjugated product. **a,** Flow cytometric gating strategy for single (Cy5$^+$ AF488$^-$ or Cy5$^-$ AF488$^+$)- and double (Cy5$^+$ AF488$^+$)-positive cells. **b, c,** Representative dot plots for Figure 2r showing the double and single uptake ratios by CD11b$^+$ cells **(b)** and CD11c$^+$ cells **(c)** (n = 4). All the data are presented as the mean±s.d. Statistical significance was evaluated by an unpaired two-tailed t test in **b, c.** P values: NS, not significant; *P<0.05, **P<0.01, ***P<0.001, ****P<0.0001.

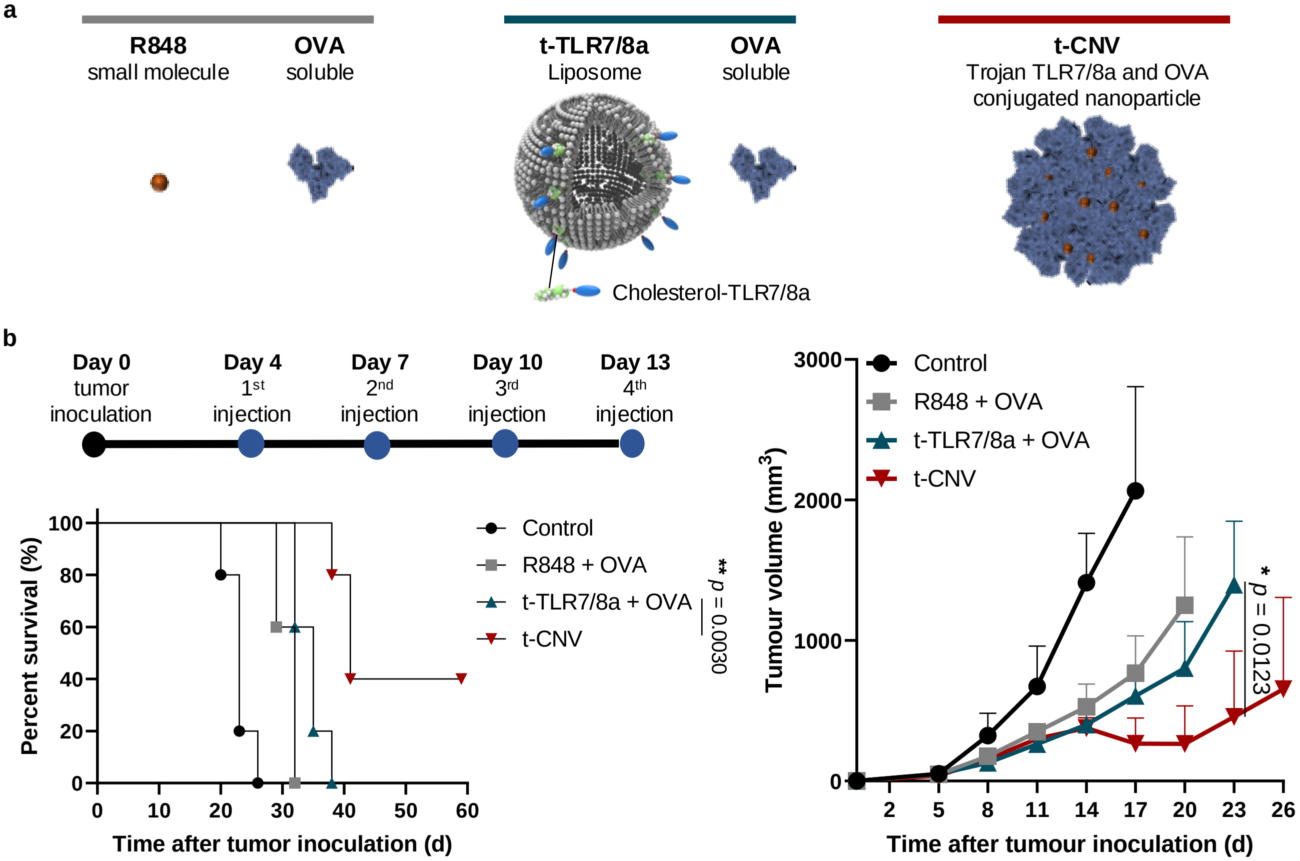

Figure S13. The *in vivo* antitumor efficacy of the antigen and TLR 7/8 agonist is dependent on their physiochemical formulation. a, Depiction of the drug formulation in each group (OVA, 22 nmol; R848, t-TLR7/8a, Trojan TLR7/8a, 16 nmol). G2: mixture of R848 (small molecule TLR7/8 agonist) and OVA (soluble protein); G3: mixture of t-TLR7/8a (timely activating TLR7/8a; liposome-formulated TLR7/8a)[1] and OVA (soluble protein); and G4: t-CNV (Trojan TLR7/8a and OVA conjugated nanoparticle). **b,** Antitumor efficacy in mice with subcutaneous E.G7-OVA tumor (n = 5). All the data are presented as the mean±s.d. Statistical significance was evaluated by an unpaired two-tailed *t*-test in **b**. Survival *P* values were calculated by the log-rank (Mantel-Cox) test in **b**. *P* values: NS, not significant, **P*<0.05, ***P*<0.01, ****P*<0.001, *****P*<0.0001.

Reference

1. Jin, S. M. *et al.* A nanoadjuvant that dynamically coordinates innate immune stimuli activation enhances cancer immunotherapy and reduces immune cell exhaustion. *Nat. Nanotechnol.* **18**, 390–402 (2023).

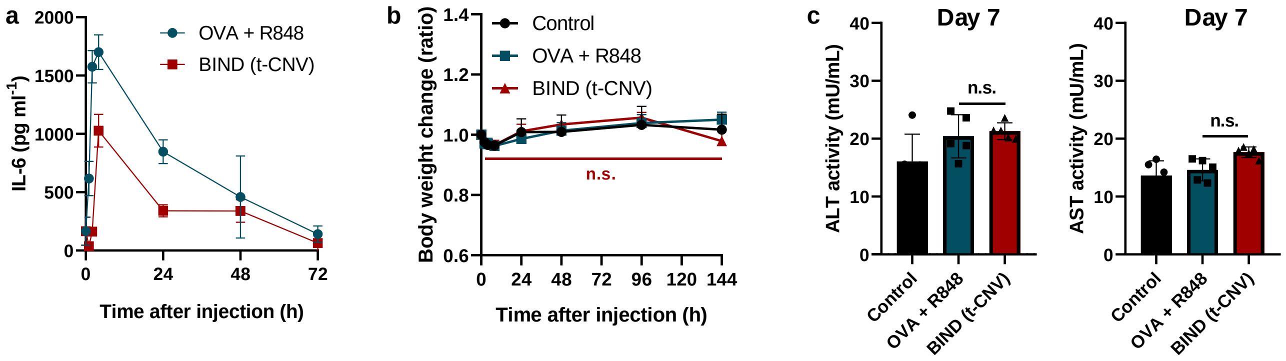

Figure S14. Biosafety analysis of BIND (t-CNV). a, Serum IL-6 level (n = 5). **b,** Time-dependent body weight change over 7 days post-injection (n = 6). **c,** ALT and AST activity analysis at 7 days post-injection (n = 5). All data are presented as the mean ± s.d. Statistical significance was evaluated by an unpaired two-tailed t test in **b** and by one-way ANOVA with Tukey's multiple comparison test in **c**. *P* values: NS, not significant; **P* < 0.05, ***P* < 0.01, ****P* < 0.001, *****P* < 0.0001.

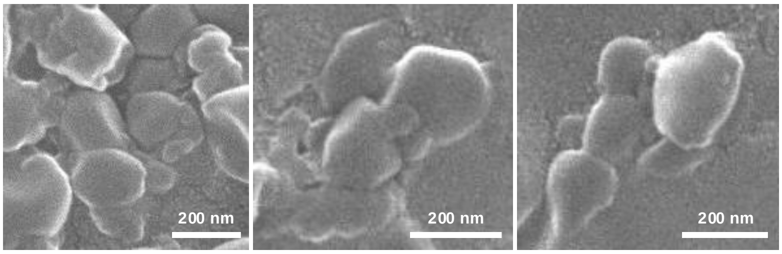

Figure S15. Scanning electron cryomicrographs (SEM) of the structure of TANNylated t-CNV.

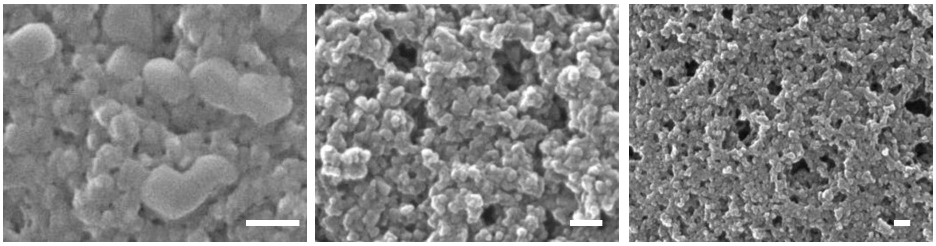

Figure S16. SEM of the structure of BIND (t-CNV). Scale bars, 100 nm. A representative image is presented in Figure 3d.

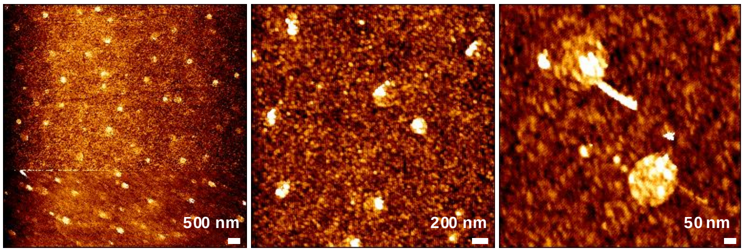

Figure S17. Atomic force microscopy (AFM) image of BIND (t-CNV) in dilution (1/400 X)

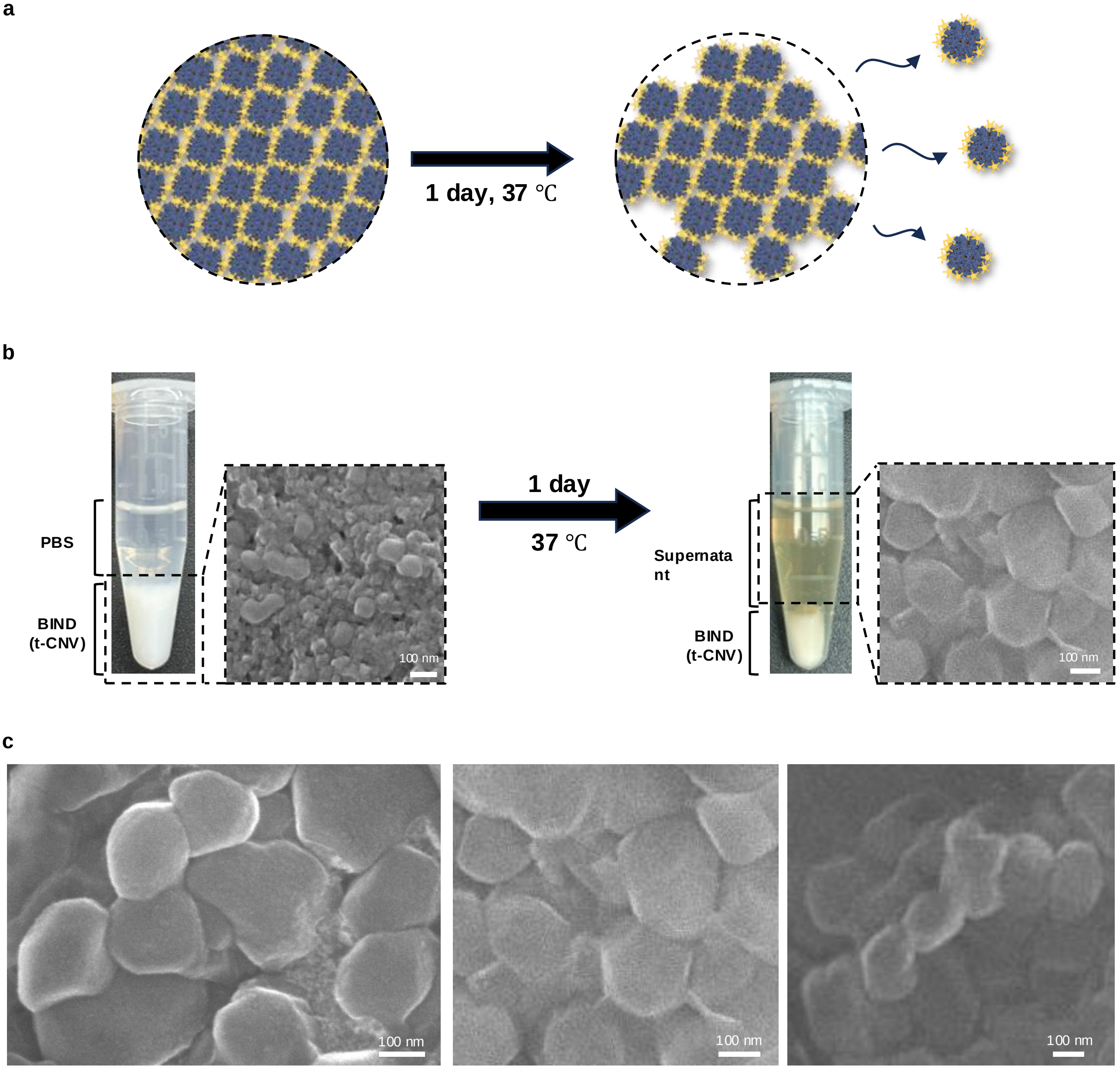

Figure S18. Mechanism of gel formation and particulate release. **a,** Scheme of release mechanism of BIND (t-CNV) after its formation. **b,** *Ex vivo* SEM observation of released particles from BIND (t-CNV) after incubation in PBS for 1 day at 37 ℃. **c,** *Ex vivo* SEM of released particles from BIND (t-CNV) after incubation in PBS for 1 day at 37 ℃. A representative image is presented in Figure S16b.

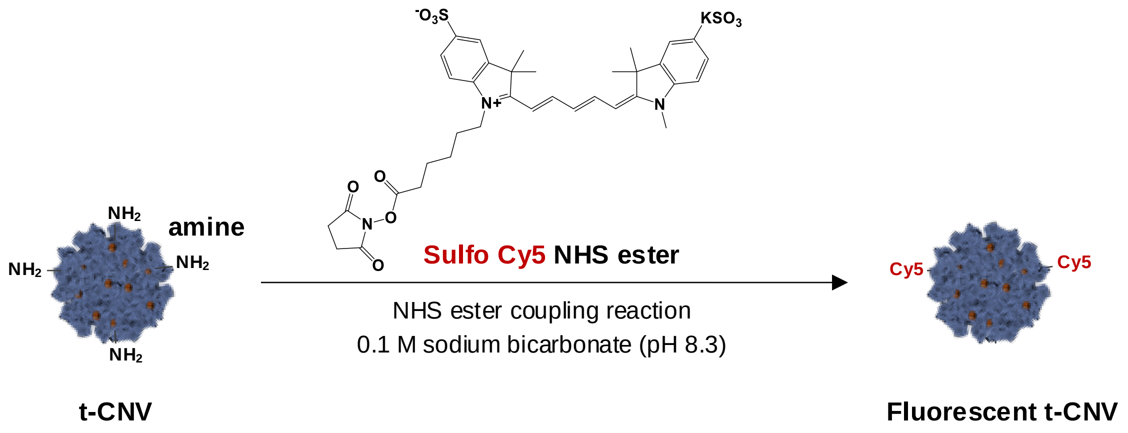

Figure S19. Synthesis and quantification of fluorescence labelling of fluorescent t-CNV.

Synthesis of fluorescent t-CNV:

t-CNV (100 mg mL^{-1}) and sulfo-Cyanine5 NHS ester (10 mg mL^{-1}) were dissolved in 0.1 M sodium bicarbonate (pH 8.3). Sulfo-Cyanine5 NHS ester was added to the t-CNV and reacted 1 h protected from light. The mixture was filtered through the Pierce™ dye removal columns (Thermo Fisher Scientific) twice to remove unlabeled free dyes.

Quantification of fluorescence labeling:

The number of fluorophores per t-CNV was estimated by Beer-Lambert law:

$$C_{dye} = \frac{A_{dye}}{\varepsilon_{dye} b}$$

A_{dye} is the absorbance of the fluorescent t-CNV at λ = 646 nm, ε_{dye} is the molar absorptivity of sulfo-Cyanine5 NHS ester at λ = 646 nm (ε_{dye} = 271,000 L mol^{-1} cm^{-1}), and b is the path length of the light (b = 1 cm).

A_{dye} was quantified with UV-Vis spectrometer and it is to be 0.614 (1000-fold diluted).

$$C_{dye} = \frac{A_{dye}}{(271{,}000\frac{L}{mol\,cm})(1\,cm)} \times 1000 = 2.27 \text{ mM}$$

The number of fluorophores per t-CNV was determined as:

$$\frac{C_{dye}}{C_{protein}} = 1.11$$

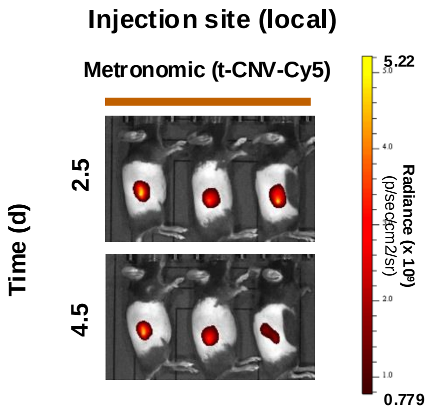

Figure S20. IVIS images of the fluorescence signal of the metronomic (t-CNV-Cy5) at the injection site over time in wild-type C57BL/6 mice (n = 3).

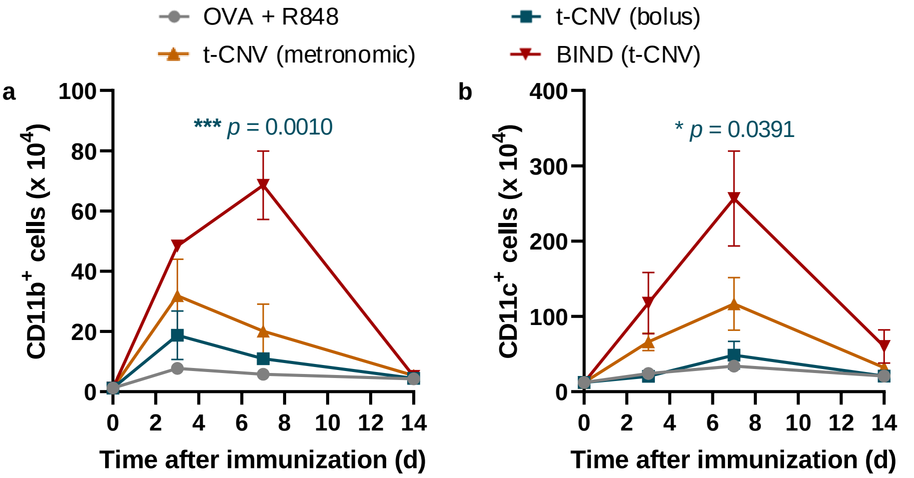

Figure S21. *In vivo* kinetics of innate immune cells: macrophages ($CD11b^{+}$) **(a)** and DCs ($CD11c^{+}$) **(b)** in the TDLN following peritumoral injection of the indicated samples (OVA, 22 nmol; R848, Trojan TLR7/8a, 16 nmol) to B16-OVA tumor-bearing mice (n = 3). All the data are presented as the mean±s.d. Statistical significance was evaluated by an unpaired two-tailed t test (P values: NS not significant; * $P < 0.05$, ** $P < 0.01$, *** $P < 0.001$, **** $P < 0.0001$).

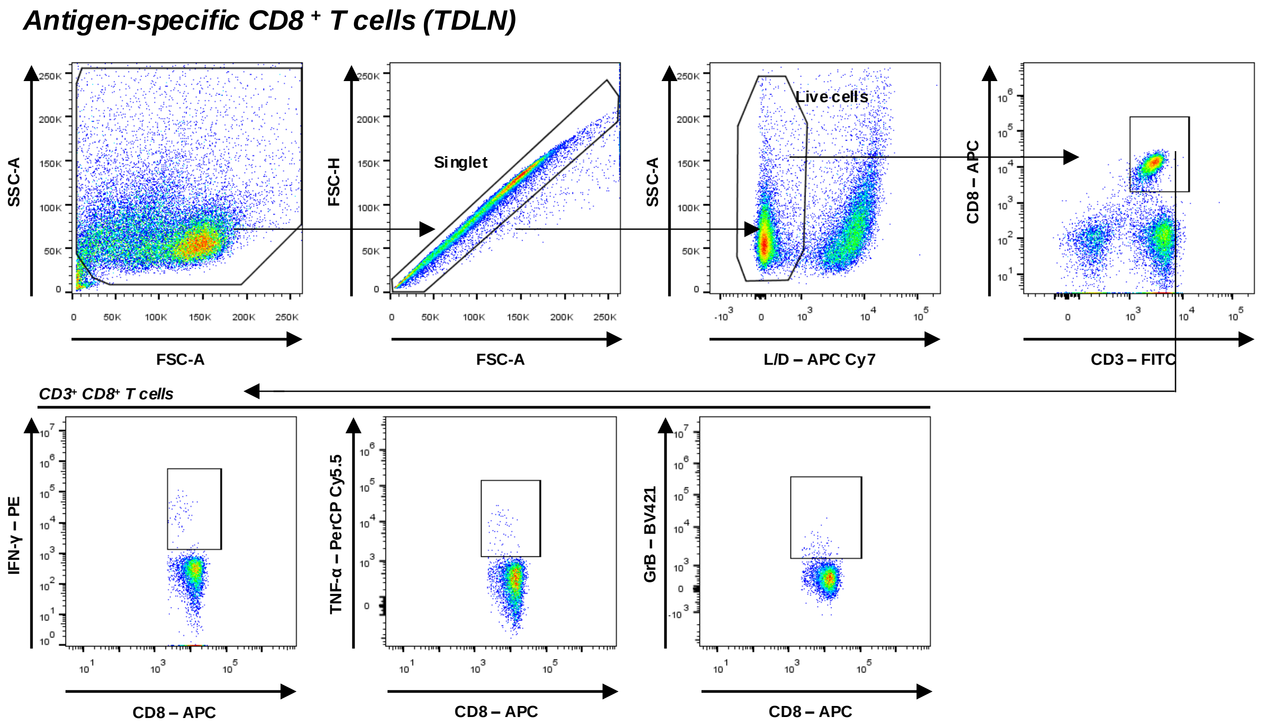

Figure S22. Flow cytometry gating strategy for Figure 4h and Figure S23 illustrating the analysis of immune cells *in vivo* in the B16-OVA tumor model. Populations of IFN-γ^+-, TNF-α^+- or GrB^+-producing $CD8^+$ T cells ($CD3^+CD8^+$) in the TDLN. The gating strategy for all samples was set to remove large clumps or aggregates of cells (FSC-H and FSC-A gating), cell debris, and dead cells (live/dead gating).

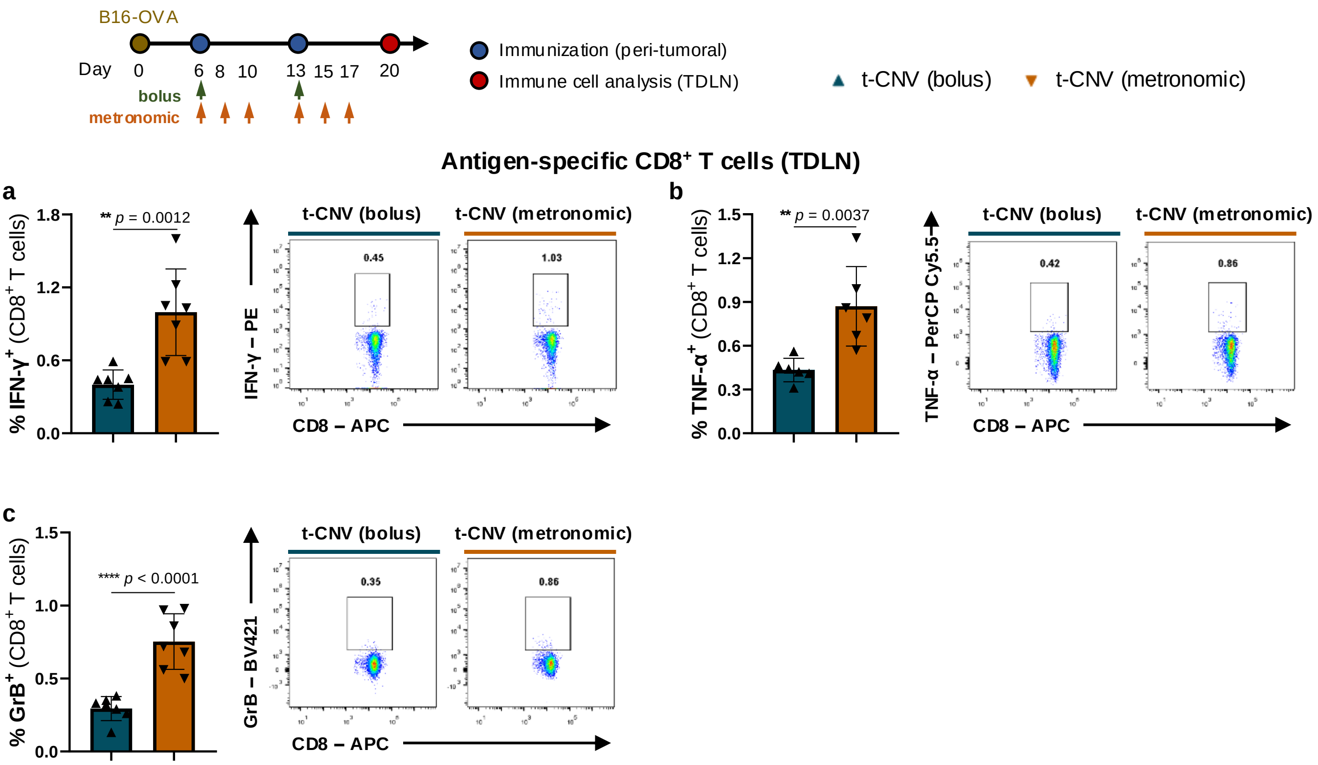

Figure S23. *In vivo* kinetics of vaccine availability (bolus versus metronomic) dependent on the generation of antigen-specific CD8$^+$ T cells in the TDLN in B16-OVA-tumor bearing mice (n = 6-7). t-CNV (OVA, 22 nmol; Trojan TLR7/8a, 16 nmol) was peritumorally injected via either a bolus or in a metronomic manner. **a-c,** Representative flow cytometry dot plots and percentages of **(a)** IFN-γ$^+$, **(b)** TNF-α$^+$ and **(c)** GrB$^+$ cells in CD8$^+$ T cells (CD3$^+$CD8$^+$) after SIINFEKL peptide-restimulation and incubation with GolgiPlug for 6 h. All the data are presented as the mean±s.d. Statistical significance was evaluated by an unpaired two-tailed *t* test (*P* values: NS not significant; * $P < 0.05$, ** $P < 0.01$, *** $P < 0.001$, **** $P < 0.0001$).

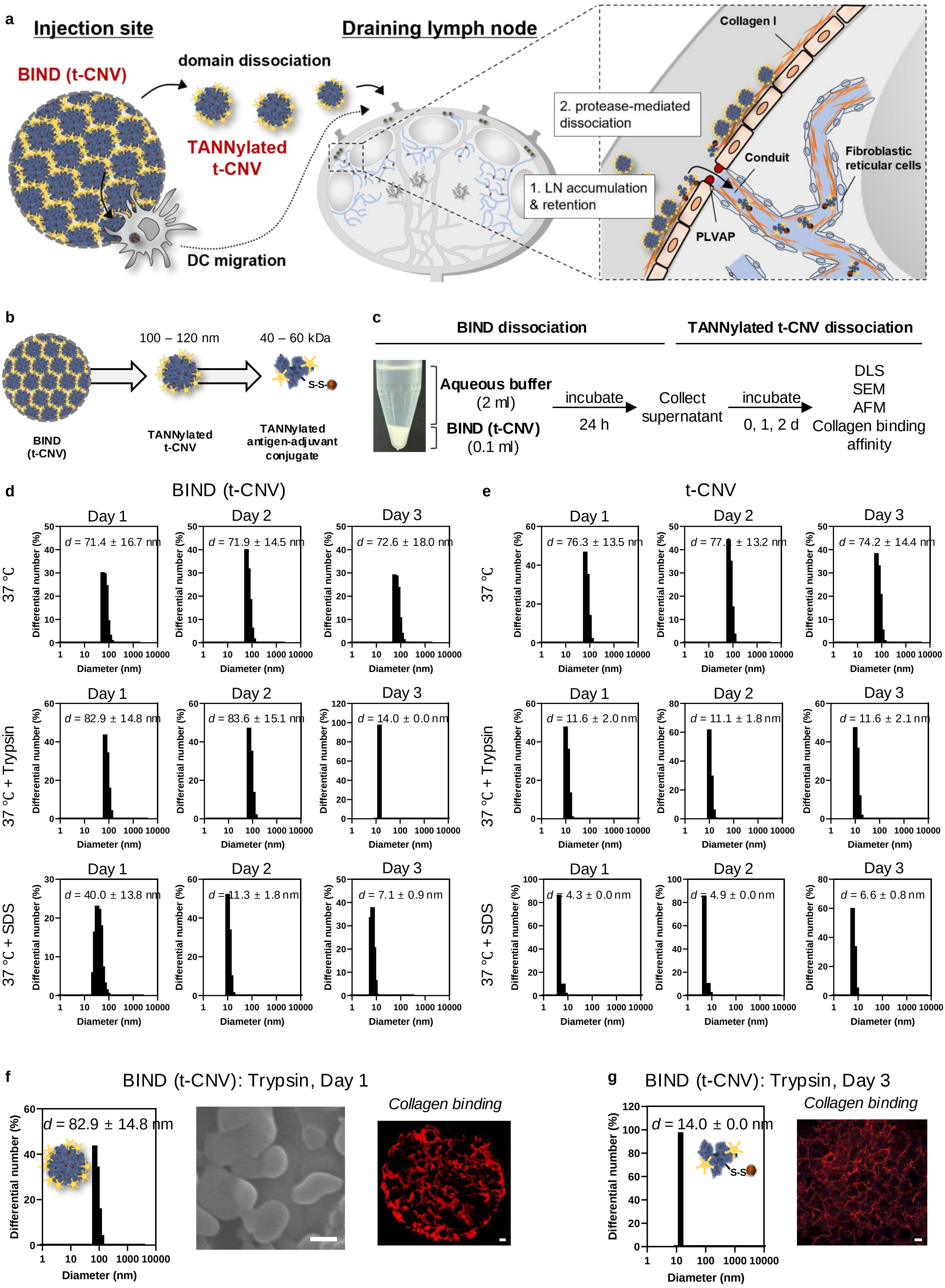
a
Injection site
Draining lymph node
BIND (t-CNV)
domain dissociation
TANNylated t-CNV
DC migration
Collagen I
2. protease-mediated dissociation
Conduit
Fibroblastic reticular cells
1. LN accumulation & retention
PLVAP
b
100 – 120 nm
40 – 60 kDa
S-S
BIND (t-CNV)
TANNylated t-CNV
TANNylated antigen-adjuvant conjugate
c
BIND dissociation
TANNylated t-CNV dissociation
Aqueous buffer (2 ml)
BIND (t-CNV) (0.1 ml)
incubate
24 h
Collect supernatant
incubate
0, 1, 2 d
DLS
SEM
AFM
Collagen binding affinity
d
BIND (t-CNV)
e
t-CNV
Day 1
Day 2
Day 3
37 °C
37 °C + Trypsin
37 °C + SDS
Differential number (%)
Diameter (nm)
d = 71.4 ± 16.7 nm
d = 71.9 ± 14.5 nm
d = 72.6 ± 18.0 nm
d = 76.3 ± 13.5 nm
d = 77.[illegible] ± 13.2 nm
d = 74.2 ± 14.4 nm
d = 82.9 ± 14.8 nm
d = 83.6 ± 15.1 nm
d = 14.0 ± 0.0 nm
d = 11.6 ± 2.0 nm
d = 11.1 ± 1.8 nm
d = 11.6 ± 2.1 nm
d = 40.0 ± 13.8 nm
d = 11.3 ± 1.8 nm
d = 7.1 ± 0.9 nm
d = 4.3 ± 0.0 nm
d = 4.9 ± 0.0 nm
d = 6.6 ± 0.8 nm
f
BIND (t-CNV): Trypsin, Day 1
d = 82.9 ± 14.8 nm
Collagen binding
g
BIND (t-CNV): Trypsin, Day 3
d = 14.0 ± 0.0 nm
S-S
Collagen binding

Figure S24. a, Illustration of the delivery mechanism of BIND (t-CNV) from the injection site to the conduit-mediated paracortex in the draining lymph node. **b,** Illustration of the step-by-step dissociation of BIND (t-CNV) into TANNylated t-CNV and into the TANNylated antigen-adjuvant conjugate. **c,** Experimental scheme to evaluate the temperature-, protease (trypsin)-, and surfactant (SDS)-dependent dissociation of BIND (t-CNV). BIND (t-CNV) was incubated at 37 °C in aqueous buffer with or without trypsin or SDS for 24 h. Then, the supernatant containing TANNylated t-CNV was further incubated for 1 or 2 days. **d,** Representative plot of data presented in Figure 4e. DLS analysis of BIND (t-CNV) after incubation at 37 °C in aqueous buffer with or without trypsin or SDS. **e,** Representative plot of data presented in Fig 4e. DLS of t-CNV after incubated at 37 °C in an aqueous buffer with or without trypsin or SDS. **f-g,** DLS and SEM analysis and confirmation of collagen binding of BIND (t-CNV) after incubation at 37 °C in aqueous buffer with trypsin for 1 day **(f)** or 3 days **(g)**. Scale bar, 100 nm.

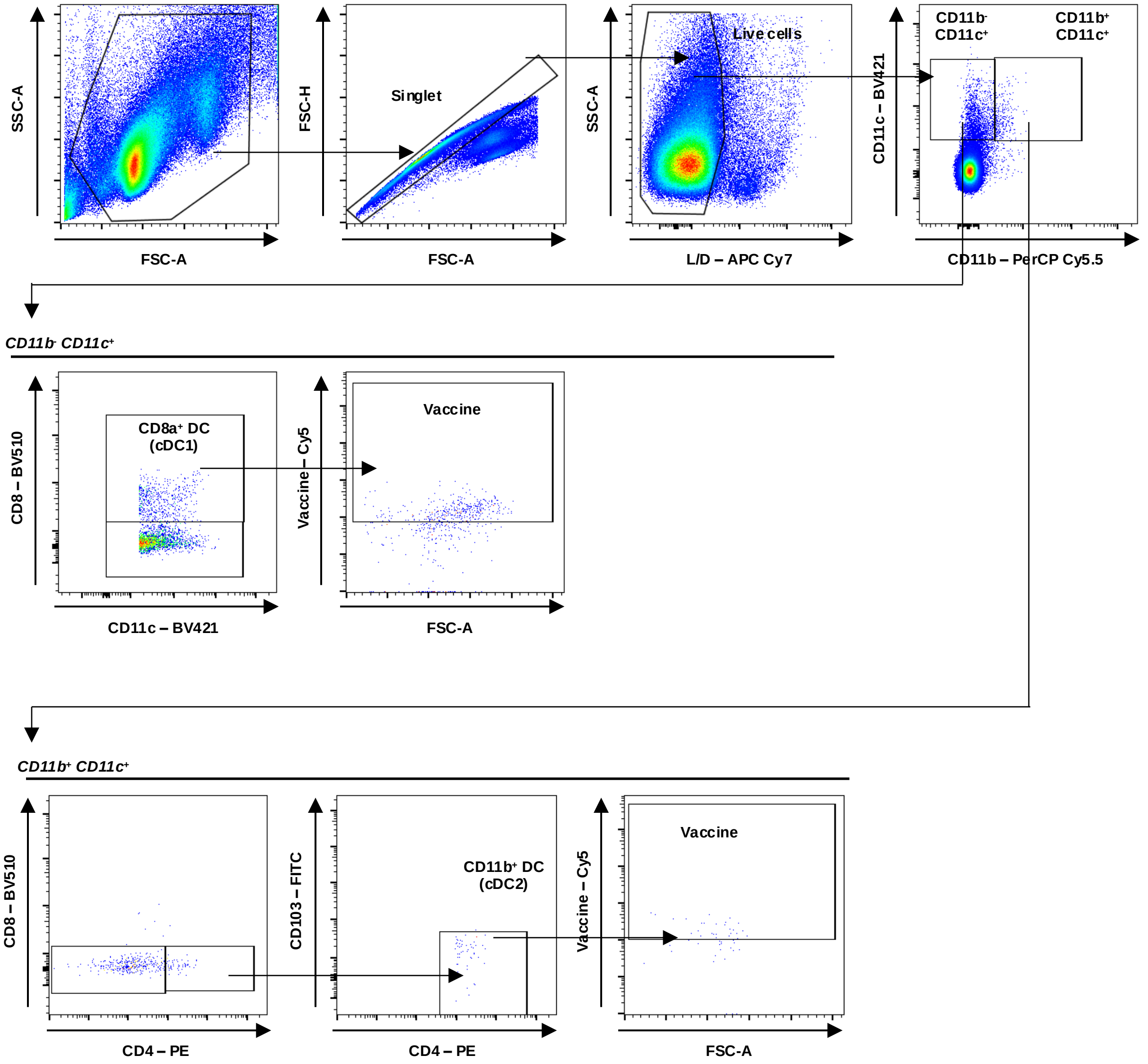

Figure S25. Flow cytometry gating strategy for Figure 4g illustrating the kinetics of the percentage of vaccine^{+} APCs in the TDLN: cDC1s (CD11b^{-}CD11c^{+}CD8^{+}) and cDC2s (CD11b^{+}CD11c^{+}CD8^{-}CD4^{+}CD103^{-}). The gating strategy for all samples was set to remove large clumps or aggregates of cells (FSC-H and FSC-A gating), cell debris, and dead cells (live/dead gating).

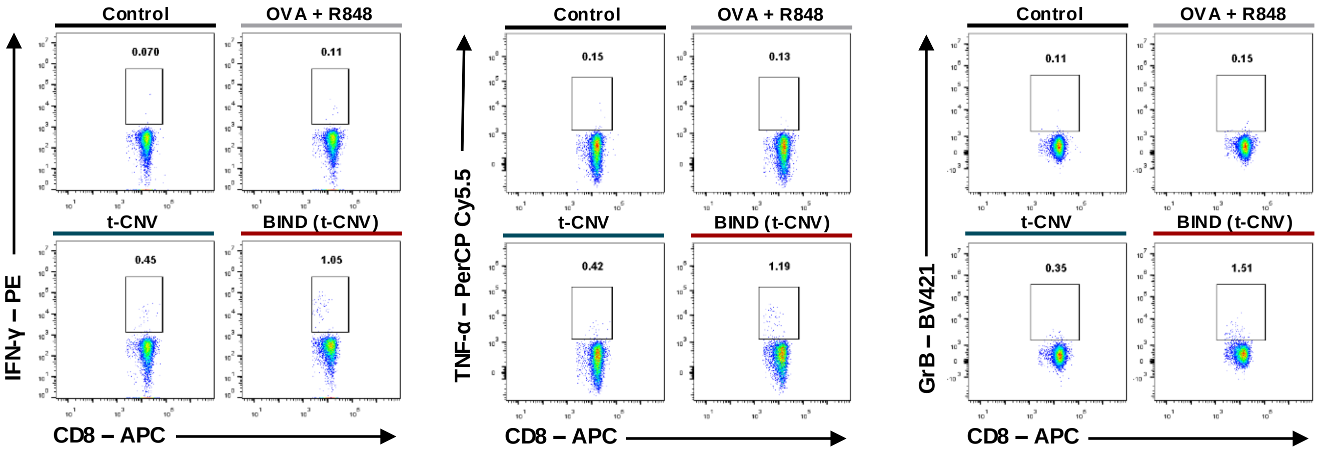

Figure S26. Representative dot plots for Figure 4h showing antigen-specific CD8+ T cells in the TDLN after SIINFEKL peptide-restimulation and incubation with GolgiPlug for 6 h

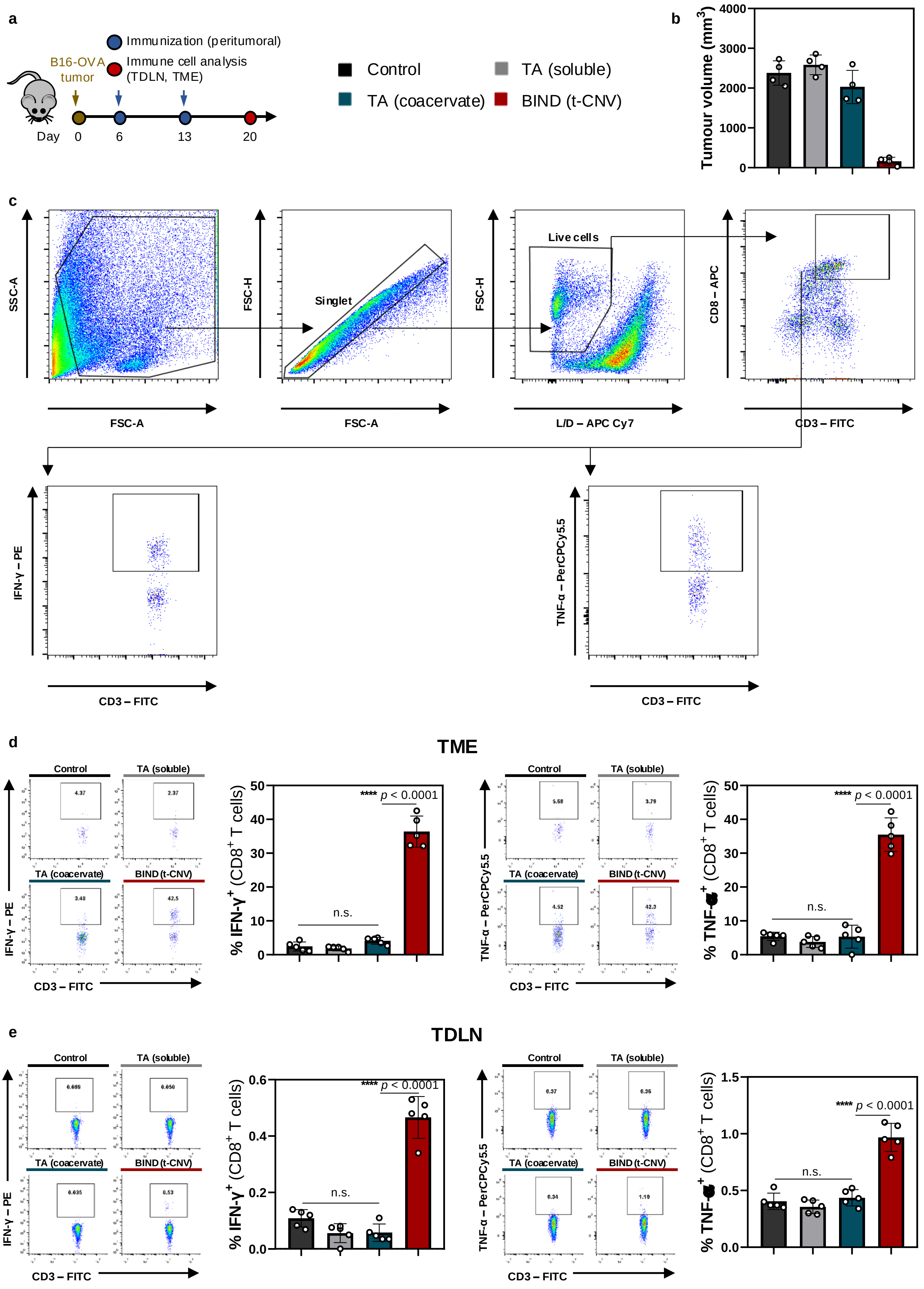

a
Immunization (peritumoral)
Immune cell analysis (TDLN, TME)
B16-OVA tumor
Day 0 6 13 20
Control
TA (soluble)
TA (coacervate)
BIND (t-CNV)
b
Tumour volume (mm3)
c
SSC-A
FSC-A
FSC-H
Singlet
Live cells
L/D – APC Cy7
CD8 – APC
CD3 – FITC
IFN-γ – PE
TNF-α – PerCPCy5.5
d
TME
% IFN-γ+ (CD8+ T cells)
% TNF-α+ (CD8+ T cells)
**** p < 0.0001
n.s.
e
TDLN

Figure S27. Immunogenicity of TA in its soluble form or during coacervate formation. TA (in its soluble form) was administered in an equivalent amount to that present in BIND (t-CNV). TA (coacervate) was formed by the simple mixing of TA with human serum albumin (HSA), following the same protocol used for the fabrication of BIND (t-CNV). **a,** Schedule of immune cell analysis in the TDLN and TME (OVA, HSA, 22 nmol; Trojan TLR7/8a, 16 nmol; TA, 620 μg). **b,** Tumor volume 20 days after tumor inoculation. **c,** Flow cytometry gating strategy for the populations of IFN-γ^+ or TNF-α^+ producing $CD8^+$ T cells ($CD3^+CD8^+$). The gating strategy for all samples was set to remove large clumps or aggregates of cells (FSC-H and FSC-A gating), cell debris, and dead cells (live/dead gating). **d-e,** Representative flow cytometry dot plots and percentage of antigen-specific $CD8^+$ T cells after SIINFEKL peptide-restimulation and incubation with GolgiPlug for 6 h in the TME **(d)** and TDLNs **(e)** (n = 5). All data are presented as the mean ± s.d. Statistical significance was evaluated by one-way ANOVA with Tukey's multiple comparison test in **d and e**. *P* values: NS, not significant, **P*<0.05, ***P*<0.01, ****P*<0.001, *****P*<0.0001.

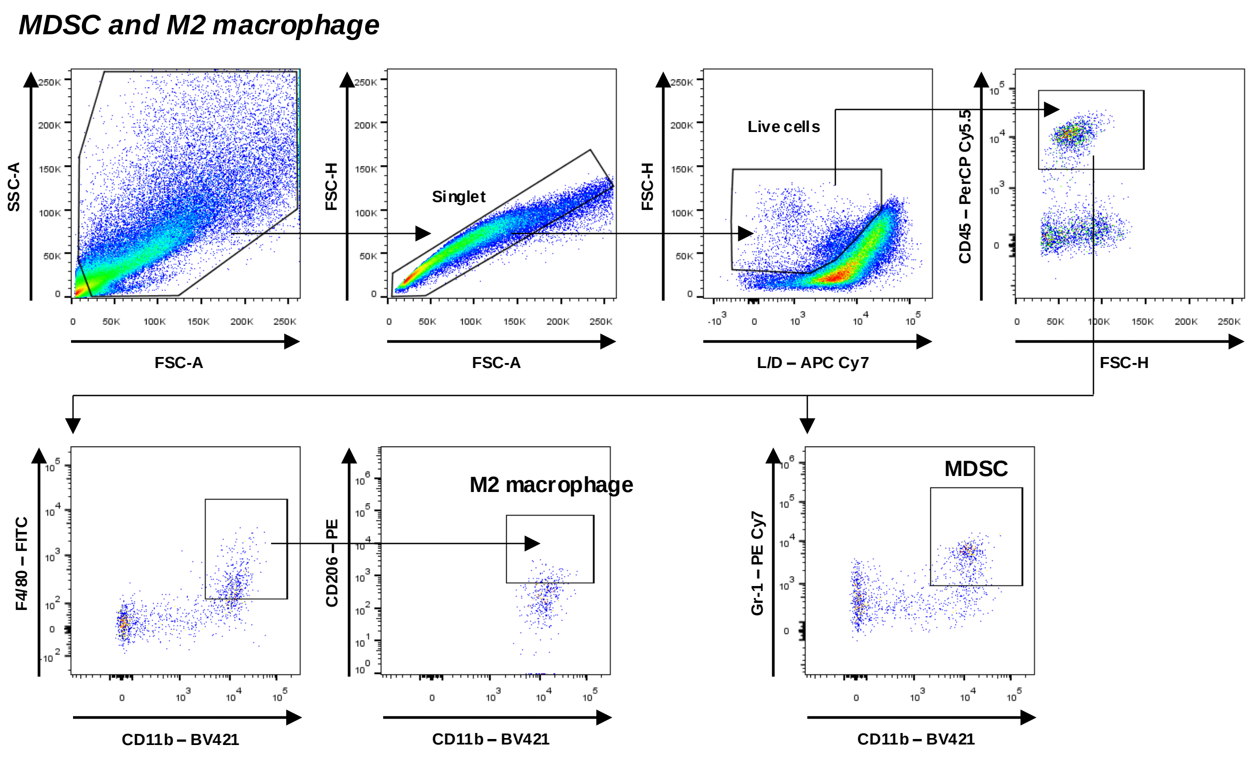

Figure S28. Flow cytometry gating strategy for Figure 5e illustrating the analysis of immunosuppressive cells *in vivo* in the B16-OVA tumor model. Populations of M2 macrophages (CD11b$^+$F4/80$^+$CD206$^+$ gated in CD45$^+$) and MDSCs (CD11b$^+$Gr-1$^+$ gated in CD45$^+$) in the TME. The gating strategy for all samples was set to remove large clumps or aggregates of cells (FSC-H and FSC-A gating), cell debris, and dead cells (live/dead gating).

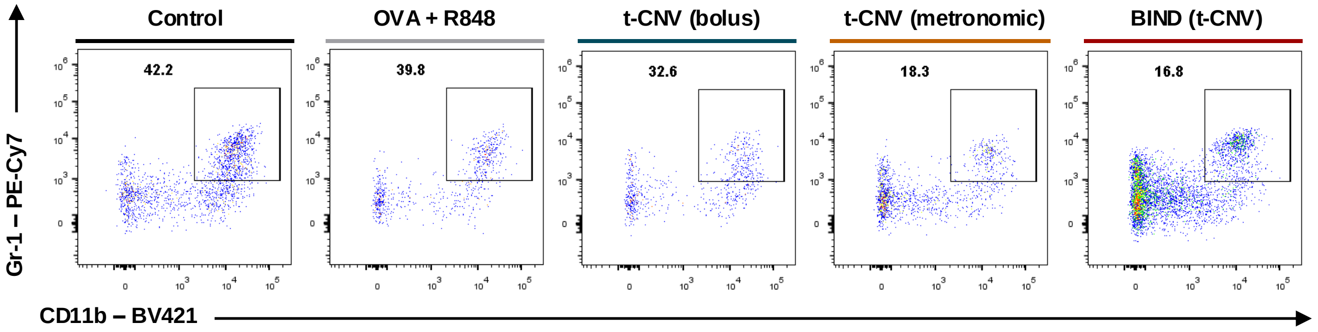

Figure S29. Representative dot plots for Figure 5e showing the MDSCs in the TME after the administration of OVA+R848, t-CNV (bolus), t-CNV (metronomic) or BIND (t-CNV).

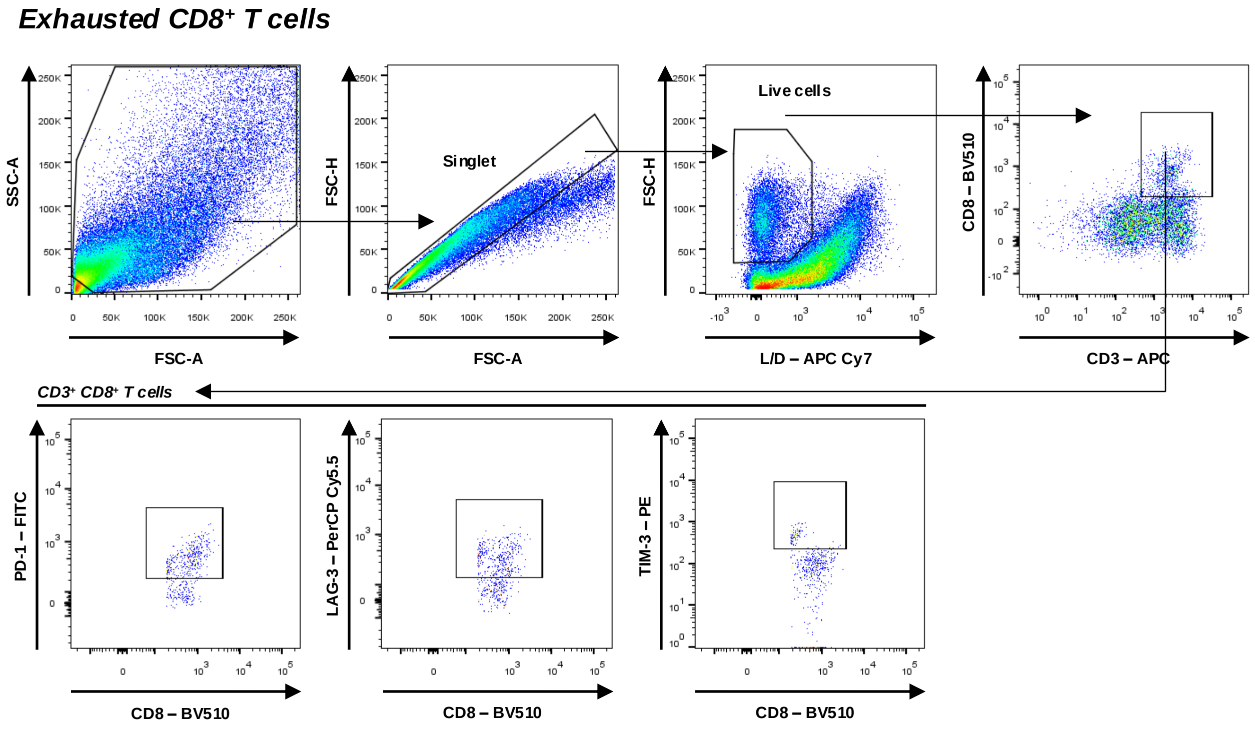

Figure S30. Flow cytometry gating strategy for Figure 5f, illustrating the analysis of exhausted CD8+ cells *in vivo* in the B16-OVA tumor model. Populations of exhausted CD8+ T cells (PD-1+ or LAG-3+ or TIM-3+ in CD3+CD8+) in the TME. The gating strategy for all samples was set to remove large clumps or aggregates of cells (FSC-H and FSC-A gating), cell debris, and dead cells (live/dead gating).

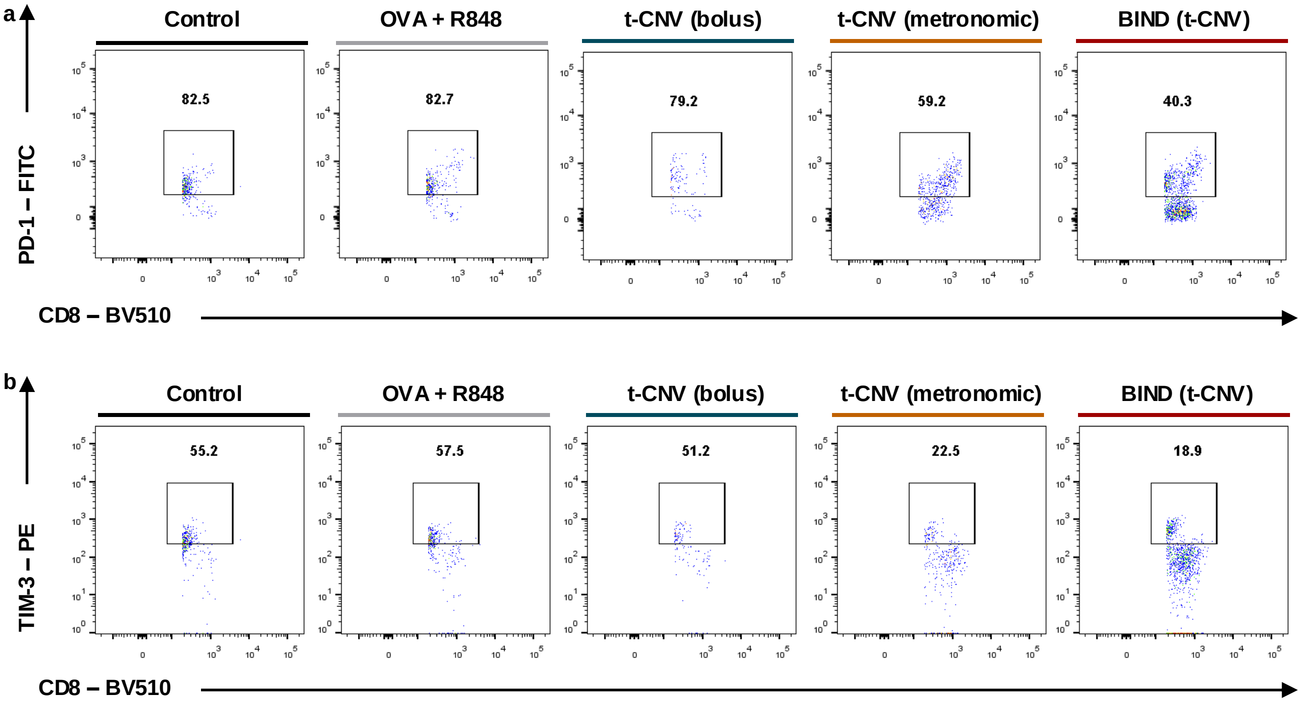

Figure S31. Representative dot plots for Figure 5f showing exhausted CD8+ T cells characterized by the expression of PD-1 **(a)** and TIM-3 **(b)** in the TME after the administration of OVA+R848, t-CNV (bolus), t-CNV (metronomic) or BIND (t-CNV).

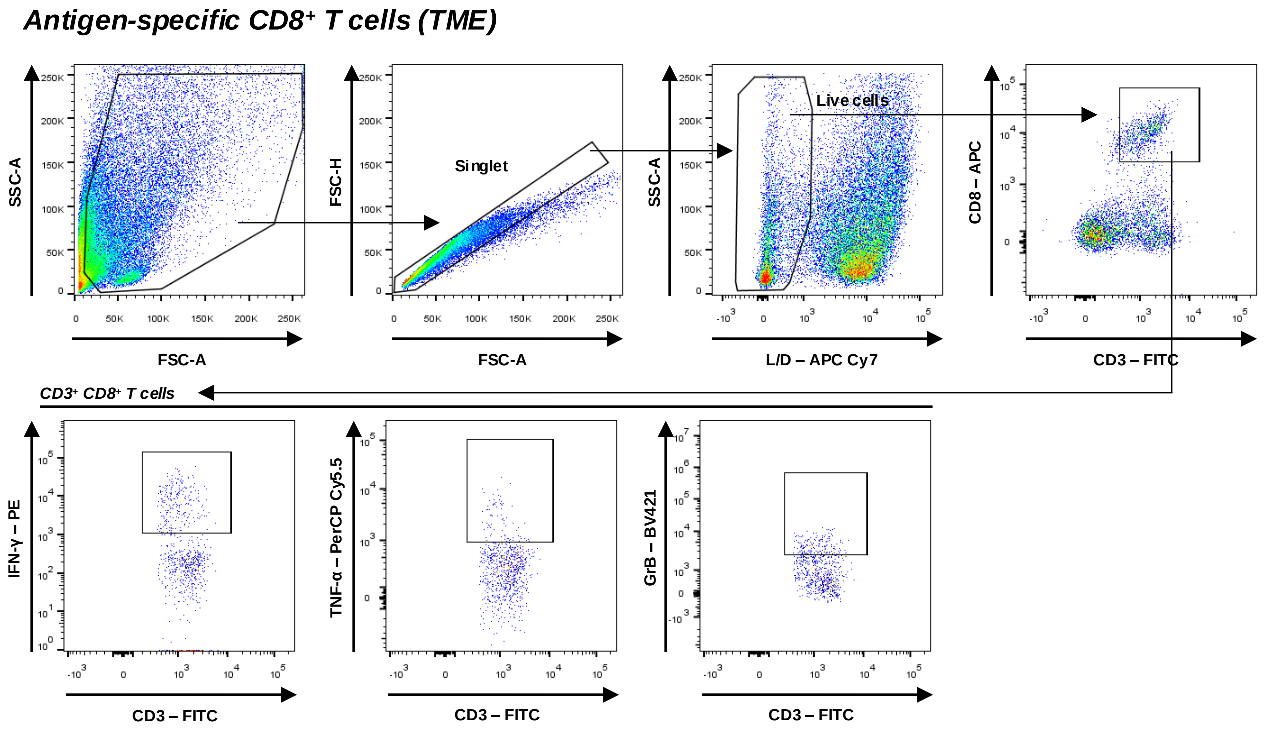

Figure S32. Flow cytometry gating strategy for Figure 5g illustrating the analysis of immune cells *in vivo* in the B16-OVA tumor model. Populations of IFN-γ^{+}-, TNF-α^{+}- or GrB^{+}- producing CD8^{+} T cells (CD3^{+}CD8^{+}) in the TME. The gating strategy for all samples was set to remove large clumps or aggregates of cells (FSC-H and FSC-A gating), cell debris, and dead cells (live/dead gating).

NK cells

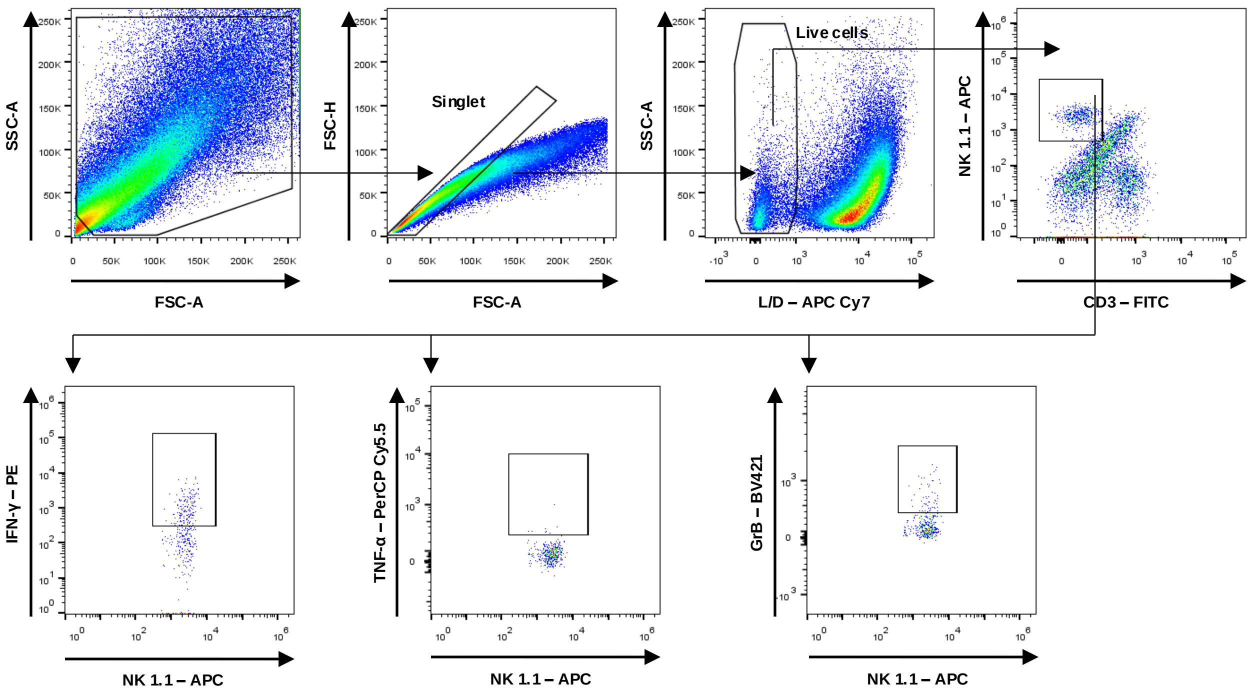

Figure S33. Flow cytometry gating strategy for Figure 5h, illustrating the analysis of activated NK cells *in vivo* in the B16-OVA tumor model. Populations of activated NK cells (IFN-γ^+, TNF-α^+ or GrB$^+$ gated in CD3$^-$NK1.1$^+$ NK cells) in the TME. The gating strategy for all samples was set to remove large clumps or aggregates of cells (FSC-H and FSC-A gating), cell debris, and dead cells (live/dead gating).

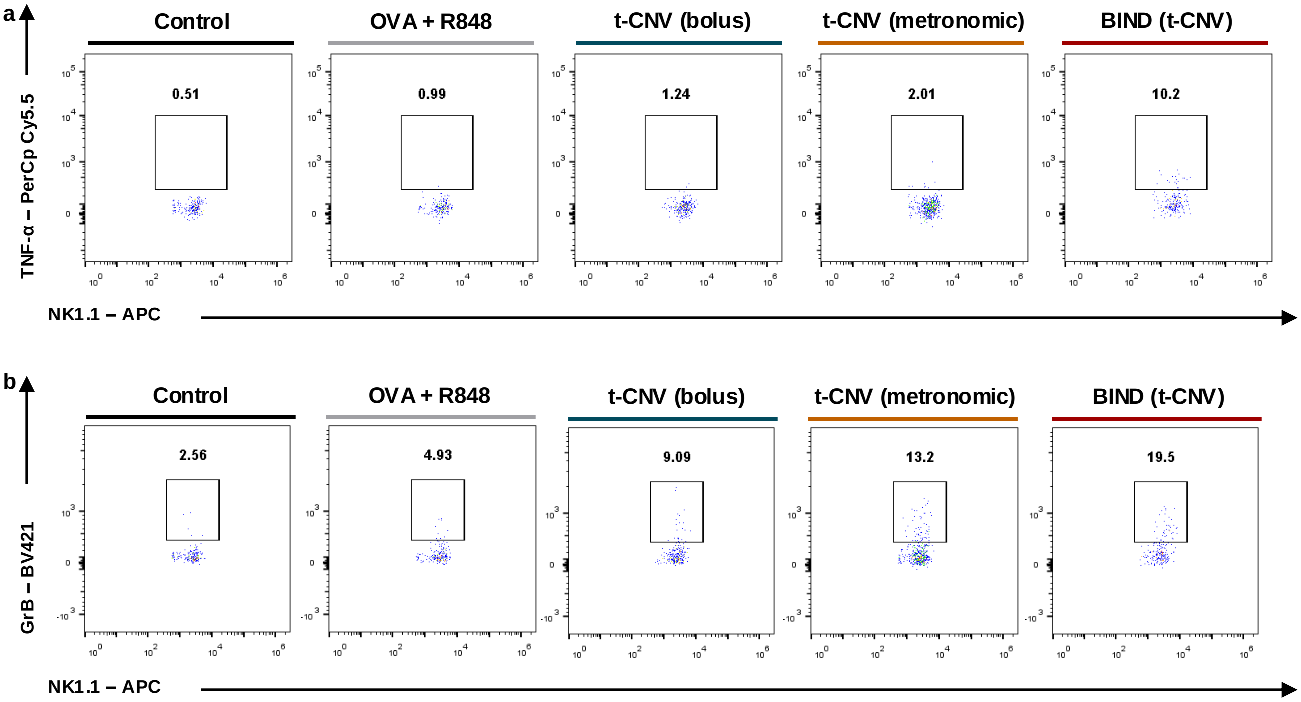

Figure S34. Representative flow cytometry dot plots for Figure 5h showing activated NK cells characterized by the TNF-α^+ **(a)** and GrB^+ **(b)** expression after incubation with a cell activation cocktail (with brefeldin A), mixture of optimized concentrations of PMA, ionomycin and brefeldin A for 4 h.

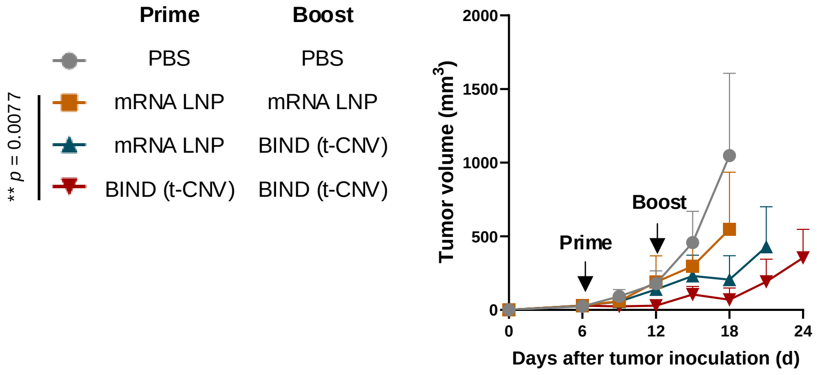

Figure S35. Comparison of the antitumor efficacy comparison with mRNA LNP vaccine in the B16-OVA melanoma tumor model. Tumor growth curves following intramuscular (mRNA LNP) or peritumoral (BIND (t-CNV)) immunization (OVA, 22 nmol; Trojan TLR7/8a, 16 nmol; mRNA (5 μg)) (n = 7).

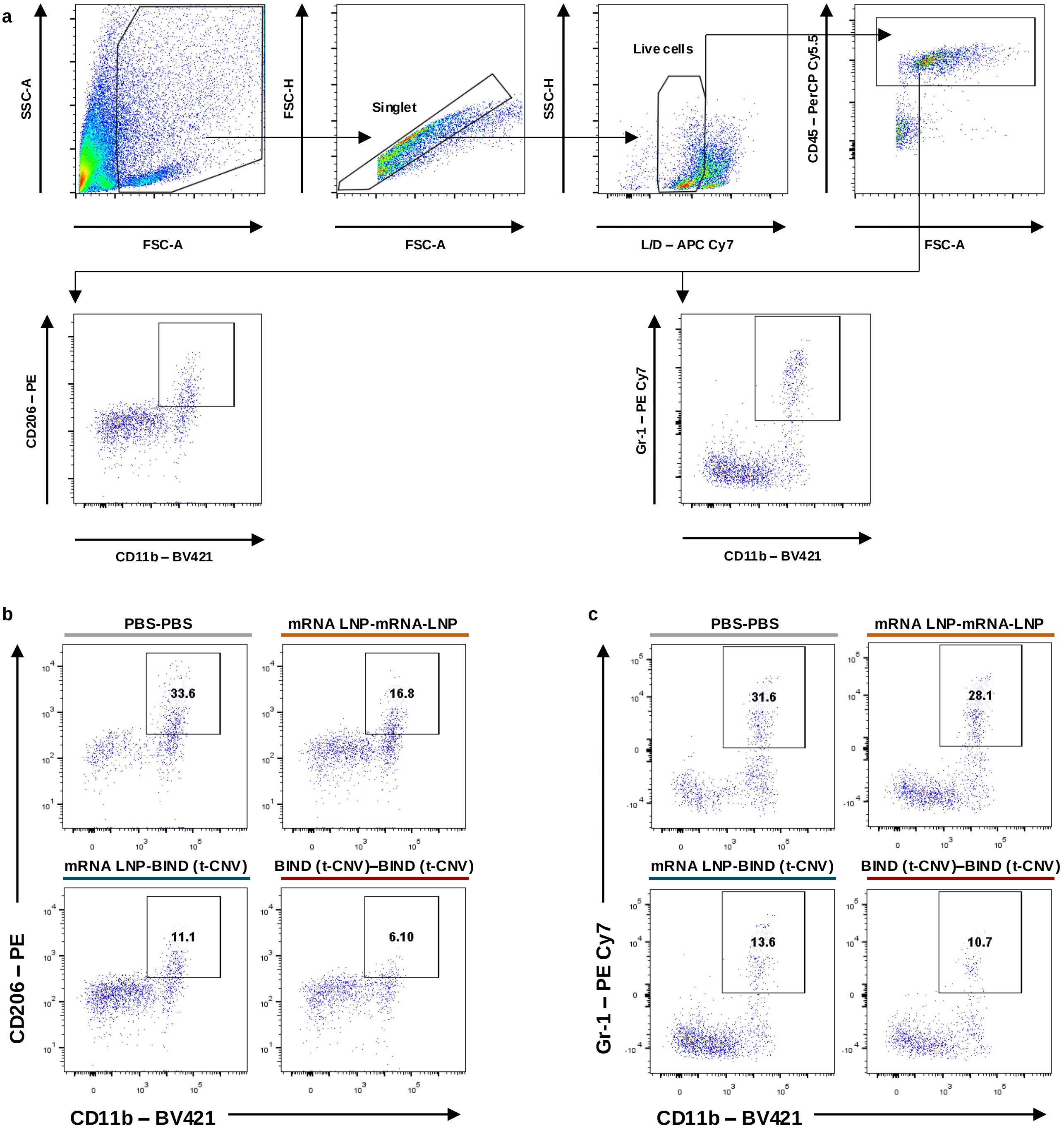

Figure S36. a, Flow cytometry gating strategy for Figure 6a illustrating the analysis of immunosuppressive cells *in vivo* in the B16-OVA tumor model. Populations of MDSCs ($CD11b^+Gr\text{-}1^+$ gated in $CD45^+$) and populations of M2 macrophages ($CD11b^+F4/80^+CD206^+$ gated in $CD45^+$) in the TME. The gating strategy for all samples was set to remove large clumps or aggregates of cells (FSC-H and FSC-A gating), cell debris, and dead cells (live/dead gating). **b-c,** Representative dot plots for Figure 6a showing M2 macrophages **(b)** and MDSCs **(c)** in the TME.

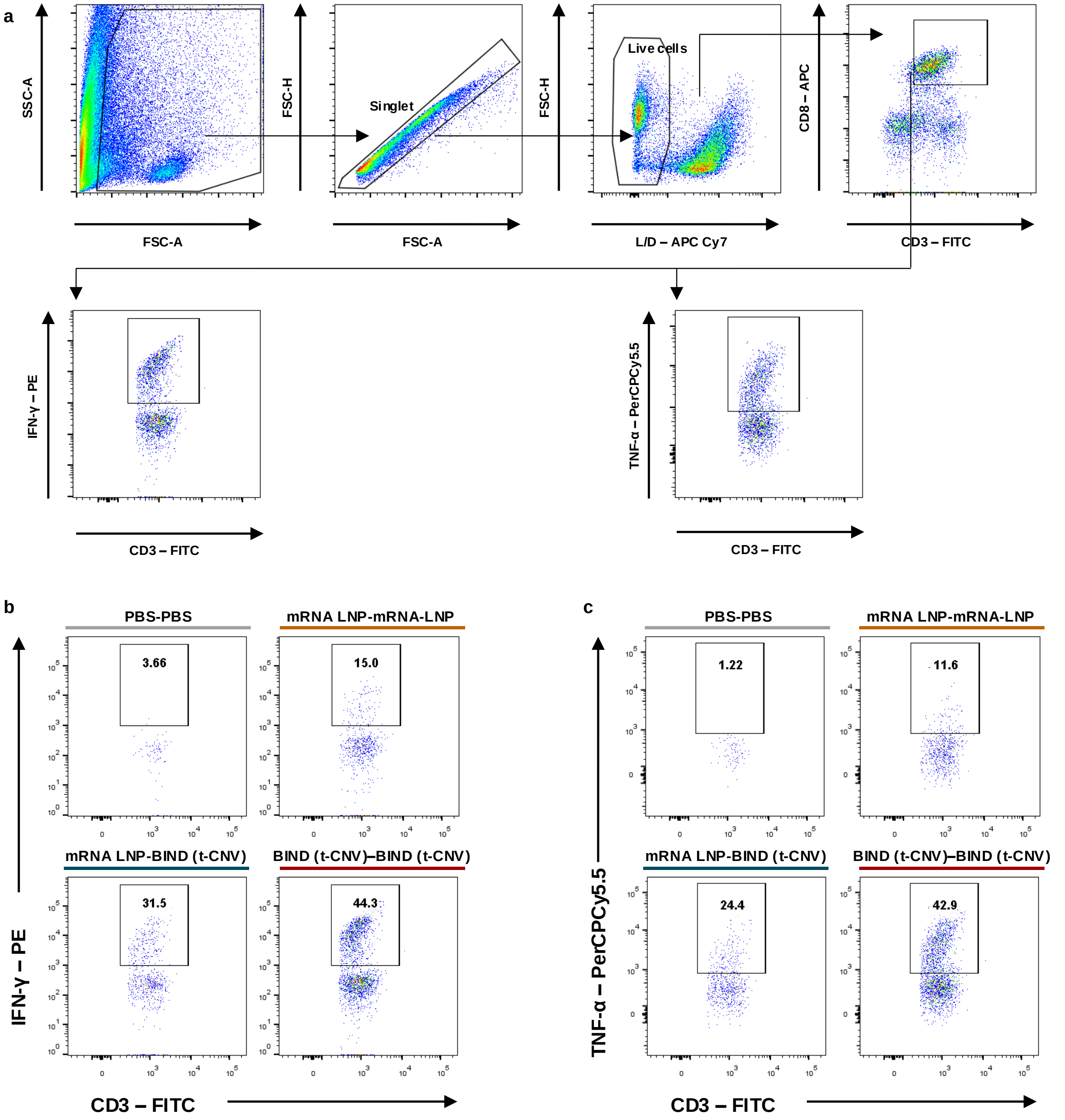

Figure S37. a, Flow cytometry gating strategy for Figure 6a illustrating the populations of IFN-γ^+ and TNF-α^+ producing CD8$^+$ T cells (CD3$^+$CD8$^+$) in the B16-OVA tumor model. The gating strategy for all samples was set to remove large clumps or aggregates of cells (FSC-H and FSC-A gating), cell debris, and dead cells (live/dead gating). **b-c,** Representative dot plots for Figure 6a showing the antigen-specific CD8$^+$ T cells in IFN-γ producing CD8$^+$ T cells **(b)** and TNF-α producing CD8$^+$ T cells **(c)** in the TME after SIINFEKL peptide-restimulation and incubation with GolgiPlug for 6 h.

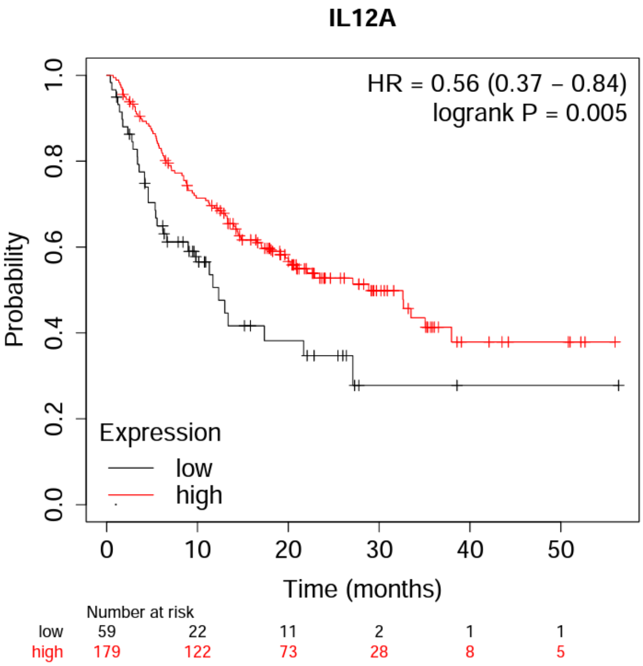

Low expression cohort (months)
12.33

High expression cohort (months)
28.85

Restrict to treatment...

Anti-PD-1 treatment:	Pembrolizumab only
Anti-PD-L1 treatment:	not used
Anti-CTLA-4 treatment:	not used
Sample acquisition:	all

Figure S38. Overall survival analysis to determine associations between the expression level of IL12A genes in anti-PD-1 (Pembrolizumab)-treated patients from the KM plotter (https://kmplot.com/analysis/). Patients with high IL12A gene expression exhibited increased responsiveness to anti-PD-1 therapy. The durable secretion of IL-12(p70) from BIND (t-CNV) suggests its potential as a synergistic partner with anti-PD-1 for enhanced therapeutic efficacy.

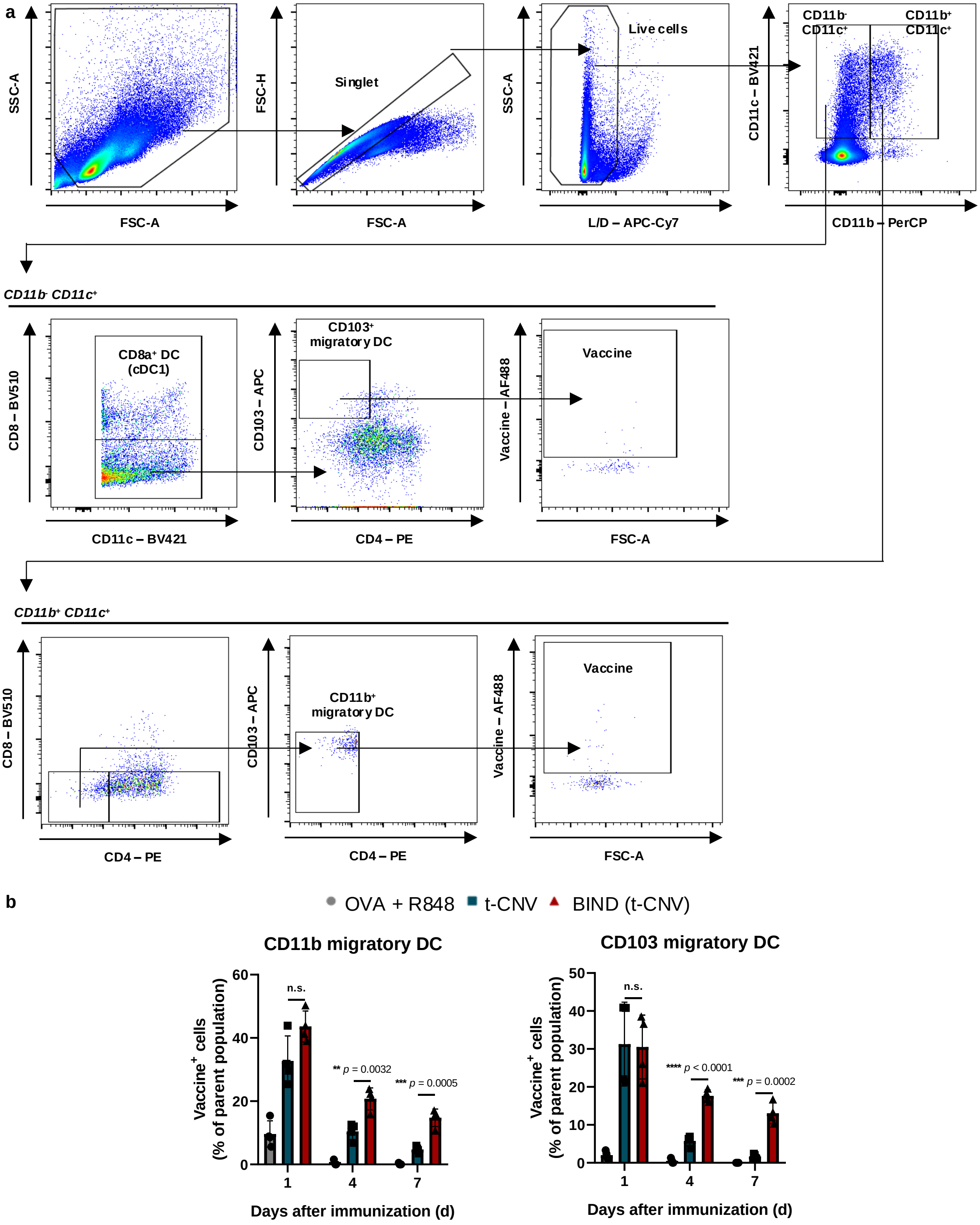

Figure S39. a, Flow cytometry gating strategy for determining the kinetics of the percentage of vaccine$^+$ APCs in the TDLN. CD11b migratory DCs (CD11b$^+$CD11c$^+$CD8$^-$CD4$^-$CD103$^-$) and CD103 migratory DCs (CD11b$^-$CD11c$^+$CD8$^-$CD4$^-$CD103$^+$). The gating strategy for all samples was set to remove large clumps or aggregates of cells (FSC-H and FSC-A gating), cell debris, and dead cells (live/dead gating). **b,** *In vivo* percentage of vaccine$^+$ antigen-presenting cells (APCs) in the TDLN of B16-OVA-tumor bearing mice 1, 4 or 7 days after peritumoral injection of the indicated fluorescently-labelled samples. APCs were defined as CD11b migratory DCs (CD11b$^+$CD11c$^+$CD8$^-$CD4$^-$CD103$^-$), and CD103 migratory DCs (CD11b$^-$CD11c$^+$CD8$^-$CD4$^-$CD103$^+$) (n = 3).

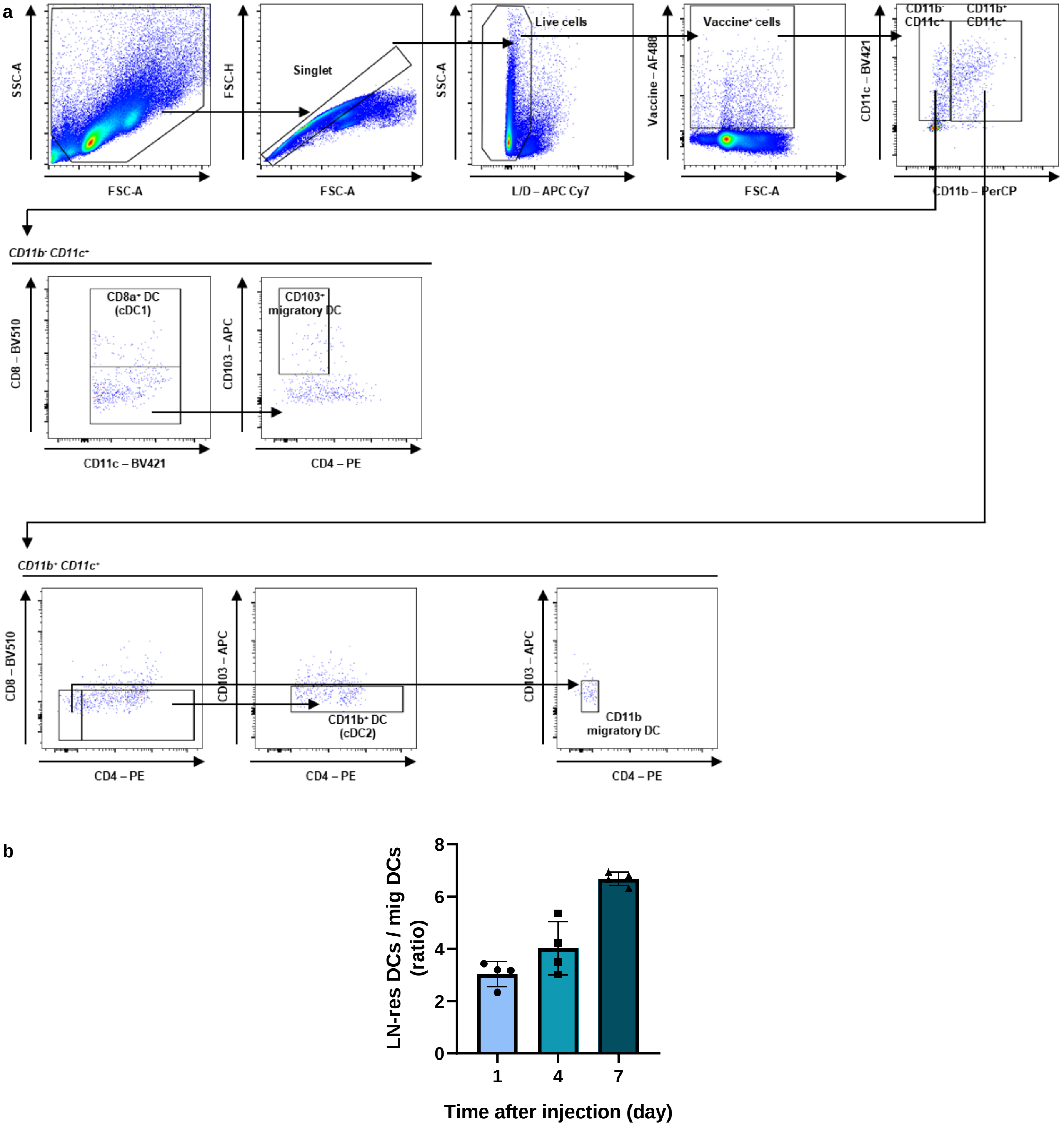

Figure S40. a, Flow cytometry gating strategy for determining the percentage of APCs in vaccine⁺ cells in the TDLN. cDC1s (CD11b⁻CD11c⁺CD8⁺), cDC2s (CD11b⁺CD11c⁺CD8⁻CD4⁺CD103⁻), CD11b⁺ migratory DCs (CD11b⁺CD11c⁺CD8⁻CD4⁻CD103⁻) and CD103⁺ migratory DCs (CD11b⁻CD11c⁺CD8⁻CD4⁻CD103⁺). The gating strategy for all samples was set to remove large clumps or aggregates of cells (FSC-H and FSC-A gating), cell debris, and dead cells (live/dead gating). **b,** *In vivo* ratio of LN resident DCs (cDC1s and cDC2s) to migratory DCs (CD11b⁺ migratory DCs and CD103⁺ migratory DCs) in vaccine⁺ cells in the TDLN of B16-OVA-tumor bearing mice. In the initial days (day 1 and day 4), vaccine⁺ migratory DCs were dominant which we attribute to the vaccine depot maintained at the local injection site for the first 1-4 days, as observed in Figure 3i via IVIS imaging. By day 7, vaccine⁺ LN resident DCs become dominant. This can be explained by intra-LN penetration through the conduit system, which occurs after the vaccine dissociates from the subcapsular sinus (SCS) as observed in Figure 4d.

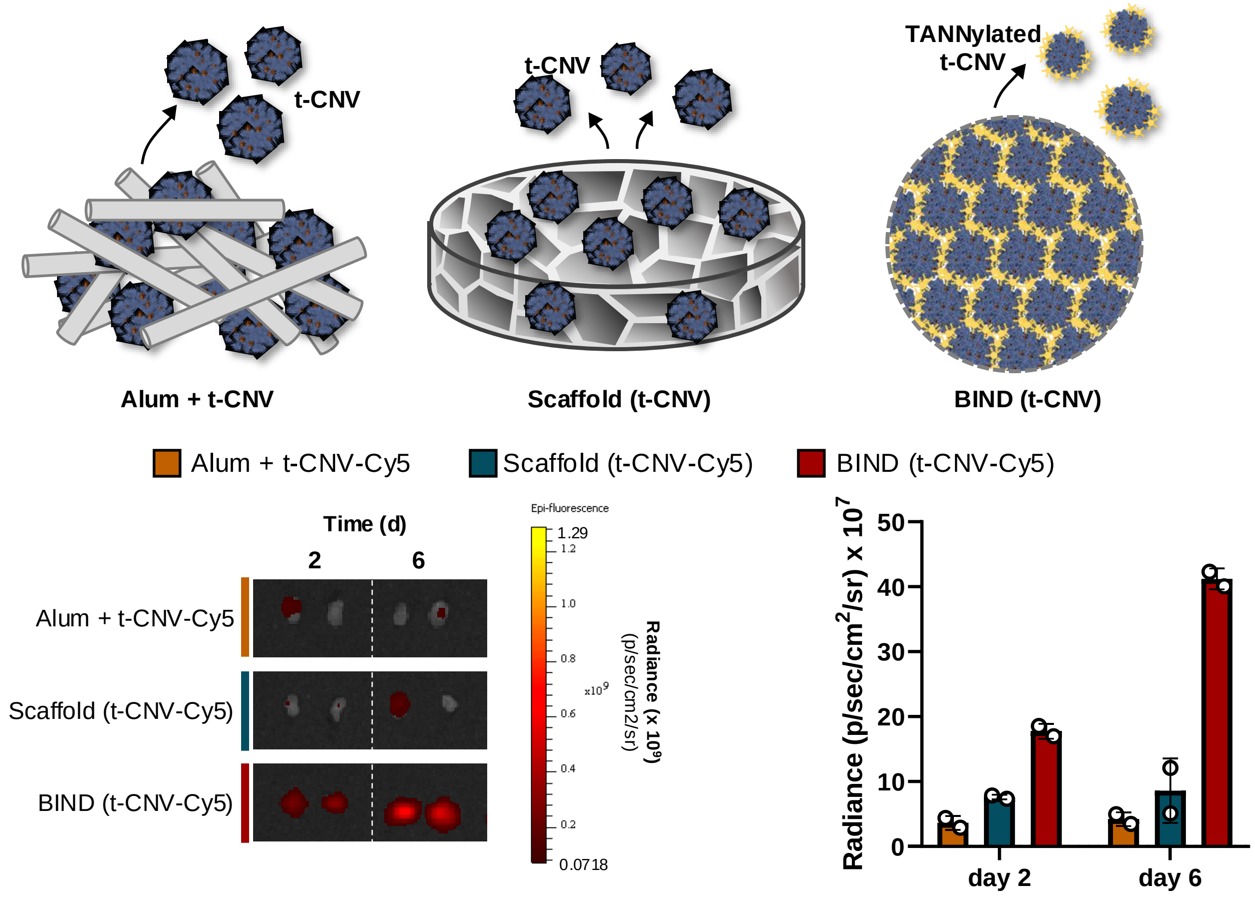

Figure S41. Comparison of the delivery efficacy of nanovaccines to the LNs via conventional local retention delivery systems and the BIND system. To evaluate the effectiveness of durable LN delivery, t-CNV-Cy5 was incorporated into three different local tension delivery systems: alum, scaffold and BIND. Each group was subcutaneously injected with the indicated samples, and dLNs were collected 2 or 6 days after the injection. The fluorescence intensity of the dye in the dLN was measured using an IVIS (n = 2). The term “Scaffold” refers to a polymer-based scaffold fabricated through a combination of methacrylate-modified hyaluronic acid and methacrylate-modified oxidized hyaluronic acid, with a pore size ranging from 50 to 100 μm.

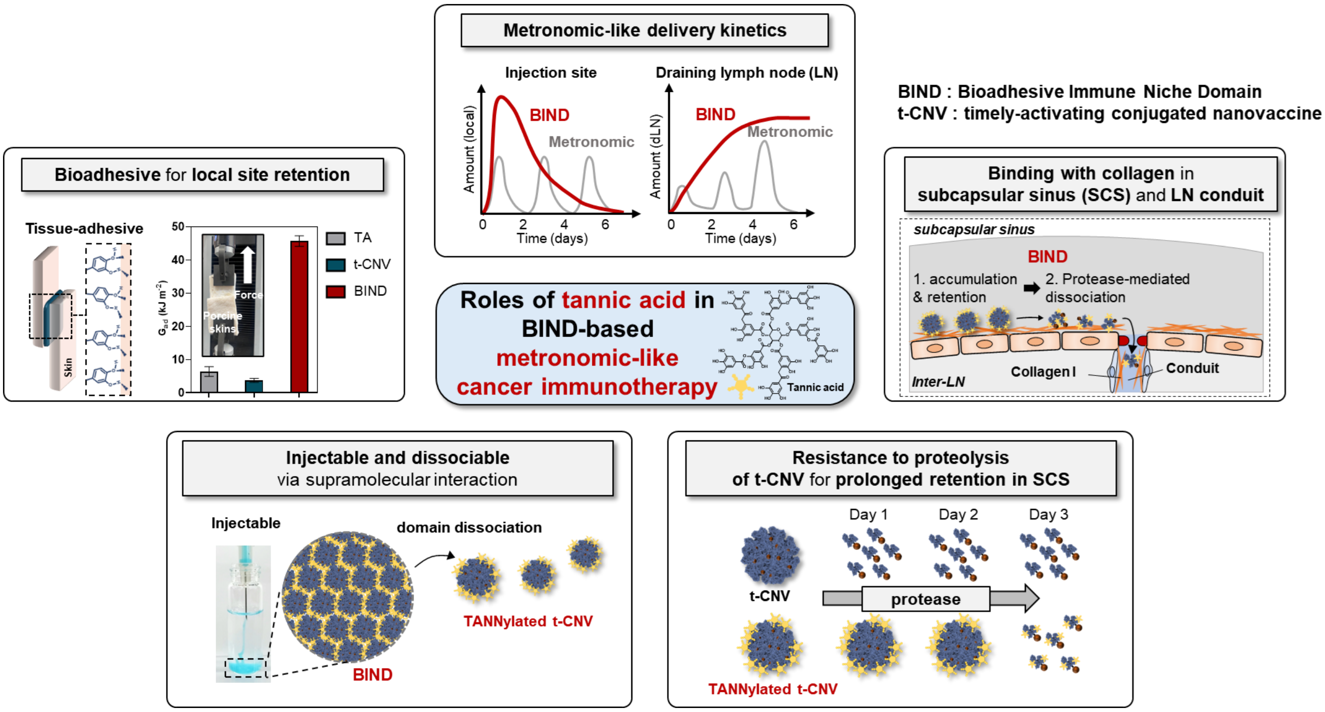

Figure S42. Schematic of multifunctional roles of tannic acid in metronomic-like cancer immunotherapy.

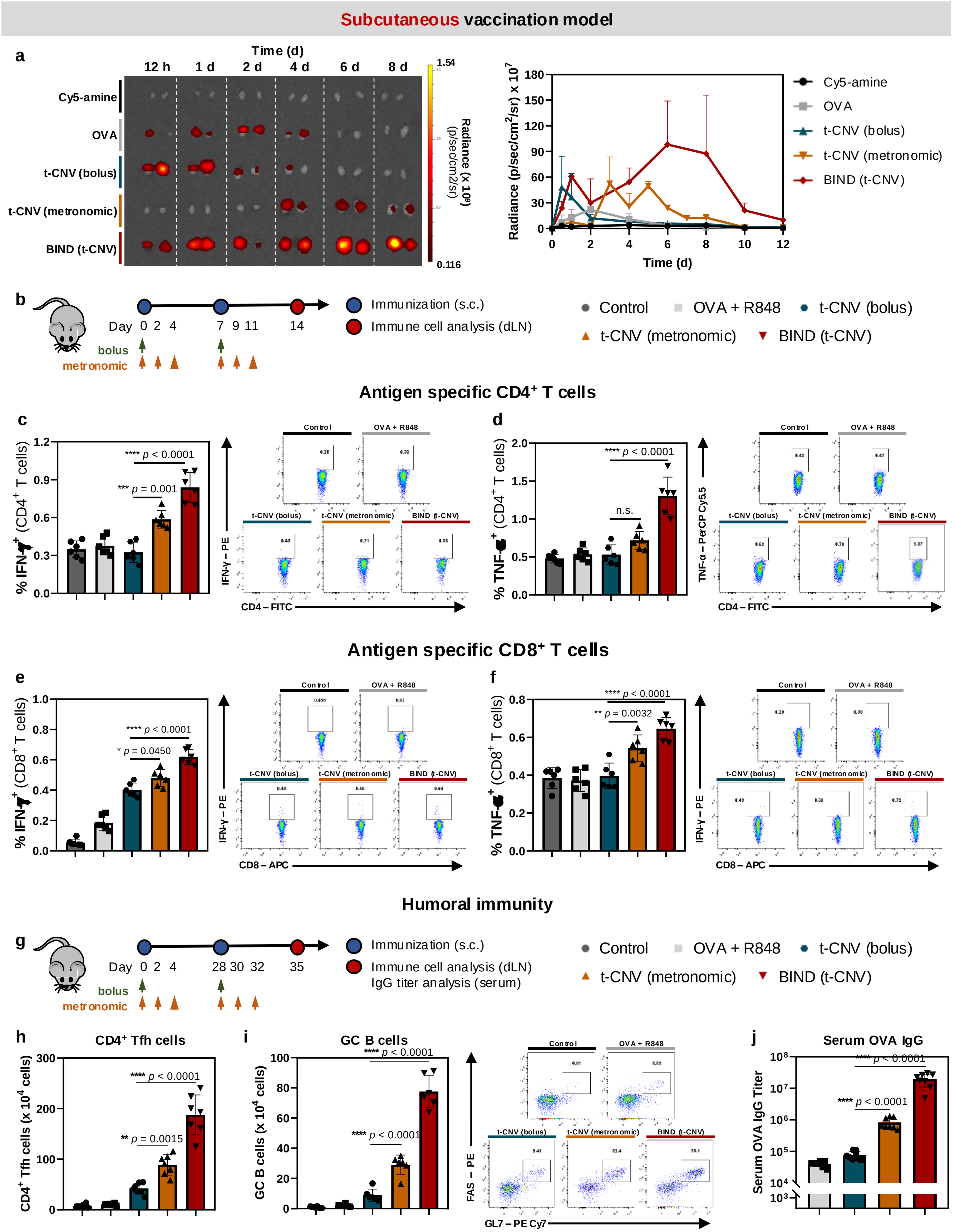

Subcutaneous vaccination model
a
Time (d)
12 h
1 d
2 d
4 d
6 d
8 d
Cy5-amine
OVA
t-CNV (bolus)
t-CNV (metronomic)
BIND (t-CNV)
1.54
0.116
Radiance (x 10⁹) (p/sec/cm2/sr)
Radiance (p/sec/cm²/sr) x 10⁷
Time (d)
Cy5-amine
OVA
t-CNV (bolus)
t-CNV (metronomic)
BIND (t-CNV)
b
Day 0 2 4 7 9 11 14
bolus
metronomic
Immunization (s.c.)
Immune cell analysis (dLN)
Control
OVA + R848
t-CNV (bolus)
t-CNV (metronomic)
BIND (t-CNV)
Antigen specific CD4⁺ T cells
c
% IFN-γ⁺ (CD4⁺ T cells)
**** p < 0.0001
*** p = 0.001
IFN-γ – PE
CD4 – FITC
d
% TNF-α⁺ (CD4⁺ T cells)
**** p < 0.0001
n.s.
TNF-α – PerCP Cy5.5
CD4 – FITC
Antigen specific CD8⁺ T cells
e
% IFN-γ⁺ (CD8⁺ T cells)
**** p < 0.0001
* p = 0.0450
IFN-γ – PE
CD8 – APC
f
% TNF-α⁺ (CD8⁺ T cells)
**** p < 0.0001
** p = 0.0032
IFN-γ – PE
CD8 – APC
Humoral immunity
g
Day 0 2 4 28 30 32 35
bolus
metronomic
Immunization (s.c.)
Immune cell analysis (dLN)
IgG titer analysis (serum)
Control
OVA + R848
t-CNV (bolus)
t-CNV (metronomic)
BIND (t-CNV)
h
CD4⁺ Tfh cells
CD4⁺ Tfh cells (x 10⁴ cells)
**** p < 0.0001
** p = 0.0015
i
GC B cells
GC B cells (x 10⁴ cells)
**** p < 0.0001
**** p < 0.0001
FAS – PE
GL7 – PE Cy7
j
Serum OVA IgG
Serum OVA IgG Titer
**** p < 0.0001
**** p < 0.0001

Figure S43. Dynamic immune modulation in the dLNs by BIND (t-CNV) after subcutaneous vaccination. **a,** IVIS images and average radiance of fluorescent signal in the dLN over time depicting the sustained vaccine availability of BIND. The indicated samples were subcutaneously administrated to E.G7-OVA tumor-bearing C57BL/6 mice (n = 2). **b-f,** Populations of antigen-specific $CD4^+$ T cells and $CD8^+$ T cells in dLNs after subcutaneous vaccination for two times with a one-week interval. OVA (1.4 mg), R848, and Trojan TLR7/8a (22.5 nmol) were subcutaneously injected. Percentages and representative dot plots of IFN-γ^+ **(c)** and TNF-α^+ **(d)** gated in $CD3^+CD4^+$ T cells in the dLN after SIINFEKL peptide-restimulation and incubation with GolgiPlug for 6 h (n = 6). Percentage and representative dot plot of IFN-γ^+ **(e)** and TNF-α^+ **(f)** gated in $CD3^+CD8^+$ T cells in dLN after SIINFEKL peptide-restimulation and incubation with GolgiPlug for 6 h (n = 6). **g-i,** Humoral immunity elicited in the dLN after subcutaneous vaccination for two times with a one-week interval. OVA (1.4 mg), R848, and Trojan TLR7/8a (22.5 nmol) were subcutaneously injected. Cell numbers and representative dot plots of follicular helper $CD4^+$ T cells (PD-1^+ $CXCR5^+$ gated in $CD4^+$) (n = 7) **(h)** and GC B cells (GL-7^+ FAS^+ gated in $B220^+$) (n = 6) **(i)**. Serum OVA IgG titer (n = 8) **(j)**. All data are presented as the mean ± s.d. Statistical significance was evaluated by one-way ANOVA with Tukey's multiple comparison test in **c-f** and **h-j**. *P* values: NS, not significant; *$P<0.05$, **$P<0.01$, ***$P<0.001$, ****$P<0.0001$.

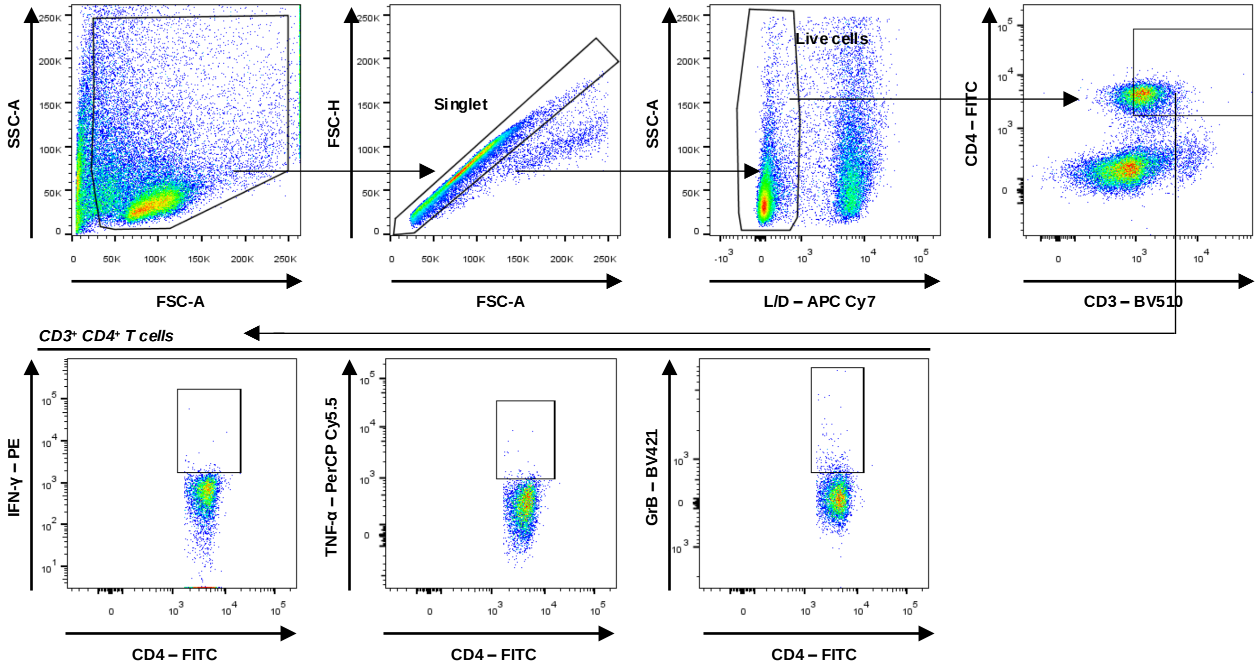

Figure S44. Flow cytometry gating strategy for Figure S43c,d, illustrating the populations of IFN-γ^{+}, TNF-α^{+} or GrB^{+} producing CD4^{+} T cells (CD3^{+}CD4^{+}). The gating strategy for all samples was set to remove large clumps or aggregates of cells (FSC-H and FSC-A gating), cell debris, and dead cells (live/dead gating).

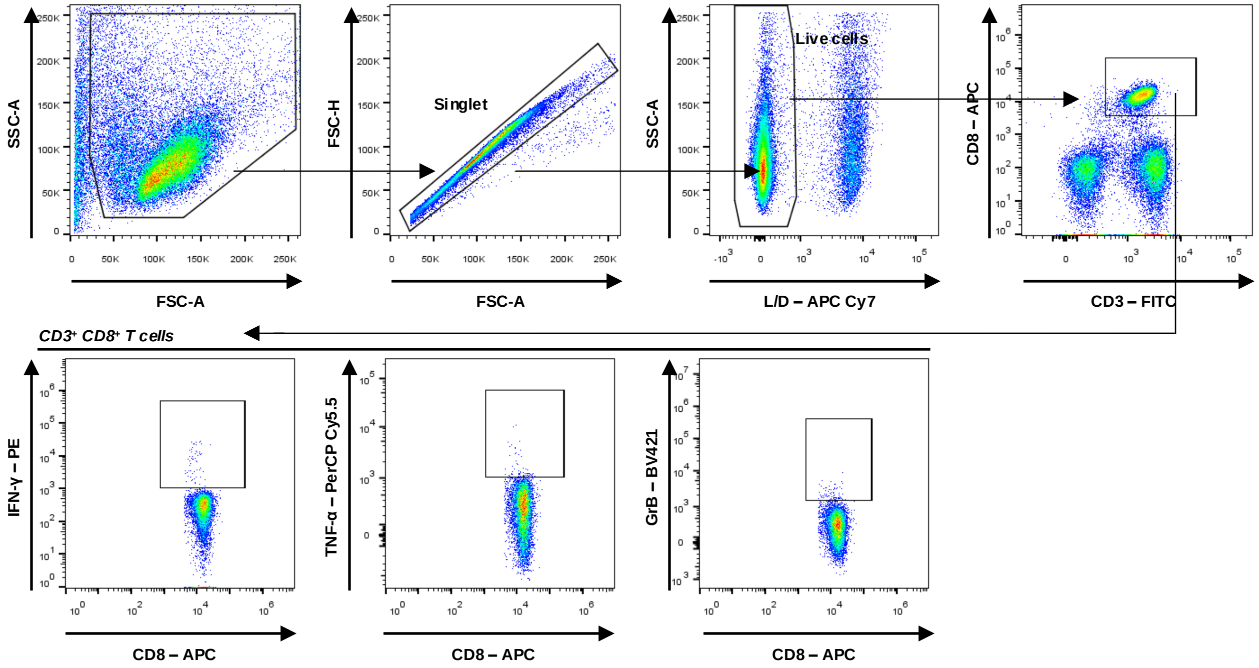

Figure S45. Flow cytometry gating strategy for Figure S43e,f illustrating the populations of IFN-γ$^+$, TNF-α$^+$ or GrB$^+$ producing CD8$^+$ T cells (CD3$^+$CD8$^+$). The gating strategy for all samples was set to remove large clumps or aggregates of cells (FSC-H and FSC-A gating), cell debris, and dead cells (live/dead gating).

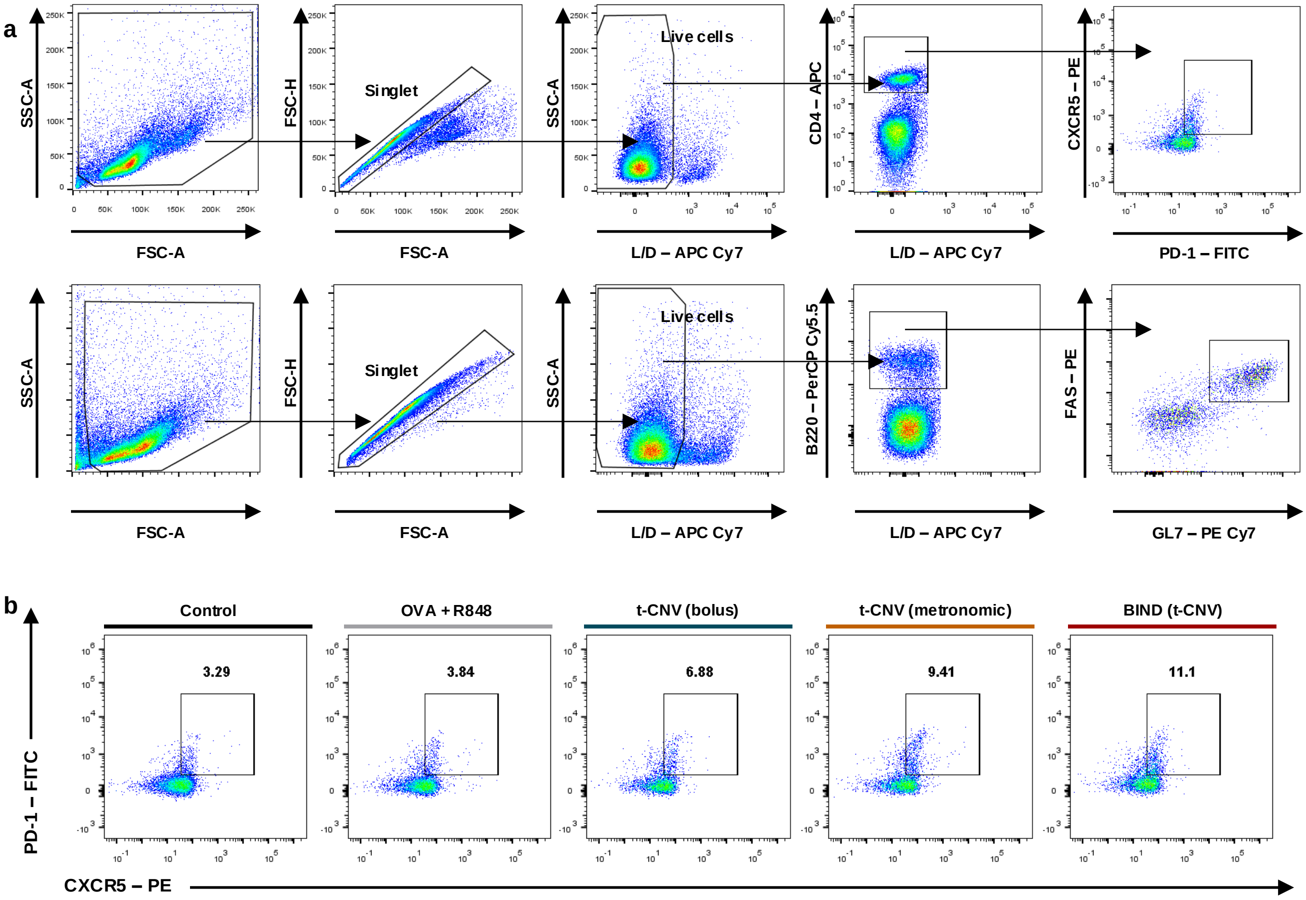

Figure S46. a, Flow cytometry gating strategy for Figure S43h,i illustrating follicular helper $CD4^+$ T cells and GC B cells. The gating strategy for all samples was set to remove large clumps or aggregates of cells (FSC-H and FSC-A gating), cell debris, and dead cells (live/dead gating). **b,** Representative dot plots for Figure S43h showing follicular helper $CD4^+$ T cells in the dLN.

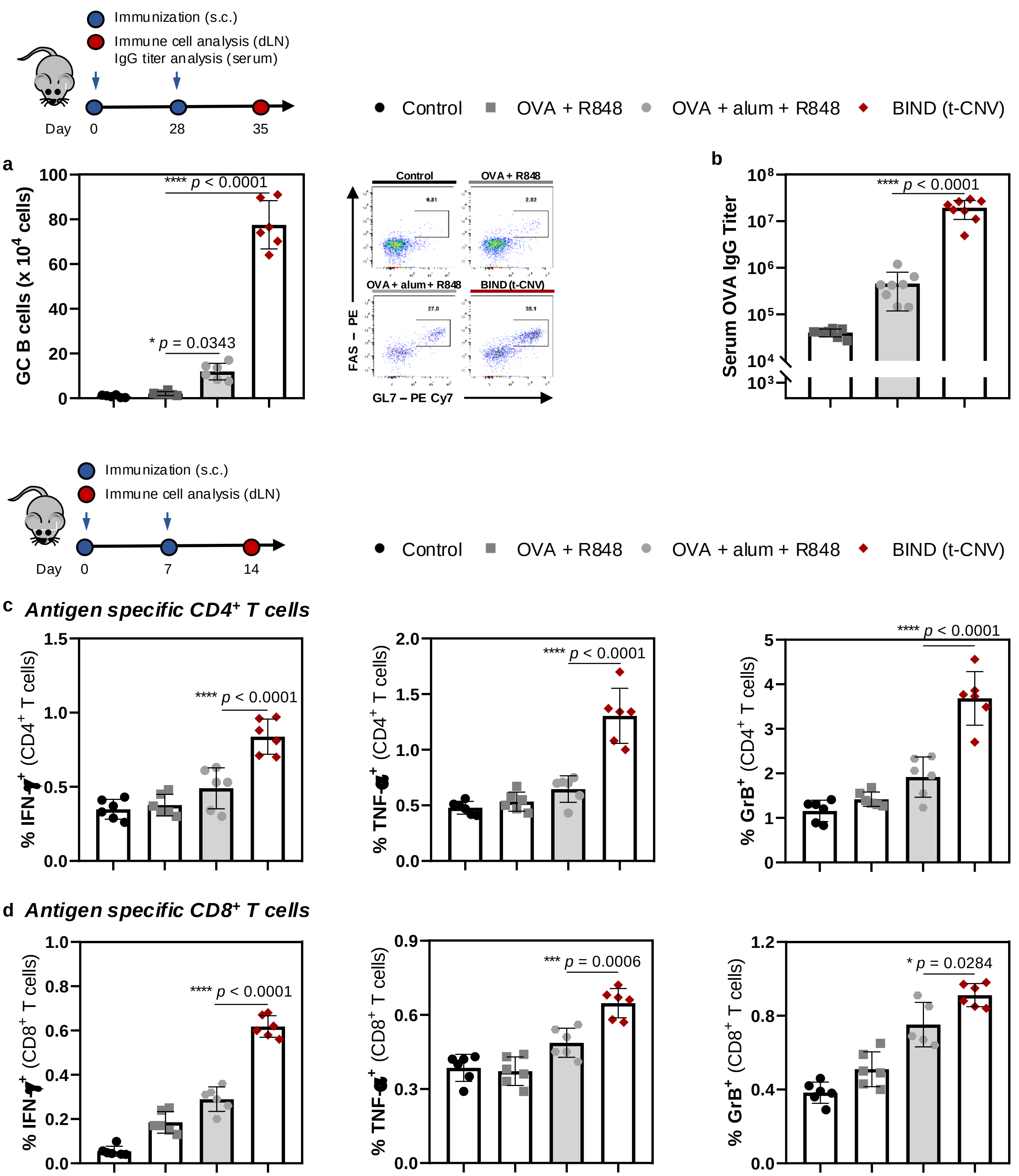
Immunization (s.c.)
Immune cell analysis (dLN)
IgG titer analysis (serum)
Day 0 28 35
Control
OVA + R848
OVA + alum + R848
BIND (t-CNV)
a
GC B cells (x 10^4 cells)
**** p < 0.0001
* p = 0.0343
Control
OVA + R848
OVA + alum + R848
BIND (t-CNV)
FAS – PE
GL7 – PE Cy7
b
Serum OVA IgG Titer
**** p < 0.0001
Immunization (s.c.)
Immune cell analysis (dLN)
Day 0 7 14
Control
OVA + R848
OVA + alum + R848
BIND (t-CNV)
c Antigen specific CD4+ T cells
% IFN-γ+ (CD4+ T cells)
**** p < 0.0001
% TNF-α+ (CD4+ T cells)
**** p < 0.0001
% GrB+ (CD4+ T cells)
**** p < 0.0001
d Antigen specific CD8+ T cells
% IFN-γ+ (CD8+ T cells)
**** p < 0.0001
% TNF-α+ (CD8+ T cells)
*** p = 0.0006
% GrB+ (CD8+ T cells)
* p = 0.0284

Figure S47. Vaccination efficacy of BIND (t-CNV) compared to that of an alum-based depot system (OVA+alum+R848). OVA (1.4 mg), R848, Trojan TLR7/8a (22.5 nmol), and alum (140 μg) were subcutaneously injected. **a,** Cell number and representative dot plot of GC B cells (GL-7$^+$ FAS$^+$ gated in B220$^+$) (n = 6). **b,** Serum OVA IgG titre (n = 6). **c,** Percentage of antigen-specific CD4$^+$ T cells in the dLN after SIINFEKL peptide-restimulation and incubation with GolgiPlug for 6 h (n = 6). **d,** Percentage of antigen-specific CD8$^+$ T cells in the dLN after SIINFEKL peptide-restimulation and incubation with GolgiPlug for 6 h (n = 5-6). All data are presented as the mean ± s.d. Statistical significance was evaluated by one-way ANOVA with Tukey's multiple comparison test in **a-d**. *P* values: NS, not significant; **P*<0.05, ***P*<0.01, ****P*<0.001, *****P*<0.0001.

Gene	Sense primer	Antisense primer
**GILT**	GGCTGATGGTGATGGAAATC	TTCAGTCTACACTCCAGCTC
**GAPDH**	TCCATGCCATCACTGCCACC	CAGTGAGCTTCCCGTTCAGC

Table S1. Sequences of primers for real-time PCR amplification.

Antibody	Manufacturer	Clone	Catalog number
LN immunofluorescence			
Alexa Fluor 488 anti-mouse CD3 Antibody	Biolegend	17A2	100210
Collagen I Monoclonal Antibody (COL-1)	Invitrogen	COL-1	MA1-26771
Goat anti-Mouse IgG (H+L) Highly Cross-Absorbed Secondary Antibody, Alexa Fluor Plus™ 405	Invitrogen		A48255
In vivo antibody injection			
InvivoMAb anti-mouse PD-1	BioXcell	RMP1-14	BE0146

Table S2. List of antibodies used for fluorescence imaging and in vivo antibody injection.

Antibody	Manufacturer	Clone	Catalog number
Flow Cytometry			
PerCP/Cyanine 5.5 anti-mouse CD45	BioLegend	30-F11	103132
Brilliant Violet 510™ anti-mouse CD3	BioLegend	17A2	100234
FITC anti-mouse CD3	BioLegend	17A2	100204
APC anti-mouse CD3	BioLegend	17A2	100236
FITC anti-mouse CD4	BioLegend	GK1.5	100406
APC anti-mouse CD4	BioLegend	GK1.5	100412
PE anti-mouse CD4	BioLegend	RM4-5	100512
APC anti-mouse CD8a	BioLegend	53-6.7	100712
Brilliant Violet 510™ anti-mouse CD8a	BioLegend	53-6.7	100752
PE anti-mouse IFN-γ	BioLegend	W18272D	163504
Brilliant Violet 421™ anti-human/mouse Granzyme B	BioLegend	QA18A28	396414
PerCP/Cyanine5.5 anti-mouse TNF-α	BioLegend	MP6-XT22	506322
APC anti-mouse TNF-α	BioLegend	MP6-XT22	506308
FITC anti-mouse CD279 (PD-1)	BioLegend	29F.1A12	135214
PerCP/Cyanine5.5 anti-mouse CD223 (LAG-3)	BioLegend	C9B7W	125212
PE anti-mouse CD366 (TIM-3)	BioLegend	B8.2C12	134004
APC anti-mouse NK 1.1	BioLegend	PK136	108710
PE hamster anti-mouse CD69	BD bioscience	H1.2F3	553237
PerCP anti-mouse/human CD11b	BioLegend	M1/70	101230
Brilliant Violet 421™ anti-human/mouse CD11b	BioLegend	M1/70	101236
Brilliant Violet 510™ anti-mouse CD11c	BioLegend	N418	117338
Brilliant Violet 421™ anti-mouse CD11c	BioLegend	N418	117330
PE anti-mouse F4/80	BioLegend	BM8	123110
FITC anti-mouse F4/80	BioLegend	BM8	123108
APC anti-mouse CD103	BioLegend	2E7	121414
APC anti-mouse Ly6G	BioLegend	1A8	127614
PerCP anti-mouse/human CD45R/B220	BioLegend	RA3-6B2	103234
APC anti-mouse IgD	BioLegend	11-26c.2a	405714
PE anti-mouse CD95 (Fas)	BioLegend	SA367H8	152608
PE/Cyanine7 anti-mouse/human GL7 Antigen	BioLegend	GL7	144620
Biotin anti-mouse CD185 (CXCR5)	BioLegend	L138D7	145510

Table S3. List of antibodies used for flow cytometry analysis.

Group	Force (N)	E (modulus) (kPa)
Tannic acid	0.2883	32.06
	0.2336	38.78
	0.202	43.98
t-CNV	0.3334	57.03
	0.2619	40.38
	0.1488	6.33
BIND (t-CNV)	0.9731	60.52
	0.7548	35.38
	0.8817	69.13

Table S4. List of the value of F and E of Figure 3h.
